# Distinct diurnal temperature rhythm patterns in critical illness myopathy: secondary analysis of two prospective trials

**DOI:** 10.1186/s13613-025-01582-5

**Published:** 2025-10-27

**Authors:** D. Mewes, S. Weber-Carstens, K. Rubarth, S. D. Boie, C. Spies, A. Kramer, J. Fielitz, T. Wollersheim, B. Ananthasubramaniam, F. Braune, L. Hancke, L. Spies, F. Balzer, L. J. Engelhardt

**Affiliations:** 1 Department of Anesthesiology and Intensive Care Medicine, Charité – Universitätsmedizin Berlin, corporate member of Freie Universität Berlin and Humboldt-Universität zu Berlin, Augustenburger Platz 1, 13353 Berlin, Germany; 2 ECRC Experimental and Clinical Research Center, Charité – Universitätsmedizin Berlin, corporate member of Freie Universität Berlin and Humboldt-Universität zu Berlin, Lindenberger Weg 80, 13125 Berlin, Germany; 3 Institute of Biometry and Clinical Epidemiology, Charité – Universitätsmedizin Berlin, corporate member of Freie Universität Berlin and Humboldt-Universität zu Berlin, Charitéplatz 1, 10117 Berlin, Germany; 4 Institute of Medical Informatics, Charité – Universitätsmedizin Berlin, corporate member of Freie Universität Berlin and Humboldt-Universität zu Berlin, Charitéplatz 1, 10117 Berlin, Germany; 5 Laboratory of Chronobiology, Charité – Universitätsmedizin Berlin, corporate member of Freie Universität Berlin and Humboldt-Universität zu Berlin, Charitéplatz 1, 10117 Berlin, Germany; 6 Department of Internal Medicine B Cardiology, University Medicine Greifswald, Fleischmannstr. 41, 17475 Greifswald, Germany; 7 DZHK (German Center for Cardiovascular Research), Partner Site, Greifswald, Germany; 8 Institute for Theoretical Biology, Humboldt-Universität zu Berlin, Philippstr.13, 10115 Berlin, Germany; 9 Klinik für Anästhesie und Intensivmedizin, Universitätsklinikum Ruppin-Brandenburg, Ruppiner Kliniken GmbH, Medizinische Hochschule Brandenburg Theodor Fontane, Fehrbelliner Straße 38, 16816 Neuruppin, Germany; 10 Hasso-Plattner-Institute, University of Potsdam, Prof.-Dr.-Helmert-Straße 2–3, 14482 Potsdam, Germany; 11 Friede Springer Cardiovascular Prevention Center at Charité, Charité – Universitätsmedizin Berlin, corporate member of Freie Universität Berlin and Humboldt-Universität zu Berlin, Hindenburgdamm 30, Haus IIIA, 12203 Berlin, Germany; 12 Berlin Institute of Health at Charité – Universitätsmedizin Berlin, BIH Biomedical Innovation Academy, BIH Charité Junior Digital Clinician Scientist Program, Charitéplatz 1, 10117 Berlin, Germany

**Keywords:** Circadian rhythms, Diurnal rhythms, Circadian rhythm disruption, Body temperature, Temperature rhythm, Critical illness, Skeletal muscle atrophy, Critical illness myopathy, Muscle weakness

## Abstract

**Background:**

Critical illness myopathy (CIM) increases mortality and causes long-term disabilities. CIM is characterized by reduced muscle excitability, muscle atrophy, weakness, and impaired glucose metabolism. Functional circadian rhythms are important for skeletal muscle homeostasis. Circadian rhythms are often disrupted during critical illness in the Intensive Care Unit (ICU). This analysis investigates whether diurnal temperature rhythms differ in critically ill CIM compared to no-CIM patients.

**Methods:**

This is a secondary analysis of two prospective trials including critically ill patients with CIM (*n* = 32) or no-CIM (*n* = 30) based on electrophysiological tests. Diurnal body temperature rhythms were compared between CIM and no-CIM groups in reference to *n* = 16 participants included in a bed rest study. Cosinor analysis was performed to determine the rhythm parameters and classify into rhythm classes. Aggregated and longitudinal data were compared between groups using non-parametric tests. Rhythm parameters were correlated with muscle atrophy, weakness and insulin sensitivity.

**Results:**

CIM and no-CIM patients had severe multiorgan failure (median SOFA score 12 in both groups, *p* = 0.39). The temperature rhythm nadir timepoint was shifted in CIM patients (10:43 [09:21, 12:22]) and no-CIM (11:12 [09:43, 13:30]) compared to the healthy bed rest group (5:03 [3:22, 6:36]) *p* < 0.001. CIM patients showed lower temperature rhythm mesors than no-CIM patients (*p* = 0.041). The temperature rhythm amplitude was lower in both CIM and no-CIM patients compared to the healthy bed rest group (CIM: 0.3 °C [0.2, 0.4]; no-CIM: 0.2 °C [0.2, 0.3]; healthy bed rest: 0.5 °C [0.2, 0.6]; *p* < 0.01). Compared to no-CIM patients, CIM patients had higher temperature rhythm amplitudes (*p* = 0.021) and showed a less pronounced reduction in temperature rhythm amplitudes during ICU stay (*p* = 0.017). A higher temperature rhythm amplitude correlated negatively with *M. vastus lateralis* myocyte cross-sectional area.

**Conclusions:**

Heterogeneous phase shifts of diurnal temperature rhythms in CIM and no-CIM groups compared to healthy bed rest volunteers may indicate ICU-related circadian disruption. Suppression of temperature rhythm amplitude during ICU stay could represent an adaptive response to this disruption. Blunted amplitude suppression observed in CIM compared to no-CIM patients might reflect reduced adaptation, potentially contributing to muscle catabolism. This hypothesis-generating analysis underlines the need for mechanistic studies exploring circadian regulation in skeletal muscle during critical illness.

**Graphical Abstract:**

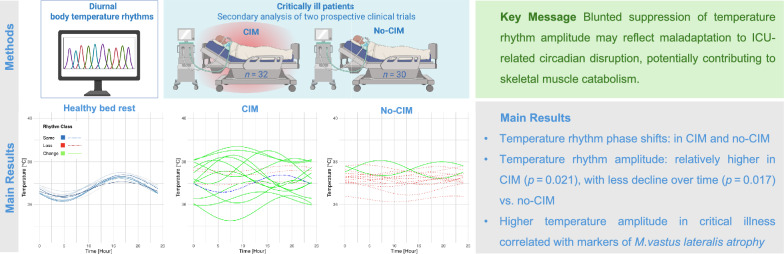

**Supplementary Information:**

The online version contains supplementary material available at 10.1186/s13613-025-01582-5.

## Introduction

Critically ill patients are susceptible to developing critical illness myopathy (CIM). CIM is characterized by reduced muscle membrane excitability, skeletal muscle atrophy, symmetric weakness, and loss of muscle function [1]. This devastating complication contributes to mortality and to long-term physical disability in the post-intensive care syndrome in survivors [1]. Critically ill patients also tend to have a circadian rhythm disruption due to their severe illness and the ICU environment, where light exposure, nutrition, and noise do not follow the typical day-night rhythm [2–5].

Diurnal body temperature is a widely used biomarker of circadian regulation [6, 7] that is routinely assessed and is feasible in critical illness [8]. Moreover, body temperature is of interest due to its bidirectional interaction with the skeletal muscle. Muscle function varies according to the time of day [9]. Exercise performance is typically better in the early evening, which correlates with the physiological body temperature peak [10]. In addition, muscle activity itself is a powerful synchronizer that helps restore diurnal temperature rhythms [11].

Functional circadian rhythms are important for skeletal muscle homeostasis [12–14]. In skeletal muscle, the molecular clock controls the expression of genes involved in the regulation of muscle mass and metabolism [12–15]. Disruption of the skeletal muscle clock causes muscle atrophy and impaired glucose metabolism [12, 16].

The phenotype of CIM shows striking similarities, including muscle atrophy, weakness, and impaired glucose metabolism [17]. Whether specific alterations in the diurnal temperature rhythm are associated with CIM has not been investigated. Addressing this knowledge gap, this analysis aims to compare diurnal body temperature rhythms between no-CIM and CIM patients from two prospective ICU trials [18, 19] in reference to healthy volunteers included in a bed rest study [20]. We hypothesize that CIM patients present with distinct diurnal temperature rhythm characteristics that differ from non-CIM critically ill patients and healthy controls.

## Results

This analysis included *n* = 32 CIM, *n* = 30 no-CIM patients, and *n* = 16 healthy bed rest volunteers. Baseline characteristics, clinical, histological and metabolic data in the CIM and no-CIM groups are shown in Table 1.

**Table 1:** Baseline characteristics

	CIM *n* = 32	No-CIM *n* = 30	*p*-value
Age [years]	64 [45; 71]	46 [40; 62]	*p* = 0.025
Male [n, %]	22 [69]	18 [60]	*p* = 0.65
Female [n, %]	10 [31]	12 [40]	
BMI [kg/m^2^]	27 [23; 31]	27 [24; 30]	*p* = 0.77
SOFA	12 [11; 14]	12 [9; 14]	*p* = 0.39
APACHE	23 [18; 29]	21 [16; 25]	*p* = 0.21
SAPS2	50 [38; 63]	49 [40; 58]	*p* = 0.56
Days with temperature ≥ 38 °C [n]	15 [7; 19]	14 [5; 15]	*p* = 0.27
Days until first awakening [n]	12 [9; 17]	11 [8; 19]	*p* = 0.42
MRC at first awakening	3.0 [2.8; 3.4]	3.8 [2.9; 4.1]	*p* = 0.10
MRC at discharge	3.8 [3.0; 4.1]	4.0 [3.4; 4.4]	*p* = 0.22
Day of *M.vastus lateralis* biopsy	18 [16; 21]	15 [14; 20]	*p* = 0.12
MCSA type I fibers [µm^2^]	3150 [2420; 3790]	3850 [2600; 4950]	*p* = 0.14
MCSA type IIa fibers [µm^2^]	2410 [1230; 3850]	3510 [2320; 4770]	*p* = 0.049
MCSA type IIb fibers [µm^2^]	2190 [1080; 2790]	3100 [2130; 3630]	*p* = 0.06
Insulin sensitivity index [(mg/min/kg)/mU/L)]	0.025 [0.019; 0.036]	0.030 [0.025; 0.044]	*p* = 0.24
Day of hyperinsulinemic-euglycemic clamp	19 [17; 22]	17 [14; 20]	*p* = 0.12

For the Cosinor analysis, 1 day was available per healthy bed rest volunteer, while 24 days [17; 31] per CIM patient, and 20 days [16; 27] per no-CIM patient were investigated. The R^2^ model fit was 0.88 [0.60; 0.95] in the healthy bed rest group*,* and 0.51 [0.41; 0.58] in the CIM compared to 0.50 [0.43; 0.57] in the no-CIM groups (*p* = 0.67).

**Fig. 1:**
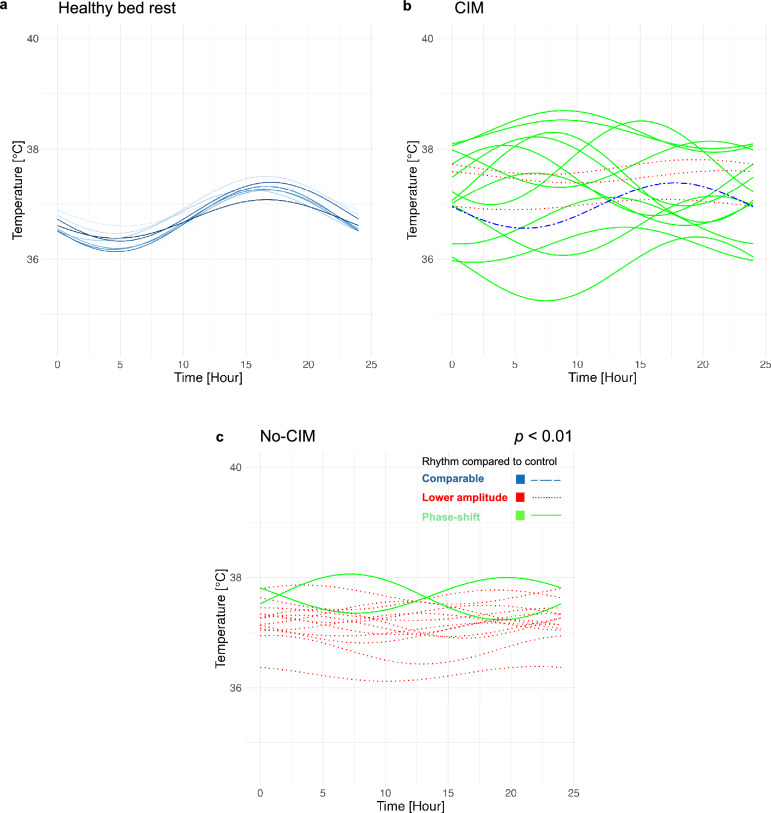
Diurnal temperature rhythms – Cosinor analysis. Diurnal temperature rhythms in CIM and no-CIM patients were classified in comparison to the healthy bed rest group: blue lines show comparable temperature rhythms; red flat dotted lines indicate lower temperature rhythm amplitudes; green lines indicate phase-shifted temperature rhythm nadirs with maintained amplitudes. **a** Healthy bed rest volunteers showed the physiological diurnal temperature rhythm, *n* = 16, raw data publicly available [20]. **b** CIM patients presented with phase-shifted, relatively higher-amplitude temperature rhythms than no-CIM patients; example shown for the day before discharge. CIM patients (*n* = 16) were more likely to be classified as having a phase shift compared to no-CIM patients (*n* = 16) on the day before ICU discharge (*p* = 0.01; Chi-square test). **c** In no-CIM patients, lower temperature rhythm amplitudes predominated on the day before ICU discharge. Data were presented at *ESICM Lives* [54]*. CIM* Critical Illness Myopathy

Cosinor analysis and temperature rhythm classification for exemplary ICU days are illustrated for the healthy bed rest group (Fig. 1a), and for the CIM (Fig. 1b) and no-CIM (Fig. 1c) groups (and in Fig.S1, Supplementary Material 1). The temperature rhythms in the majority of critically ill patients were classified into either a ‘lower amplitude’, or ‘phase-shifted’ category compared to healthy bed rest volunteers on all days of interest. Individual original temperature data and Cosinor overlays are shown in the Supplementary Material 2.

### Temperature rhythm phases were shifted in CIM and no-CIM patients compared to healthy bed rest volunteers.

**Fig. 2:**
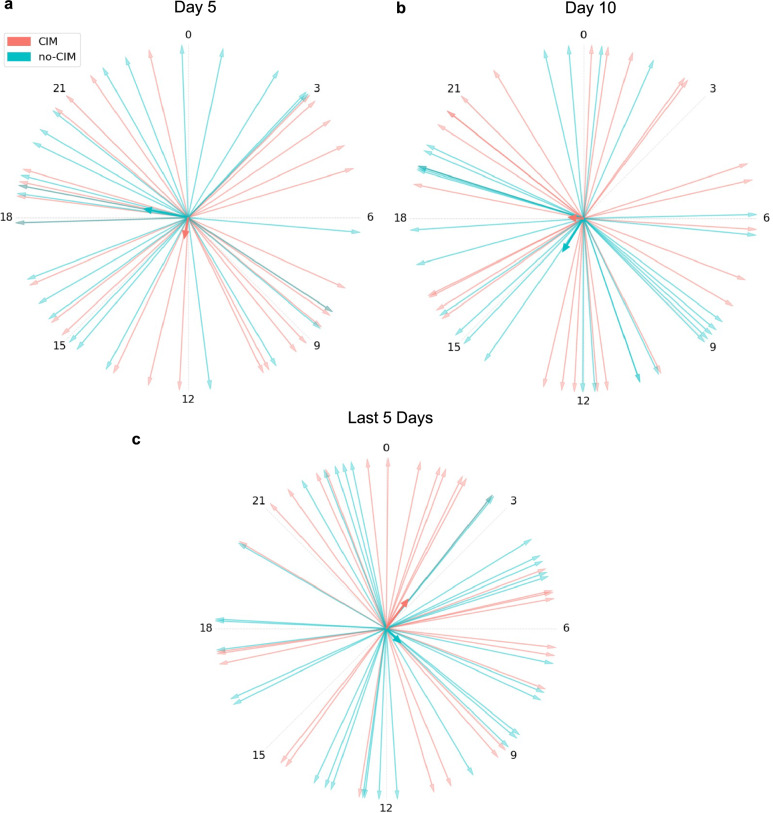
Rayleigh plot **a** ICU day 5 **b** ICU Day 10 **c** Last 5 ICU days with available temperature data: High variability in the timing of the temperature rhythm nadir was observed in both CIM and no-CIM groups, without a consistent phase direction. Each arrow represents the temperature rhythm nadir of an individual patient; the bold vector indicates the group average

**Fig. 3:**
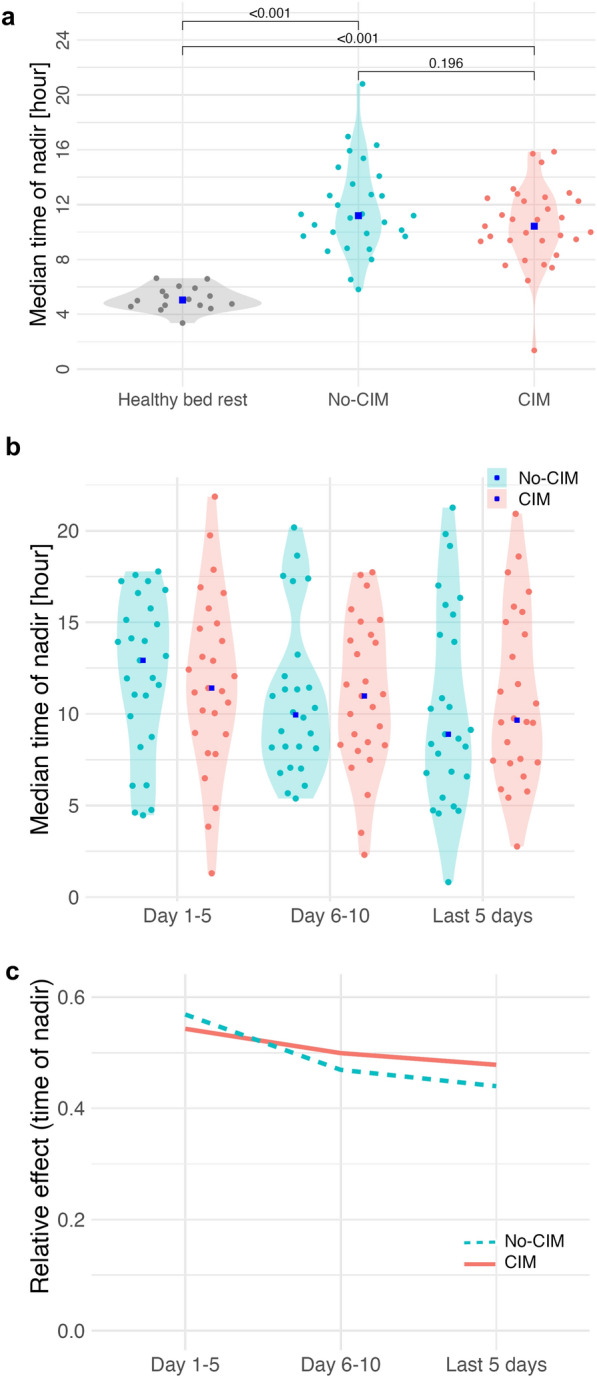
Time of temperature rhythm nadir (minimum phase). **a** Aggregated median time of nadir during ICU stay: healthy bed rest 05:03 [03:22, 06:36]; CIM 10:43 [09:21, 12:22]; no-CIM 11:12 [09:43, 13:30]. **b** Longitudinal trend of temperature rhythm nadir at ICU day 5, ICU day 10, and the last 5 ICU days with available data: no differences between CIM and no-CIM patients (group effect: *p* = 0.721); no significant change over time within groups (time effect: *p* = 0.243). **c** Relative effects demonstrated no interaction between group and time (interaction effect: *p* = 0.837). Brunner–Munzel analysis. *CIM* Critical Illness Myopathy

Healthy bed rest volunteers represented with the temperature rhythm nadir at 05:03 [03:22, 06:36], and the peak at 17:04 [15:23, 19:35], showing the physiological diurnal temperature rhythm (Fig. 1a) [20, 22, 23]. The circular Rayleigh plot illustrated a high interindividual variability in temperature rhythm nadir timing in CIM and no-CIM groups, without a consistent phase direction (Fig. 2). Compared to healthy bed rest volunteers, CIM and no-CIM patients had phase-shifted temperature rhythms (*p* < 0.01), with the aggregated median nadir at 10:43 [09:21, 12:22] in the CIM vs. at 11:12 [09:43, 13:30] in the no-CIM group (Fig. 3a) (*p* = 0.20).

In the longitudinal analysis across ICU time periods, temperature rhythm nadir time remained highly variable in both groups: no differences between CIM and no-CIM patients (group effect: *p* = 0.721), no significant changes over time within groups (time effect: *p* = 0.243), and no interaction between group and time (interaction effect: *p* = 0.837) were observed (Fig. 3b, c).

### Temperature rhythm mesors were higher in both CIM and no-CIM patients compared to healthy bead rest volunteers, but lower in CIM than in no-CIM patients.

**Fig. 4:**
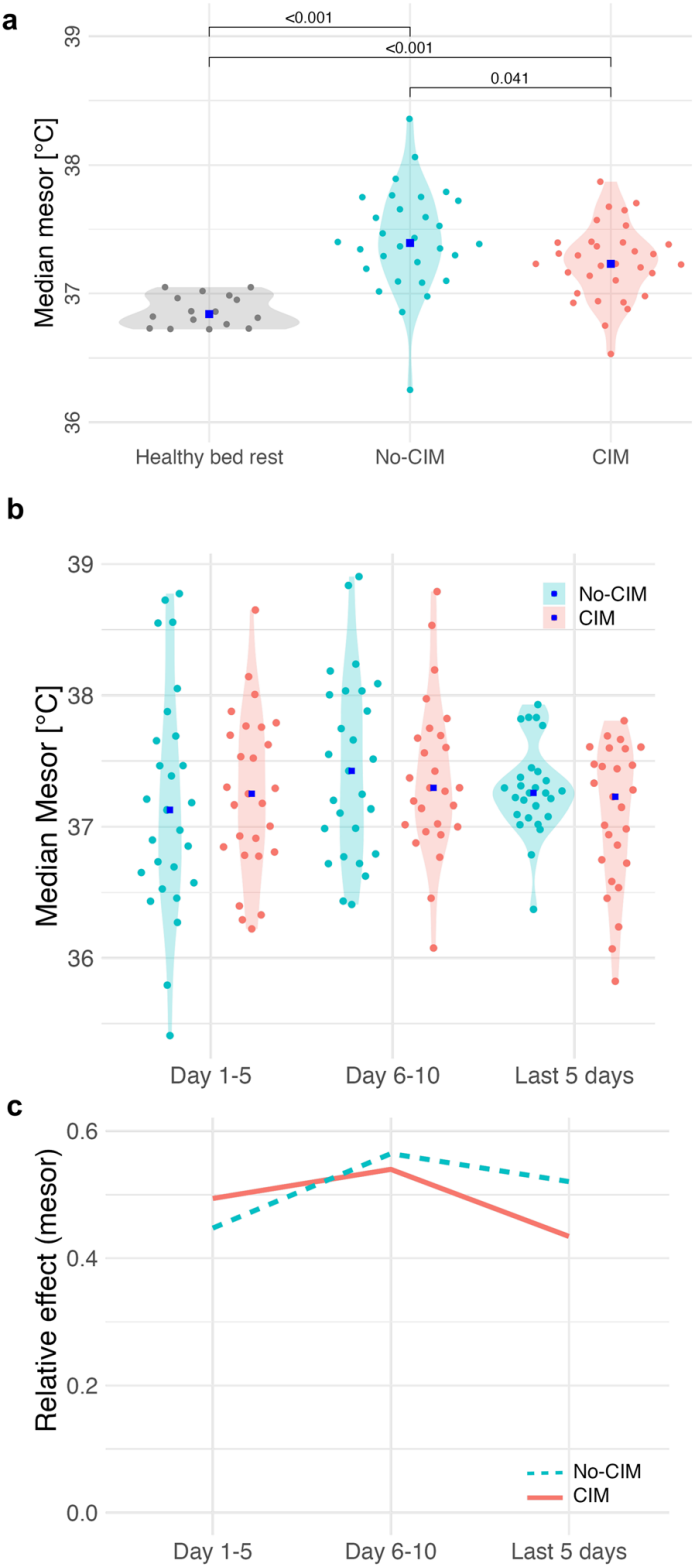
Temperature rhythm mesor. **a** Aggregated median mesor during ICU stay: healthy bed rest 36.8 °C [36.7; 37.1]; CIM 37.2 °C [37.1; 37.4]; no-CIM 37.4 °C [37.2; 37.7] (*p* = 0.041). **b** Longitudinal trend of mesor at ICU day 5, ICU day 10, and the last 5 ICU days with available data: no difference between CIM and no-CIM (group effect: *p* = 0.700); no significant change over time within groups (time effect: *p* = 0.181). **c** Relative effects indicated no interaction between group and time (interaction effect: *p* = 0.383). Brunner–Munzel analysis. *CIM* Critical Illness Myopathy

 The healthy bed rest group had a mesor of 36.8 °C [36.7; 37.1] (Fig. 4a). The aggregated temperature mesors in CIM patients was 37.2 °C [37.1; 37.4] vs. no-CIM patients 37.4 °C [37.2; 37.7] (*p* = 0.041; Fig. 4a). In the longitudinal analysis across ICU time periods, there was no significant difference in mesor between CIM and no-CIM patients (group effect,* p* = 0.700), no significant change over time (time effect, *p* = 0.181), and no significant interaction between group and time (interaction effect *p* = 0.383) (Fig. 4b, c). The linear regression model for temperature rhythm mesor adjusted for CIM, age, sex and fever (Table S1, Supplementary Material 1) explained 48% of the variance in mesor values (adjusted R^2^ = 0.47). In the model, CIM had the strongest negative (β = –0.10, 95% CI [–0.15; –0.05], *p* < 0.01) and fever (β = 0.92, 95% CI [0.87; 0.96], *p* < 0.01) the strongest positive effects on mesor values. Age and sex had no significant effects on mesor in the model.

### Temperature rhythm amplitude was lower in both CIM and no-CIM patients than in healthy bed rest volunteers, but the amplitude reduction over time was less marked in CIM compared to no-CIM groups.

**Fig. 5:**
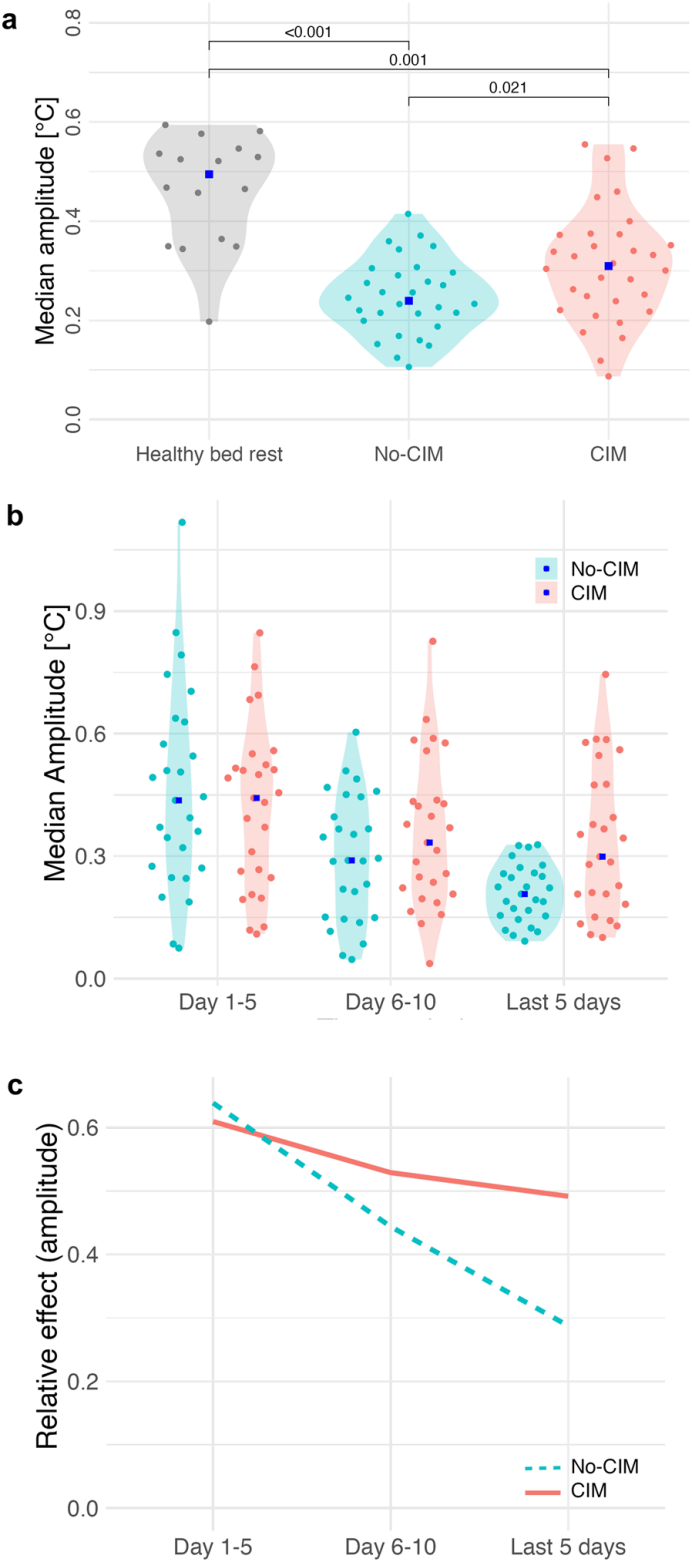
Temperature rhythm amplitude. **a** Aggregated median amplitude during ICU stay: healthy bed rest 0.5 °C [0.2; 0.6]; CIM 0.3 °C [0.2; 0.4]; no-CIM 0.2 °C [0.2; 0.3]; (*p* = 0.021).** b** Longitudinal trend of amplitude at ICU day 5, ICU day 10, and the last 5 ICU days with available data: CIM vs. no-CIM (group effect:* p* = 0.124); both groups significantly reduced the amplitude during ICU stay (time effect:* p* < 0.01).** c** Relative effects emphasized a more pronounced amplitude reduction in no-CIM vs. CIM patients over time (interaction effect:* p* = 0.017). Brunner–Munzel analysis. CIM Critical Illness Myopathy

In the healthy bed rest group, the temperature rhythm amplitude was 0.5 °C [0.2; 0.6], whereas it was lower for both CIM and no-CIM patients when amplitude was aggregated for all ICU days (Fig. 5a). CIM patients showed higher temperature rhythm amplitudes compared to no-CIM patients in the aggregated analysis (*p* = 0.021, Fig. 5a). The longitudinal analysis across ICU stay showed that temperature rhythm amplitudes decreased in the no-CIM and CIM groups (time effect: *p* < 0.01, Fig. 5b, c). The interaction between group and time showed a less pronounced reduction in temperature rhythm amplitude over time in CIM patients compared to no-CIM patients (*p* < 0.017; Fig. 5b, c).

Interindividual variability of temperature rhythm amplitude was higher in CIM compared to no-CIM patients during the last ICU days (*p* = 0.002), indicating greater heterogeneity in diurnal temperature rhythm amplitude. Variability analyses for additional rhythm parameters across ICU time periods are provided in Table S3, Supplementary Material 1.

The linear regression model for temperature rhythm amplitude adjusted for CIM, age, sex and fever is shown in Table S2, Supplementary Material 1. CIM (β = 0.07, 95% CI [0.04; 0.10], *p* < 0.01) and fever (β = 0.14, 95% CI [0.11; 0.16], *p* < 0.01) were significantly associated with higher temperature rhythm amplitude, while age (β = -0.001, 95% CI [−0.002; 0.00], *p* = 0.02) was associated with a significantly lower temperature rhythm amplitude in the model. Sex had no effect on temperature rhythm amplitude in this model. The model explained 8% of the variance in temperature rhythm amplitude (adjusted R^2^ = 0.08).

### A higher temperature rhythm amplitude correlated with MCSA of *M. vastus lateralis* myofibers in critical illness.

**Fig. 6:**
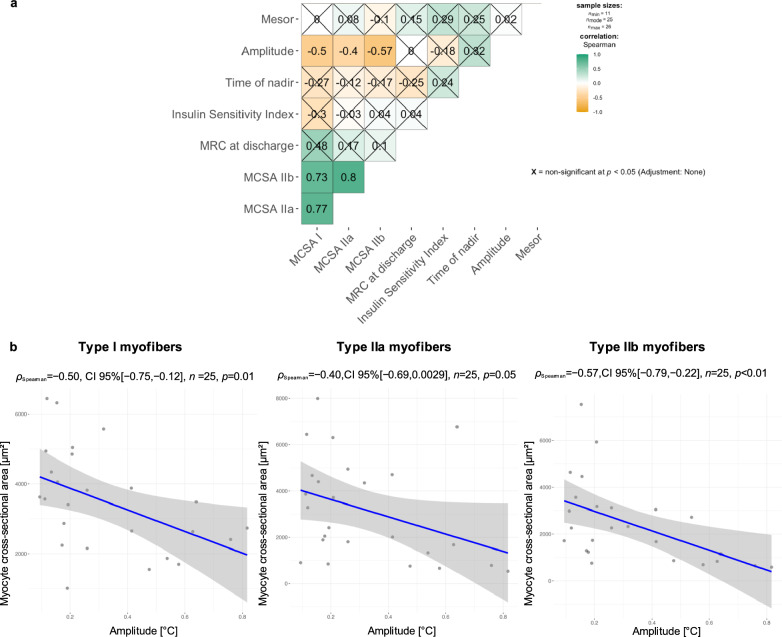
Spearman correlations. **a** Temperature rhythm parameters on the day before ICU discharge and MCSA, clinical muscle strength and insulin sensitivity. Temperature rhythm amplitude correlated with muscle atrophy metrics, but not with MRC or insulin sensitivity. **b** Temperature rhythm amplitude on the day before ICU discharge correlated negatively with myocyte cross-sectional area of myofibers type 1, IIa and IIb. Data were presented at ESICM Lives 2024 [54], Medical Research Council (MRC) Score, myocyte cross-sectional area (MCSA), Critical Illness Myopathy (CIM)

In critically ill patients who received skeletal muscle biopsy, temperature rhythm amplitude correlated negatively with MCSA of type I, IIa and IIb myofibers, but not with clinical muscle strength or metabolic study data on the day before ICU discharge (Fig. 6a, b).

### 24-h temperature rhythm periods were observable and became more pronounced towards ICU discharge in CIM and no-CIM patients.

**Fig. 7:**
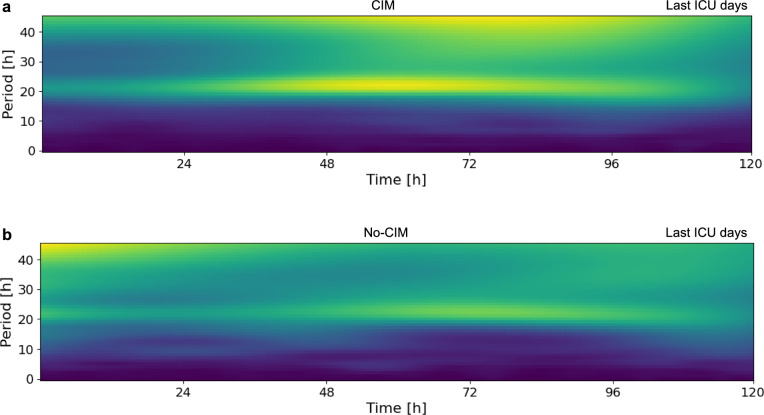
Wavelet spectogram. Bright yellow regions indicate average relative power at each time point for the temperature rhythm period. A ~ 24-h oscillatory component and a low-frequency drift can be observed in the spectrograms in **a **CIM and **b** no-CIM patients. Towards ICU discharge, a strengthening of the 24-h period is observed in the wavelet power spectrum in both groups. Visual group-level comparison does not suggest a relevant difference between groups

Continuous wavelet transformation revealed that 24-h temperature rhythm components were detectable in both CIM and no-CIM patients (Fig. 7). Temperature rhythm period varied as a function of time in the ICU in both, the CIM and no-CIM groups. Early during ICU stay, the periodic signal appeared more diffuse (Fig.S2, Supplementary Material 1), while over time, a strengthening of the 24-h period was observed in the wavelet power spectrum in both groups (Fig. 7). Most importantly, visual group-level comparison of the wavelet-derived amplitude over time revealed the same pattern as observed in the cosinor results: patients with CIM showed higher amplitudes than no-CIM patients during ICU stay (Fig.S3, Supplementary Material 1).

## Discussion

The diurnal body temperature rhythm is an evolutionarily conserved output of circadian regulation [7]. The temperature rhythm amplitude is precisely regulated by minor temporal imbalances between metabolic heat generation and thermoregulatory heat loss [7]. This requires the interplay between central circadian pacemaker and thermoregulatory peripheral tissues and organs, such as skeletal muscle for metabolic heat generation, or the skin for heat loss [7, 24, 25].

In this study, we identified phase shifts, lower amplitudes, and higher mesors of diurnal temperature rhythms in both CIM and no-CIM patients compared to healthy bed rest volunteers. While temperature rhythm amplitude declined during ICU stay in both groups of critically ill patients, the amplitude reduction was less pronounced in CIM patients, resulting in relatively higher temperature rhythm amplitudes than in no-CIM patients. This was accompanied by relatively lower temperature rhythm mesor values in the CIM compared to the no-CIM group. In both CIM and no-CIM groups, 24-h rhythm periods were detectable and became more pronounced towards ICU discharge.

### Heterogeneous phase shifts in diurnal temperature rhythms in the CIM and no-CIM groups, compared to healthy controls, may reflect conflicting ICU time cues.

 Consistent with our findings, phase-shifted body temperature rhythms have previously been reported in critically ill patients [26–28]. This may reflect disruption of circadian and thermoregulatory control through combined effects of inflammation, medication, and loss of external time cues (e.g., room lighting, nutrition timing, mobilization). Similarly, circadian disruption during critical illness has been demonstrated at the molecular level in immune cells [2]. 

Given the systemic nature of circadian regulation, the disrupted temperature rhythm may also reflect circadian dysregulation within skeletal muscle. However, direct correlation between temperature rhythm disruption and muscular clock gene dysregulation in skeletal muscle tissue would require 24-h time series biopsies, which are not feasible in critically ill patients. 

Importantly, we identified a distinct temperature rhythm pattern associated with CIM. Temperature rhythm amplitude declined during ICU stay in CIM and no-CIM groups, but relatively less amplitude suppression was observed in CIM. 

### Suppression of temperature rhythm amplitude during ICU stay could represent an adaptive response to ICU-related circadian disruption.

 Interestingly, no-CIM patients showed a more pronounced reduction in temperature rhythm amplitude during ICU stay, possibly reflecting an adaptive response to immobilization and conflicting time cues in the ICU. Temperature rhythm amplitude reductions have been similarly observed in healthy individuals during immobilization [29], induced phase shifts [30], and shift work [31]. Thus, no-CIM patients appeared to regulate temperature rhythms comparably to healthy individuals, whereas CIM patients did not. Blunted amplitude suppression observed in CIM compared to no-CIM patients may indicate reduced adaptation, potentially linked to skeletal muscle catabolism.

### Relatively higher temperature rhythm amplitude in critically ill patients was linked to CIM and skeletal muscle atrophy.

Higher temperature rhythm amplitudes in critical illness, as observed in CIM compared to no-CIM patients, have been suggested as a marker of excessive circadian activation [25]. In previous studies, higher temperature rhythm amplitudes were associated with worse survival in critical illness, particularly sepsis [26, 32]. Body temperature, metabolism and inflammation are all circadian regulated and interact closely [7, 25]. In healthy individuals, temperature rhythm amplitude correlated with robust metabolite rhythms in plasma [33]. We hypothesize that in critical illness with circadian disruption, the relatively higher temperature rhythm amplitude observed in CIM compared to no-CIM patients reflects maladaptive circadian and metabolic activation. We propose that temperature rhythms with phase-shifts and blunted amplitude suppression in CIM compared to no-CIM patients may indicate maladaptive diurnal temperature regulation associated with skeletal muscle catabolism. Whether this maladaptation causally contributes to muscle catabolism remains to be clarified*.*

Mice with skeletal muscle *Bmal1* overexpression developed insulin resistance under sleep deprivation, indicating impaired metabolic adaptability to homeostatic stress [34]. In CIM patients, we also observed insulin resistance [17], possibly related to common ICU associated sleep disruption. Moreover, in rats with systemic inflammation, external circadian disruption through constant light exposure was associated with increased muscle atrophy compared to a standard 12-h light–12-h dark cycle [35]. 

In this analysis, a higher body temperature rhythm amplitude correlated also with a smaller MCSA in *M. vastus lateralis* myofibers, further supporting our result on a histological level. The negative correlation between temperature rhythm amplitude and MCSA was particularly pronounced in type IIb myofibers. These fibers are known to be preferentially affected by circadian disruption in mice [12]. In CIM, type II myofibers are also primarily affected [36]. Type IIb myofibers more likely rely on glycolytic metabolism and are vulnerable to metabolic stress, as well as to circadian disruption [12, 18, 36]. In chronic inflammatory atrophic conditions, circadian disruption and a more pronounced type IIb myofiber atrophy have been reported [37–39]. The link between temperature rhythm and skeletal muscle clock rhythm disruption remains hypothetical, as time-series biopsies were not performed. This is currently being explored in a complementary molecular animal study.

## Confounders and limitations

Body temperature rhythms reflect diurnal regulation. However, body temperature and its rhythm in the ICU are influenced by various factors, such as age, sex, inflammation, organ support systems, artificial temperature control, medications, nutrition, or fluid status. Moreover, the observed higher interindividual variability in temperature rhythm amplitude observed in CIM patients during late ICU stay may reflect diverging recovery trajectories.

A potential concern is that fitting a fixed 24-h period Cosinor model to a non-24-h rhythm may introduce bias. However, our wavelet analysis demonstrated the presence of underlying ~ 24-h oscillatory components in both CIM and no-CIM groups, which became more pronounced towards ICU discharge, the time point at which group differences in temperature rhythm amplitude were particularly relevant. In addition, wavelet-derived amplitude patterns over the ICU stay closely mirrored those identified by Cosinor analysis. The consistency between both methods supports the use of fixed 24-h Cosinor modeling in this setting.

Higher age was associated with CIM and with smaller MCSA of type I, IIa, and IIb myofibers. Since CIM patients were older, age was considered a potential confounder of the observed association between CIM and higher temperature rhythm amplitude. However, in our multivariable linear regression model adjusting for CIM, age, sex, and fever, age was independently associated with a lower temperature rhythm amplitude, while CIM remained significantly associated with a higher temperature rhythm amplitude. This indicates that age does not explain the increased temperature rhythm amplitude in CIM. Temperature rhythm amplitude reduction in ageing has been described before [40].

As expected, fever was associated with a higher temperature rhythm mesor and amplitude. However, CIM remained independently associated with a lower temperature rhythm mesor and a higher amplitude in the adjusted model. This suggests that temperature rhythm alterations in CIM cannot be explained by fever alone. The model explained only a small proportion of the variance in temperature rhythm amplitude, indicating that additional factors may contribute. High temperature rhythm amplitudes [41] and greater temperature changes in a 24-h period were associated with sepsis development even in afebrile ICU patients [42]. Therefore, our findings may reflect more severe sepsis in CIM patients. However, both groups were severely ill and the sepsis severity based on the SOFA score was similar between CIM and no-CIM groups. Still, systemic inflammation likely plays a key role for circadian rhythm disruption and for skeletal muscle wasting in critical illness [39]. 

The data are limited by the small sample size, which did not allow for adjustments of all confounders. The retrospective study design and the ICU setting limit the generalizability of this hypothesis-generating analysis. The bed rest group is a reference for physiological temperature rhythms under immobilization [20] but does not replicate the conditions of critical illness.

## Conclusions

Heterogeneous phase shifts of diurnal temperature rhythms in CIM and no-CIM groups compared to healthy bed rest volunteers may indicate ICU-related circadian disruption. Suppression of temperature rhythm amplitude during ICU stay could represent an adaptive response to this disruption. Blunted amplitude suppression observed in CIM compared to no-CIM patients might reflect reduced adaptation, potentially linked to muscle catabolism. This hypothesis-generating analysis underlines the need for mechanistic studies exploring circadian regulation in skeletal muscle during critical illness.

## Methods

### Aims

Aim of this analysis was to compare diurnal body temperature rhythms between CIM and no-CIM patients from two prospective ICU trials [18, 19] in reference to *n* = 16 participants included in a bed rest study [20]. Critically ill patients were classified CIM (*n*= 32) or no-CIM (*n* = 30) based on electrophysiological tests in the prior studies [18, 19]. Further aims were to test correlations between temperature rhythm parameters and baseline characteristics, histological muscle atrophy metrics in the form of MCSA values of type I, IIa, and IIb myofibers from *M. vastus lateralis* biopsies, clinical muscle strength quantified by the Medical Research Council (MRC) strength scale, and metabolic insulin sensitivity index determined by hyperinsulinemic-euglycemic clamp. The detailed study protocols are described in previous publications [18, 19, 21, 36].

### Study design and setting

This is a secondary data analysis of two prospective clinical trials (ISRCTN77569430 and ISRCTN19392591) which focused on CIM and skeletal muscle metabolism and mobilization in critical illness [18, 19]. The trials included mechanically ventilated adult patients with a Sequential Organ Failure Assessment (SOFA) Score of ≥ 8 [18, 19]. Patients or legal proxies provided written informed consent prior to their inclusion to the study. The Charité Ethics Committee approved the clinical trials (EA2/061/06, EA2/041/10) and this secondary data analysis (EA1/284/22 January 2023, amended 01 August 2024). The trials were conducted at two tertiary care ICUs at Charité–Universitätsmedizin Berlin.

External time cues were not aligned with physiological rhythms in the ICU setting. If artificial nutrition was indicated, enteral 24-h continuous nutrition was the clinical standard during the study period. Patients were treated in standard ICU rooms, featuring northeast- or southeast-facing windows of approximately 11 m^2^ [43].

Conventional lighting in our ICU rooms achieved a circadian effective irradiance (a measure based on the action spectrum for melatonin suppression) between 0.29 and 0.5 W/m^2^, as indicated by spectroradiometric measurements [43–45]. While low illuminance levels influence melatonin secretion in healthy individuals under controlled conditions [44, 45], thresholds in critical illness remain unclear. ICU patients frequently show altered melatonin physiology and disrupted rhythms [46, 47]. Even exposure to complete darkness (< 1 lx) or bright light (> 10,000 lx) did not result in a consistent melatonin response in critically ill patients [47].

Mobilization methods evolved over time, ranging from standard physiotherapy to protocol-based physiotherapy, and to protocol-based plus advanced muscle activation measures, as outlined in the study protocols [17, 19, 36]. Physiotherapeutic measures were typically performed during the early shift in the ICU, specific time points were not documented. Still, patients were mainly immobilized.

We refer to temperature rhythms as *diurnal* rather than *circadian*, since temperature measurements were performed under standard ICU conditions without experimental control of masking factors such as light, nutrition, or mobility.

### Groups

Critically ill patients who received electrophysiological CIM diagnostics were selected from both prospective clinical trials and were grouped based on whether they developed CIM or not. CIM was diagnosed through direct muscle stimulation compound muscle action potential (dmCMAP) in both trials [18]. The CIM group was characterized by the dmCMAP < 3 mV, while patients with dmCMAP > 3 mV were classified as no-CIM [48]. To complement the electrophysiological diagnosis of CIM, additional metrics were reported for the CIM and no-CIM groups. These metrics included histological muscle atrophy measurements, represented by MCSA values of type I, IIa, and IIb myofibers from *M. vastus lateralis* biopsies; clinical muscle strength, assessed using the Medical Research Council (MRC) strength scale; and metabolic insulin sensitivity index, determined via a hyperinsulinemic-euglycemic clamp.

As a reference, we used publicly available temperature data from male volunteers (mean age 31 years) that participated in a study that investigated the effects of head-down tilt (− 6°) bed rest, a commonly used space-flight analogue, with and without exercise on diurnal body temperature rhythms [20].

### Diurnal temperature rhythm analysis

In ICU patient care, temperature was continuously measured using a thermometer attached to the urinary bladder catheter. Measurements were stored electronically every 30 min and data from the whole ICU stay were considered for analysis. Urinary bladder temperature measurements have a high accuracy in critically ill patients, when compared to the gold standard of pulmonary artery temperature measurements and are less invasive [49]. For the healthy bed rest group, continuously reported 24-h rectal temperature after one week of bed rest were used. Temperature data of ICU and healthy bed rest groups were preprocessed (Tables S4 and S6, Supplementary Material 1). An overview of excluded temperature values is provided in Table S4, Supplementary Material 1.

**Fig. 8:**
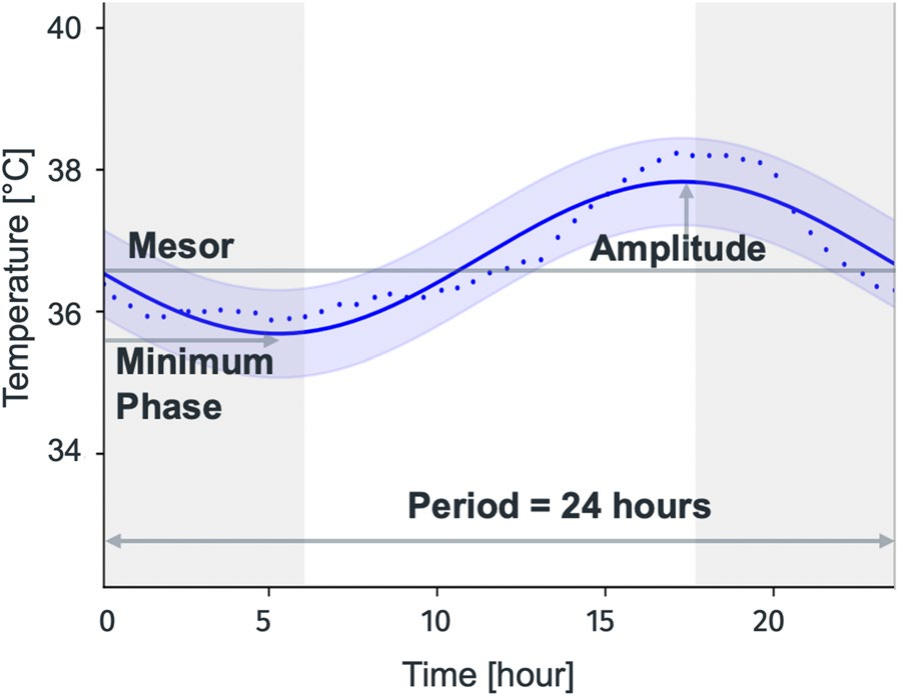
Cosinor analysis and rhythm parameters of an ICU patient with physiological temperature rhythm. The blue dots represent the measured body temperature, the blue line indicates the fitted cosinor model with confidence intervals. The nadir is observed in the early morning hours, while the peak is observed in the late afternoon

Cosinor analysis was performed based on the pre-processed temperature data. Therefore, single-component cosinor models were fitted per patient and day using the *CosinorPy* library (v2.1) [50]. We used a 1-component Cosinor model as the primary approach due to its clinical interpretability. For transparency, a 2-component Cosinor analysis was additionally performed. Details are provided in Table S5, Supplementary Material 1. The period was set to 24 h. The temperature rhythm parameters (endpoints) time-of-day of temperature nadir (reflecting the minimum phase), amplitude and mesor were the outputs of the cosinor analysis, and the R^2^ indicated the goodness of fit (Fig. 8). The temperature nadir time (minimum phase) was estimated for all fits, including those with suppressed amplitudes.

Rhythm classification was determined for ICU day 5, ICU day 10, and the day before ICU discharge using the *compareRhythms* R library [51], which took temperature as an input and performed a cosinor-based classification in comparison to the healthy bed rest group. CompareRhythms by default classified the diurnal temperature rhythms of CIM or no-CIM patients into three categories for each investigated ICU day relative to healthy bed rest volunteers: ‘lower amplitude’ (CompareRhythms category: loss), ‘phase-shifted’ (CompareRhythms category: change), and ‘comparable rhythm’ (CompareRhythms category: same) [51]. Rhythms were considered present when the adjusted *p*-value of the Cosinor fit was < 0.05 and the amplitude exceeded a predefined threshold. To define this amplitude cut-off, we calculated the average max–min difference in the raw temperature profiles of healthy bed rest individuals, which was 1.10 °C. Half of this value, 0.55 °C, was used as the biological amplitude cut-off (the default cut-off in this context).

Phase-shifted temperature rhythm compared with healthy bed rest individuals (CompareRhythms category: change): The modeled diurnal temperature rhythm of the investigated CIM or no-CIM patient showed a *p*-value < 0.05 and an amplitude > 0.55 °C, but differed in one or more Cosinor rhythm parameters compared to the healthy bed rest group.

Lower temperature rhythm amplitude compared with healthy bed rest individuals (CompareRhythms category: loss): The modeled diurnal temperature rhythm of the investigated CIM or no-CIM patient had either amplitude ≤ 0.55 °C and/or an adjusted *p*-value ≥ 0.05.

Comparable temperature rhythm in comparison with healthy bed rest individuals (CompareRhythms category: same): The fitted temperature rhythm had a *p*-value < 0.05 and an amplitude > 0.55 °C in the investigated CIM or no-CIM patient, and the temperature rhythm parameters did not differ significantly from the healthy bed rest group.

The temperature rhythm constitutes complex waveforms with multiple oscillatory components. To further support interpretation of fixed 24-h period Cosinor modeling, we assessed the presence of underlying ~ 24-h oscillatory components using wavelet analysis. We provided average spectrograms for ICU days 1–5, 5–10, and last ICU days for the CIM and no-CIM groups. We performed time–frequency analysis using continuous wavelet transforms implemented in the pyBOAT package [52], employing the complex Morlet wavelet due to its good time and frequency localization properties. For each time series, we computed the wavelet power spectrum (spectrogram), capturing the temporal evolution of power across a range of frequencies. To facilitate meaningful averaging across multiple recordings, each individual spectrogram was first normalized by its maximum value. The resulting normalized spectrograms were then averaged across all samples. Finally, the averaged spectrogram was globally normalized so that the total sum of all power values equaled 1, enabling visual group-level comparisons of relative power distributions across time and frequency. Bright yellow regions indicated periods at a given point in time with a high relative power.

### Statistical analysis

The descriptive analysis results were expressed as median and 25th and 75th percentiles. Group comparisons of temperature rhythm parameters and assigned rhythm classes were based on aggregations for the entire ICU stay or longitudinally, or for specific single days of interest. Non-parametric Brunner*–*Munzel tests and longitudinal procedures, *nparcomp* package [53], and Chi-Square tests were used. The Brown–Forsythe test was used to compare interindividual variability of temperature rhythm parameters between the CIM and no-CIM groups. Temperature rhythm parameters were correlated with clinical, histological, and metabolic study data by Spearman correlation.

To account for potential confounding, we performed a linear regression analysis for each rhythm parameter and adjusted for CIM, age, gender, and fever. A statistician (KR) was involved in the formal analyses. Data preprocessing and analyses have been implemented using RStudio, R, Python and SciPy, versions, packages and references are listed in Table S6, Supplementary Material 1.

## Supplementary Information

Supplementary material 1.

Supplementary material 2.

## Data Availability

The analyzed anonymous datasets are available from the corresponding author or dai-researchdata@charite.de on reasonable request. Preliminary results were presented in abstract form at *ESICM Lives 2024* and published [54].

## Abbreviations

APACHE: Acute physiology and chronic health evaluation
*Bmal1*: Basic helix-loop-helix ARNT like 1
BMI: Body mass index
CIM: Critical illness myopathy
DMCMAP: Direct muscle stimulation compound muscle action potential
ICU: Intensive care unit
MCSA: Myocyte cross-sectional area
MRC: Medical research council
R^2^: Coefficient of determination
SAPS2: Simplified acute physiology score II
SOFA: Sequential organ failure assessment
