# Supplementary material for: Distinct diurnal temperature rhythm patterns in critical illness myopathy: secondary analysis of two prospective trials
Source: Ann Intensive Care. 2025 Oct 27;15:171. doi: 10.1186/s13613-025-01582-5 (PMC12554853; doi:10.1186/s13613-025-01582-5)
Supplement: Supplementary file 1 — Supplementary material 1. [file 13613_2025_1582_MOESM1_ESM.pdf]

Distinct circadian temperature rhythm patterns in critical illness myopathy: secondary analysis of two prospective trials

D Mewes[1,2]; S Weber-Carstens[1]; K Rubarth[3]; SD Boie[4]; C Spies[1]; A Kramer[6]; J Fielitz [6,7]; T Wollersheim[1]; B Ananthasubramaniam[8]; F Braune[1]; L Hancke[1];
L Spies[4]; F Balzer[4]; LJ Engelhardt[1,9#]

Additional File 1

**Additional Figures**
**Fig. S1** Body temperature rhythms ICU Day 5 and ICU day 10
**Fig. S2** Wavelet analysis ICU days 1-5 and ICU days 5-10
**Fig. S3** Comparison of temperature rhythm amplitude in CIM and no-CIM groups using different methods. (**a**) Cosinor analysis (**b**) Wavelet analysis.
**Additional Tables**
**Table S1** Multivariable linear regression model for temperature rhythm mesor
**Table S2** Multivariable linear regression model for temperature rhythm amplitude
**Table S3** Temperature rhythm parameter variability
**Table S4** Excluded temperature values
**Table S5** Temperature rhythm parameters based on two-component Cosinor analysis
**Table S6** Body temperature data preprocessing and software packages
**Additional References**

Additional Figures

a

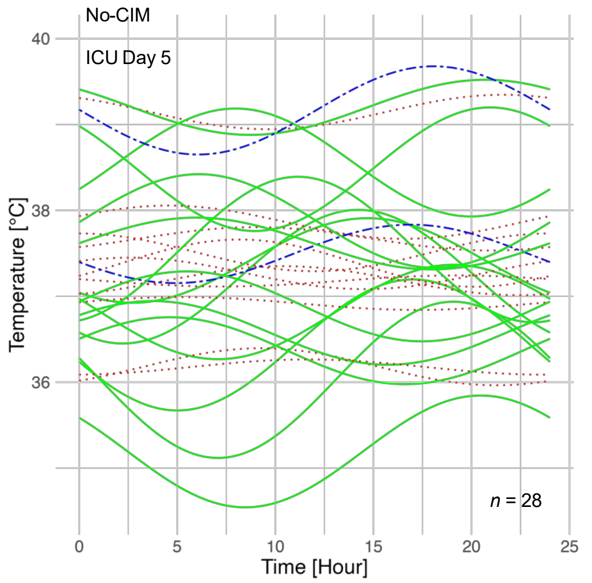

b

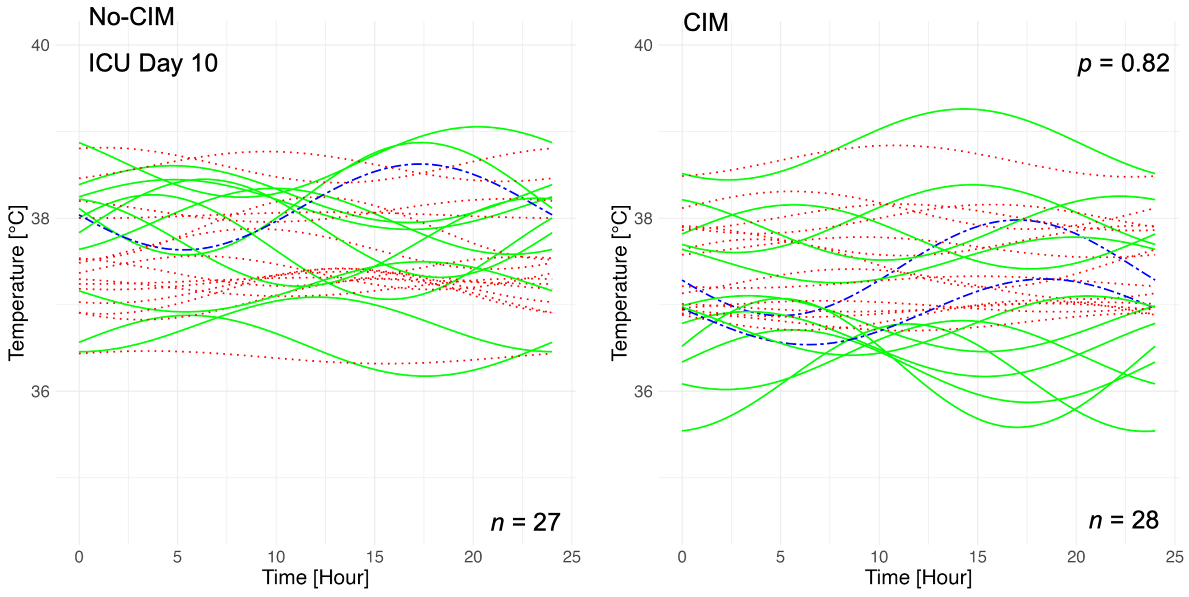

Fig. S1 Body temperature rhythms (**a**) ICU day 5 and (**b**) ICU day 10**.** Cosinor analysis in no-CIM and CIM patients, classified relative to the healthy bed rest group by *CompareRhythms*: orange *loss* (amplitude loss), green *change* (phase shift), blue *same* amplitude and phase. In the initial phase of critical illness, both groups showed temperature rhythms with phase-shifts and amplitude loss. Rhythm class distribution in no-CIM vs. CIM groups on ICU day 5 and ICU day 10, Chi-square test. Data were presented at *ESICM Lives 2024*, the Abstract has been published (1).

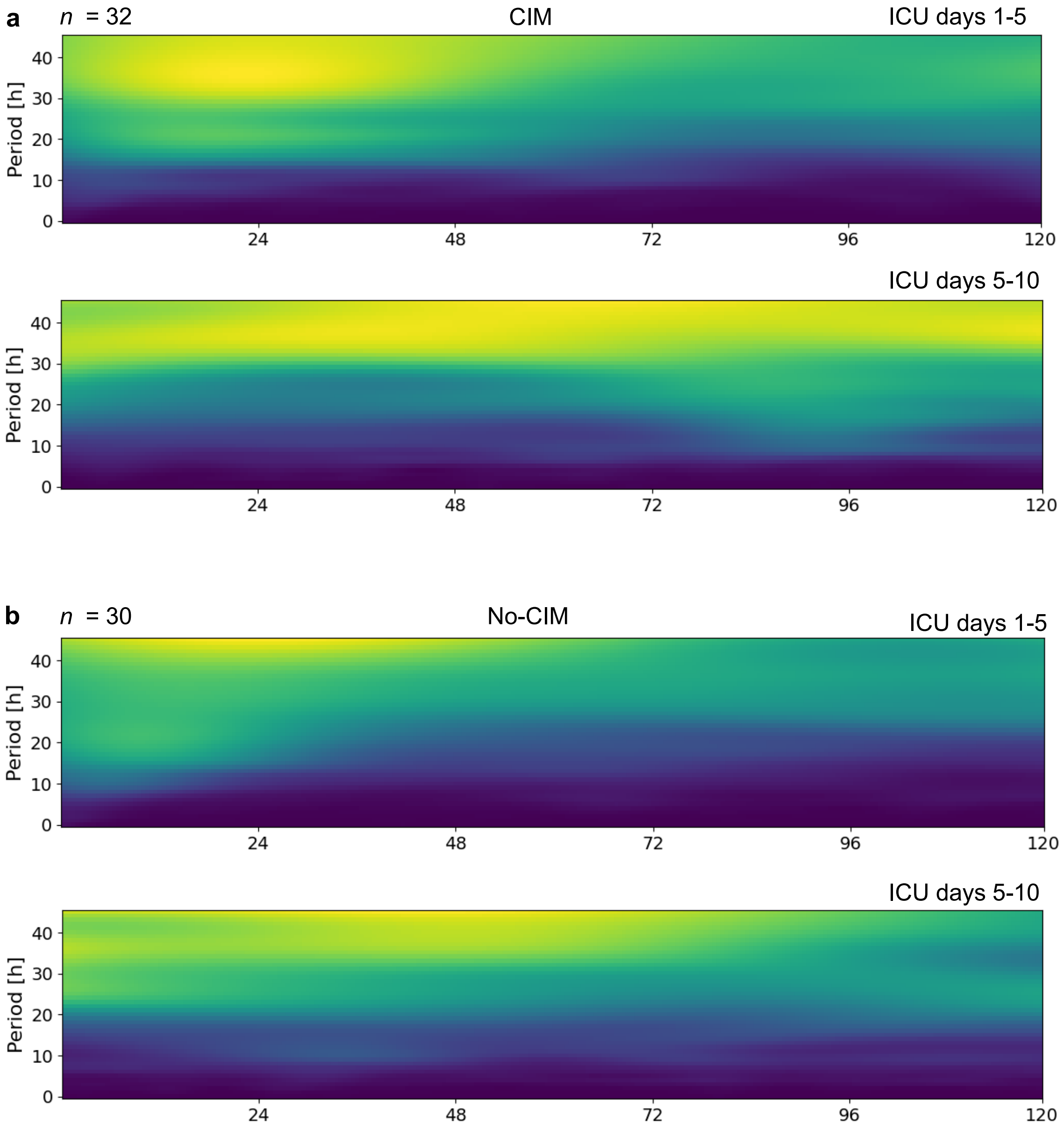

Fig. S2 Wavelet spectrogram ICU days 1-5 and ICU days 5-10 Bright yellow regions indicate average temperature rhythm periods at the given time point during ICU stay with a high relative power. Temperature rhythm period varies as a function of time in the ICU in both, the CIM and no-CIM groups. Early during ICU stay, the periodic signal appears more diffuse, while over time, a strengthening of the 24-hour period is observed in the wavelet power spectrum in both groups (Fig.7).Visual group-level comparison does not suggest a relevant difference between groups.

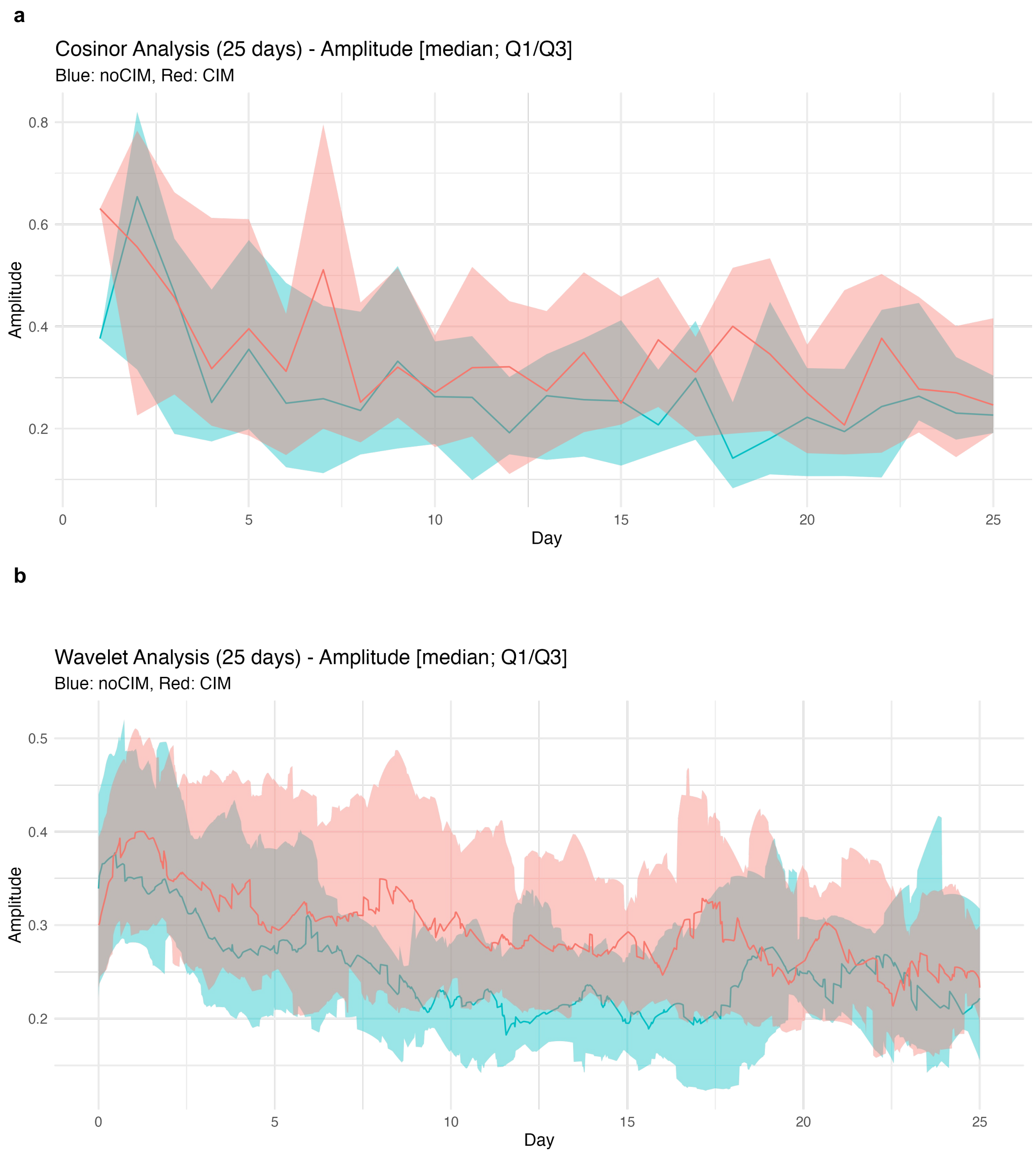

Fig. S3 Comparison of temperature rhythm amplitude in CIM and no-CIM groups using different analysis methods. (a) **Cosinor analysis**: Temperature rhythm amplitude is consistently higher in CIM compared to no-CIM patients on most ICU days between day 1 and 25. (b) **Wavelet analysis**: Temperature rhythm amplitude is likewise consistently higher in CIM compared to no-CIM patients across the majority of ICU days between day 1 and 25.

Table S1 Multivariable linear regression model for temperature rhythm mesor

n=62 patients (no-CIM *n=30,* CIM *n=32*)

**Response Variable**	**Variable**	**β**	**95% CI of ß**	**ß** standardized	***p***	**R^2**	**R^2** adjusted	***p*** model
Mesor	Intercept	36.95	[36.86;37.03]		<0.01	0.48	0.47	<0.01
	CIM	-0.10	[-0.15; -0.05]	-0.08	<0.01			
	Age	0.00	[0.00/0.00]	0.01	0.71			
	Sex	0.05	[0.00/0.10]	0.04	0.06			
	Fever	0.92	[0.87/0.96]	0.68	<0.01			

B regression coefficient, ß standardized regression coefficient, CI Confidence interval, CIM Critical illness myopathy

Table S2. Multivariable linear regression model for temperature rhythm amplitude

n=62 patients (no-CIM *n*=30*,* CIM *n*=32)

**Response Variable**	**Variable**	**β**	**95% CI of ß**	**ß** standardized	***p***	**R^2**	**R^2** adjusted	***p*** model
Amplitude	Intercept	0.29	[0.25;0.34]		<0.01	0.09	0.08	<0.01
	CIM	0.07	[0.04;0.1]	0.13	<0.01			
	Age	-0.001	[-0.002/0.00]	-0.06	0.02			
	Sex	-0.02	[-0.05; 0.01]	-0.04	0.11			
	Fever	0.14	[0.11;0.16]	0.26	<0.01			

B regression coefficient, ß standardized regression coefficient, CI Confidence interval, CIM Critical illness myopathy

Table S3 Interindividual temperature rhythm variability

Rhythm parameter	ICU Days	CIM	No-CIM	*p*-value
Amplitude variability	1-5	0.44 [0.26]	0.44 [0.33]	0.515
Mesor variability	1-5	37.25 [0.90]	37.13 [1.06]	0.735
Time of nadir variability	1-5	11.41 [5.87]	12.92 [6.14]	0.765
Amplitude variability	6-10	0.33 [0.22]	0.29 [0.27]	0.191
Mesor variability	6-10	37.29 [0.68]	37.42 [1.13]	0.617
Time of nadir variability	6-10	10.98 [5.86]	9.80 [3.64]	0.788
Amplitude variability	Last 5	0.30 [0.28]	0.21 [0.10]	0.002
Mesor variability	Last 5	37.23 [0.80]	37.26 [0.31]	0.118
Time of nadir variability	Last 5	9.56 [7.35]	8.89 [8.19]	0.902

Results in Median [IQR]; last ICU days refers to last days with available temperature data
Brown-Forsythe Test;

Table S4. Excluded temperature values basic statistics

	**CIM**	**No-CIM**
Patients (*n*)	32	30
Overall temperature values per group (*n*)	50553	41005
Excluded temperature values <34°C<br>(*n* and % of overall temperature values)	515 (1.0%)<br>mean value: 30.9 °C	270 (0.7%)<br>mean value: 30.1 °C
Excluded temperature values >41°C<br>(*n* and % of overall temperature values, mean value)	4 (0.01%)<br>mean: 41.5 °C	6 (0.01%)<br>mean: 41.4 °C
Temperature values outliers excluded (*n and %)*)	50034 (99.0%)	40729 (99.3%)
Overall ICU days per group* (*n*)	1226	938
Excluded ICU days with data gaps >4h<br>(*n* and % of overall ICU days)	325 (26.5%)	210 (22.3%)
Overall days included in Cosinor Analysis per group<br>(*n* and % of overall ICU days)	901 (73.5%)	728 (77.7%)

* measurement outlier removal reduced both CIM and No-CIM by two days, as these consisted entirely of outlying values.

Table S5. Temperature rhythm parameters based on two-component Cosinor analysis

Rhythm parameter	CIM	No-CIM	*p*-value
R^2	0.74 [0.68, 0.79]	0.71 [0.65, 0.77]	0.25
Amplitude (in °C)	0.42 [0.36, 0.48]	0.33 [0.26, 0.41]	0.02
Mesor (in °C)	37.24 [37.09, 37.4]	37.41 [37.18, 37.7]	0.07
Time of temperature nadir 1 (hh:mm)	7.21 [6.28, 8.35]	7.89 [6.91, 11.18]	0.15
Time of temperature nadir 2 (hh:mm)	17.18 [15.3, 18.59]	17 [15.84, 19.14]	0.67
Missing second component (%)	0.54 [0.47, 0.63]	0.58 [0.5, 0.65]	

Brunner Munzel Test; The two-component Cosinor analysis improved model fit (R^2), as expected. However, a second peak was not consistently detectable (58% of no-CIM and 54% of CIM patients showed no second peak). Importantly, the central finding of a higher amplitude in CIM remained unchanged in the two-harmonic Cosinor model.

Table S6. Body temperature data preprocessing and software packages

**Filtering and pre-processing**	▪ Individual measurements below 34 °C were removed since the data below the physiological range were frequently single outliers (details see Table S5)<br>▪ Days with time gaps in body temperature ≥ 4 hours were excluded (details see Table S5)<br>▪ For the healthy bed rest group, 24-hour rectal temperature data after one week of bed rest were resampled to 60-minute intervals for the complete day of measurements using the *round_date* function of the *lubridate* package for R (2).<br>▪ The ICU patients temperature measurements were resampled to 60-minute intervals before performing rhythm classification. The compareRhythms package requires regular data points. We decided to perform hourly resampling (60 minutes), as a compromise between preserving clinically meaningful temperature fluctuations and allowing the use of days with fewer measurements. This was performed using the *round_date* function of the *lubridate* package for R (2), reducing the temporal resolution by taking the mean of all measurements within each hour.<br>▪ The cut-off value used for the distinction between *loss* and *change* categories in the *compareRhythms* classification was based on the healthy bed rest

	group, as described in the Methods (main manuscript).
**Software for data importing, pre-processing and analysis**	▪ Importing, cleaning, and analysis of data have been implemented using using RStudio (version 2023.12.0+369) and R (version 4.3.2 (2023-10-31) (3), Python (v.3.11.3) and SciPy (v.1.11.4).<br>▪ The CosinorPy library (v2.1) for cosinor analysis (4), reticulate package was used to execute Python code within R markdown notebooks.<br>▪ Rhythm classification was determined for ICU day 5, ICU day 10, and the day before ICU discharge using the *compareRhythms* R library (5).<br>▪ Further R packages: broom (6), , flextable (7), ggbeeswarm(8), ggpubr(9), ggstatsplot (10), knitr (11), lubridate(12), nparcomp (13), nparLD (13), patchwork(14), reticulate (15), rmarkdown (16), table1 (17), tidyverse(18)

References

1. Engelhardt LJ, Mewes D, Spies C, Wollersheim T, Kramer A, Ananthasubramaniam B, Balzer F, Rubarth K, Boie S, Weber-Carstens S: Circadian Temperature Rhythms and the Development of Critical Illness Myopathy (Best Abstracts). *Intensive Care Medicine Experimental* 2024; 12:87

2. Mendt S, Gunga H-C, Felsenberg D, et al.: Regular exercise counteracts circadian shifts in core body temperature during long-duration bed rest [Internet]. *npj Microgravity* 2021; 7:1–6[cited 2023 Apr 21] Available from: https://www.nature.com/articles/s41526-020-00129-1

3. R Core Team: R: a language and environment for statistical computing [Internet]. Vienna, Austria: R Foundation for Statistical Computing; 2023. Available from: https://www.R-project.org/

4. Moškon M: CosinorPy: a python package for cosinor-based rhythmometry. *BMC Bioinformatics* 2020; 21:485

5. Pelikan A, Herzel H, Kramer A, et al.: Venn diagram analysis overestimates the extent of circadian rhythm reprogramming. *FEBS J* 2021;

6. Robinson D, Hayes A, Couch S: broom: Convert statistical objects into tidy tibbles [Internet]. 2023. Available from: https://CRAN.R-project.org/package=broom

7. Gohel D, Skintzos P: flextable: Functions for tabular reporting [Internet]. 2024. Available from: https://CRAN.R-project.org/package=flextable

8. Clarke E, Sherrill-Mix S, Dawson C: ggbeeswarm: Categorical scatter (violin point) plots [Internet]. 2023. Available from: https://github.com/eclarke/ggbeeswarm

9. Kassambara A: ggpubr: “ggplot2” based publication ready plots [Internet]. 2023. Available from: https://rpkgs.datanovia.com/ggpubr/

10. Patil I: Visualizations with statistical details: The “ggstatsplot” approach [Internet]. *Journal of Open Source Software* 2021; 6:3167Available from: https://doi.org/10.21105/joss.03167

11. Xie Y: knitr: a comprehensive tool for reproducible research in R. In: Stodden V, Leisch F, Peng RD, editor(s). Implementing reproducible computational research. Chapman and Hall/CRC; 2014.

12. Grolemund G, Wickham H: Dates and times made easy with lubridate [Internet]. *Journal of Statistical Software* 2011; 40:1–25Available from: https://www.jstatsoft.org/v40/i03/

13. Konietschke F, Placzek M, Schaarschmidt F, et al.: nparcomp: An R software package for nonparametric multiple comparisons and simultaneous confidence

intervals [Internet]. *Journal of Statistical Software* 2015; 64:1–17Available from: http://www.jstatsoft.org/v64/i09/

14. Pedersen TL: patchwork: The composer of plots [Internet]. 2023. Available from: https://CRAN.R-project.org/package=patchwork

15. Ushey K, Allaire J, Tang Y: reticulate: Interface to “Python” [Internet]. 2023. Available from: https://CRAN.R-project.org/package=reticulate

16. Xie Y, Dervieux C, Riederer E: R markdown cookbook [Internet]. Boca Raton, Florida: Chapman and Hall/CRC; 2020. Available from: https://bookdown.org/yihui/rmarkdown-cookbook

17. Rich B: table1: Tables of descriptive statistics in HTML [Internet]. 2023. Available from: https://CRAN.R-project.org/package=table1

18. Wickham H, Averick M, Bryan J, et al.: Welcome to the tidyverse. *Journal of Open Source Software* 2019; 4:1686
