# Supplementary material for: Distinct diurnal temperature rhythm patterns in critical illness myopathy: secondary analysis of two prospective trials
Source: Ann Intensive Care. 2025 Oct 27;15:171. doi: 10.1186/s13613-025-01582-5 (PMC12554853; doi:10.1186/s13613-025-01582-5)
Supplement: Supplementary file 2 — Supplementary material 2. [file 13613_2025_1582_MOESM2_ESM.pdf]

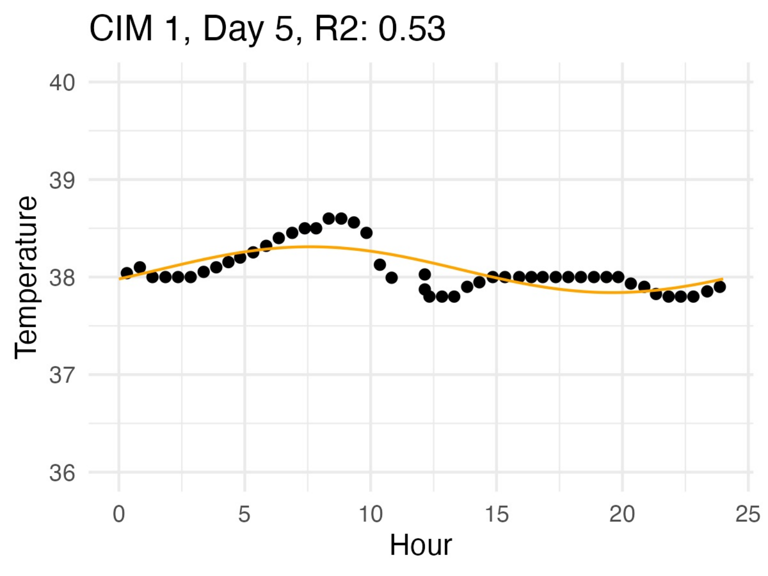
CIM 1, Day 5, R2: 0.53
Temperature
40
39
38
37
36
Hour
0
5
10
15
20
25

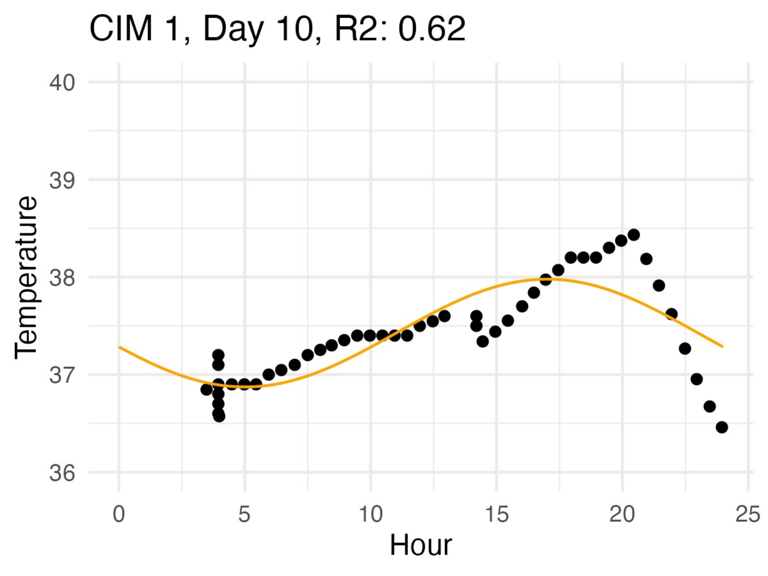
CIM 1, Day 10, R2: 0.62
Temperature
40
39
38
37
36
Hour
0
5
10
15
20
25

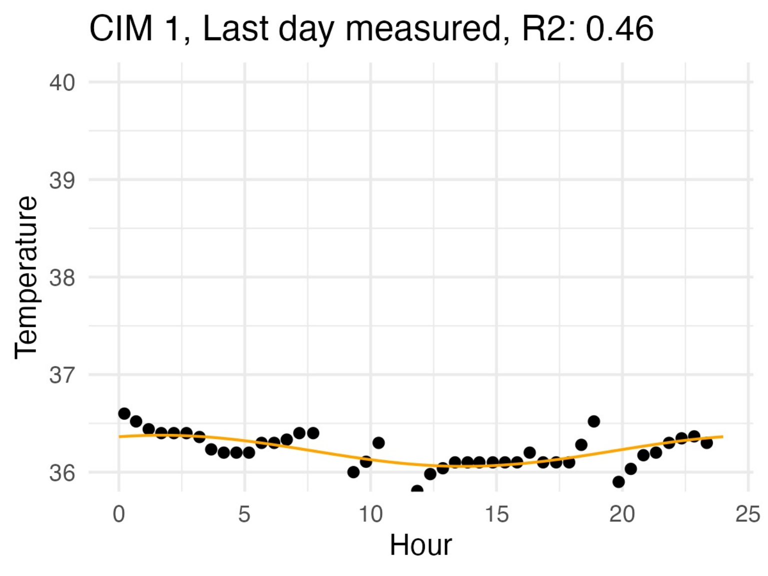

CIM 1, Last day measured, R2: 0.46
Temperature
Hour
40
39
38
37
36
0
5
10
15
20
25

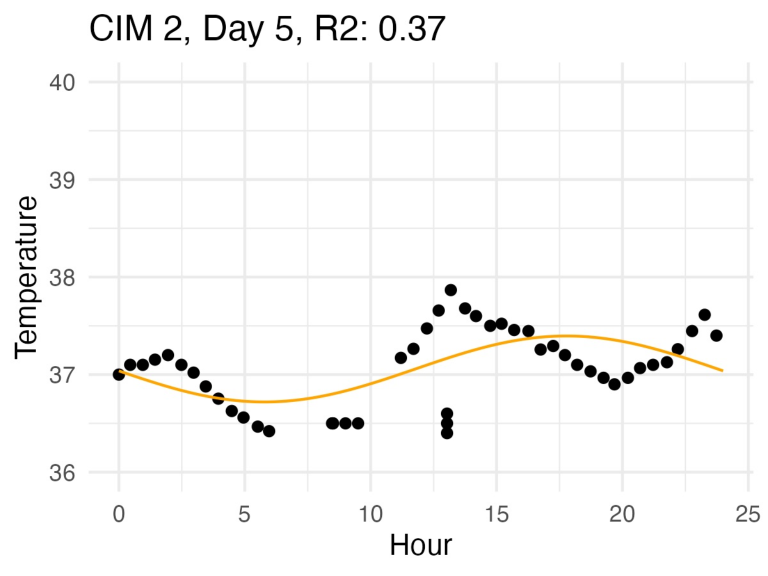
CIM 2, Day 5, R2: 0.37
Temperature
40
39
38
37
36
Hour
0
5
10
15
20
25

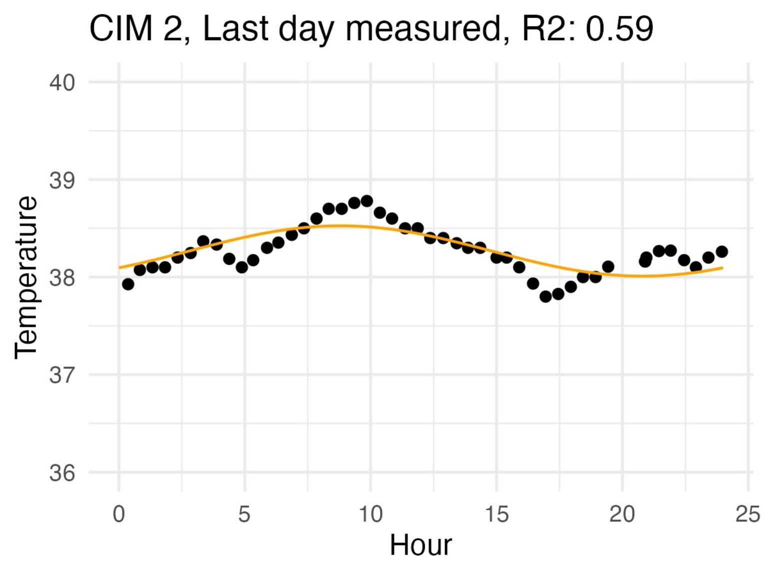
CIM 2, Last day measured, R2: 0.59
Temperature
40
39
38
37
36
Hour
0
5
10
15
20
25

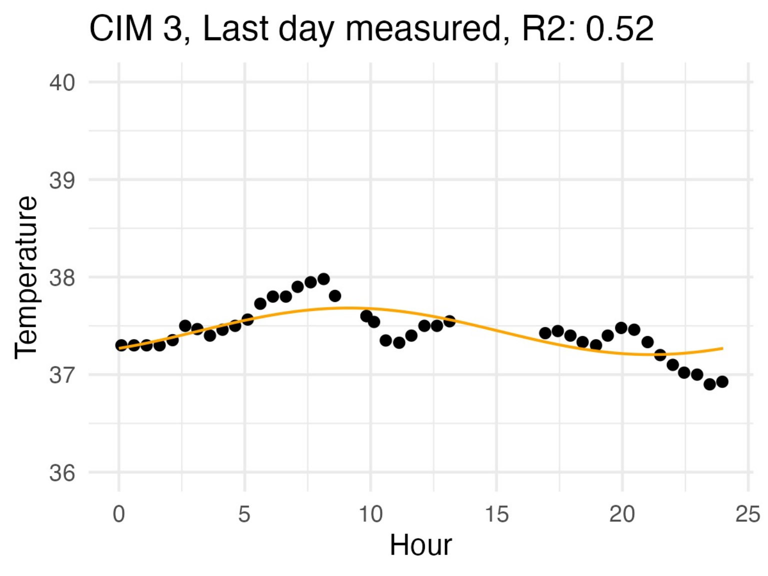

CIM 3, Last day measured, R2: 0.52
Temperature
40
39
38
37
36
0
5
10
15
20
25
Hour

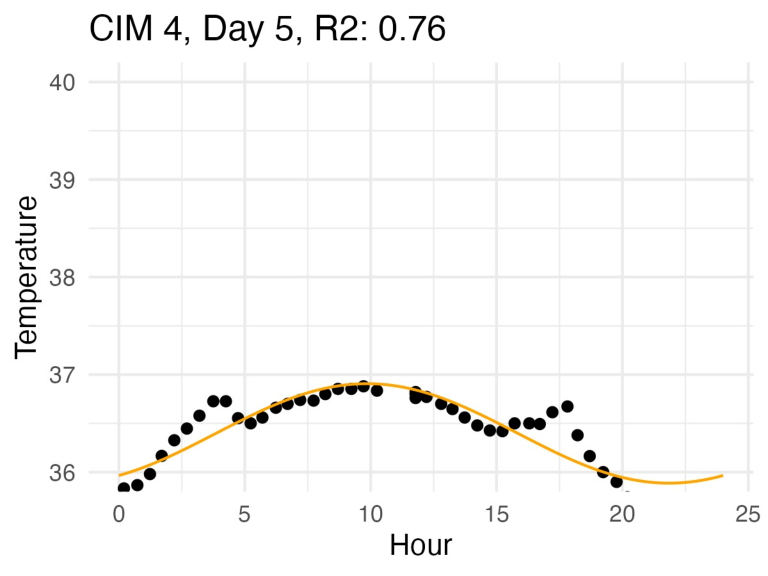

CIM 4, Day 5, R2: 0.76
Temperature
40
39
38
37
36
0
5
10
15
20
25
Hour

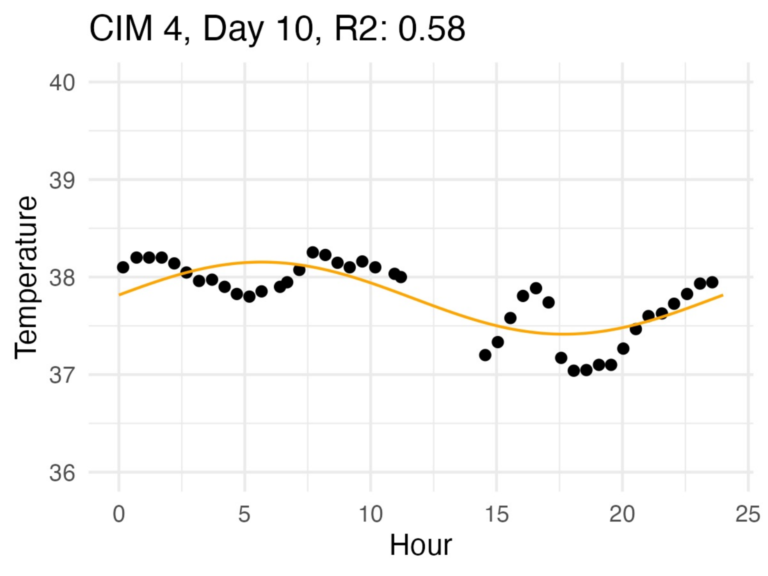
CIM 4, Day 10, R2: 0.58
Temperature
40
39
38
37
36
Hour
0
5
10
15
20
25

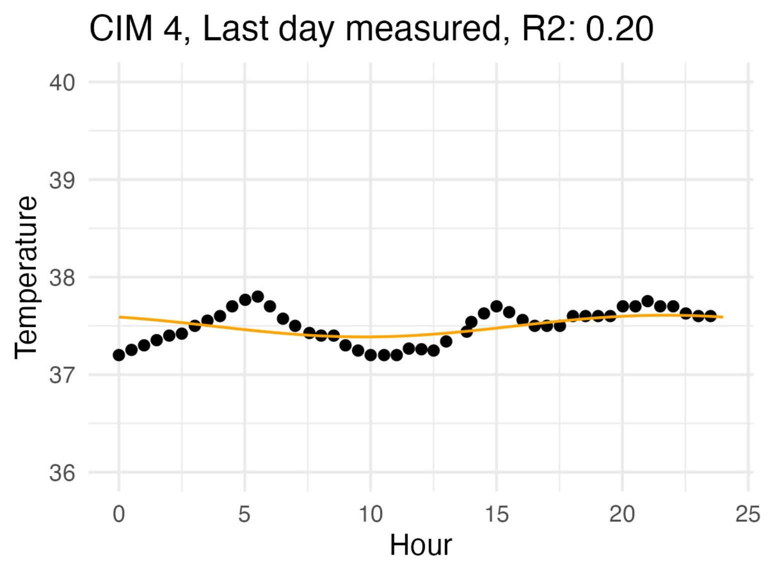
CIM 4, Last day measured, R2: 0.20
Temperature
40
39
38
37
36
Hour
0
5
10
15
20
25

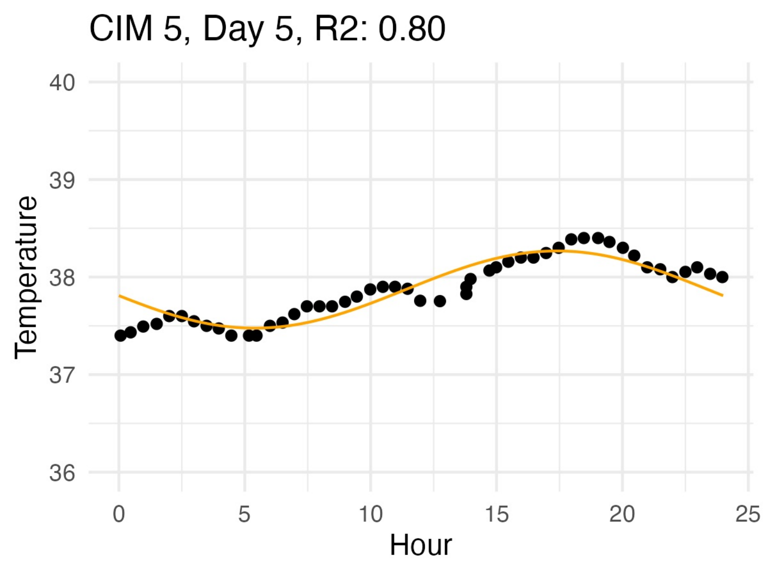
CIM 5, Day 5, R2: 0.80
Temperature
40
39
38
37
36
Hour
0
5
10
15
20
25

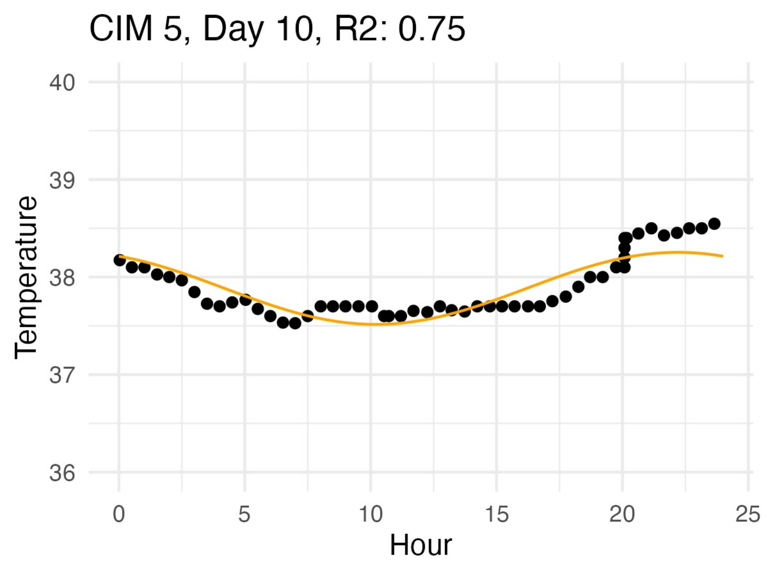

CIM 5, Day 10, R2: 0.75
Temperature
40
39
38
37
36
0
5
10
15
20
25
Hour

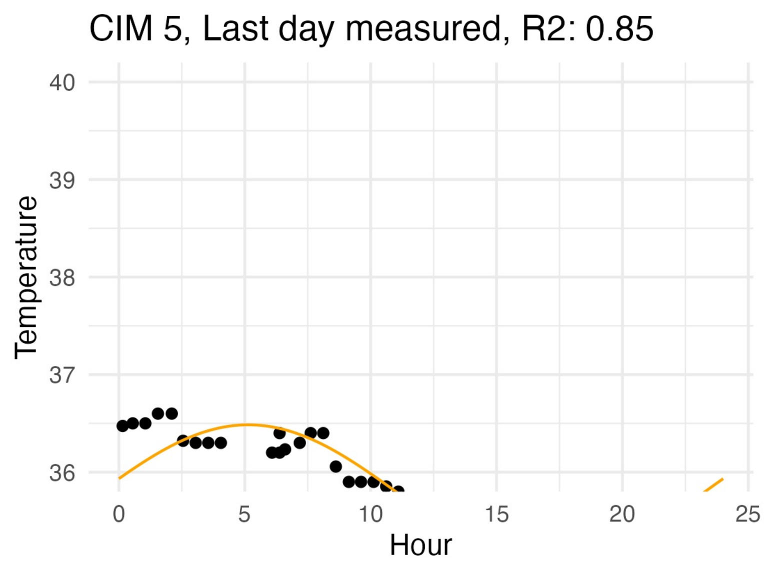
CIM 5, Last day measured, R2: 0.85
Temperature
40
39
38
37
36
0
5
10
15
20
25
Hour

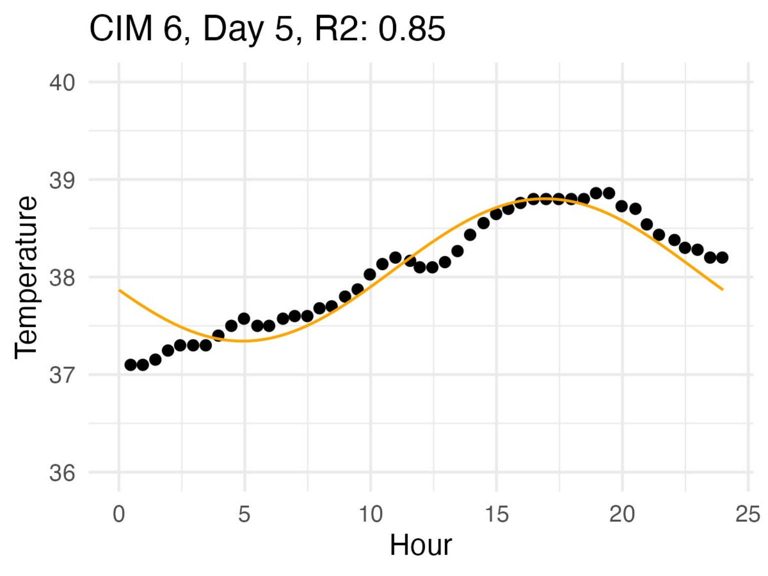

CIM 6, Day 5, R2: 0.85
Temperature
Hour
40
39
38
37
36
0
5
10
15
20
25

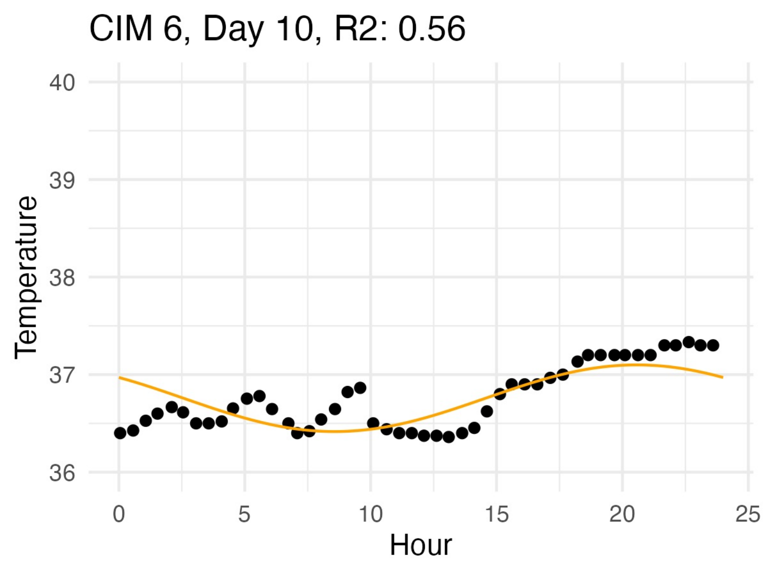
CIM 6, Day 10, R2: 0.56
Temperature
40
39
38
37
36
Hour
0
5
10
15
20
25

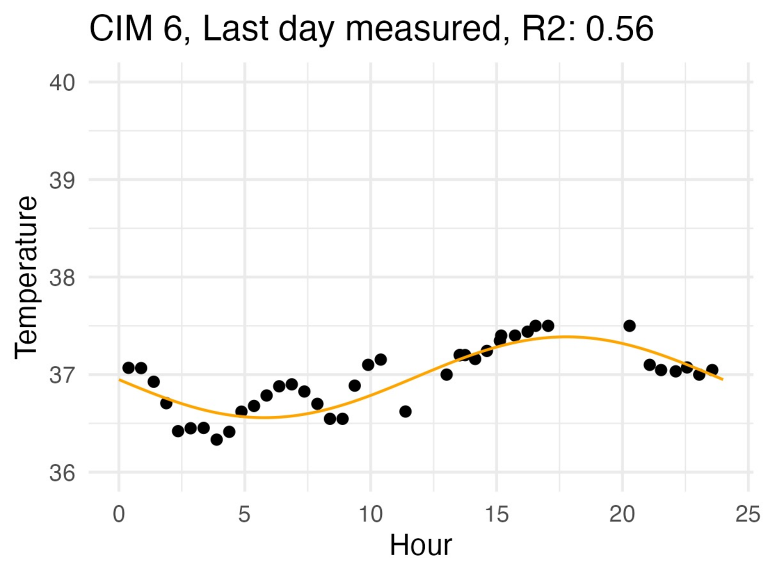
CIM 6, Last day measured, R2: 0.56
Temperature
40
39
38
37
36
0
5
10
15
20
25
Hour

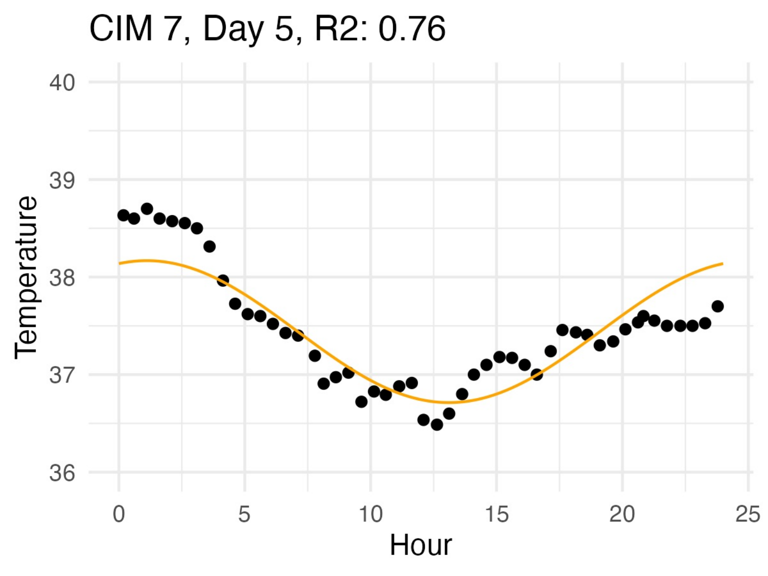
CIM 7, Day 5, R2: 0.76
Temperature
40
39
38
37
36
0
5
10
15
20
25
Hour

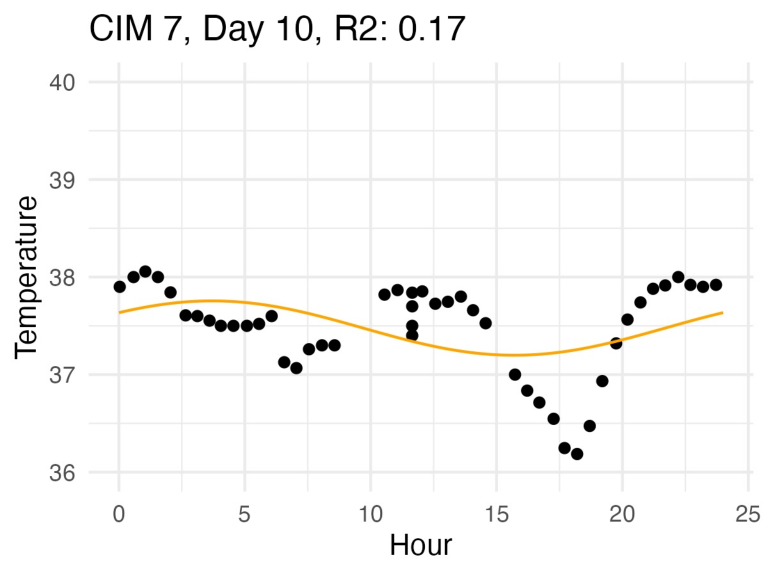
CIM 7, Day 10, R2: 0.17
Temperature
40
39
38
37
36
Hour
0
5
10
15
20
25

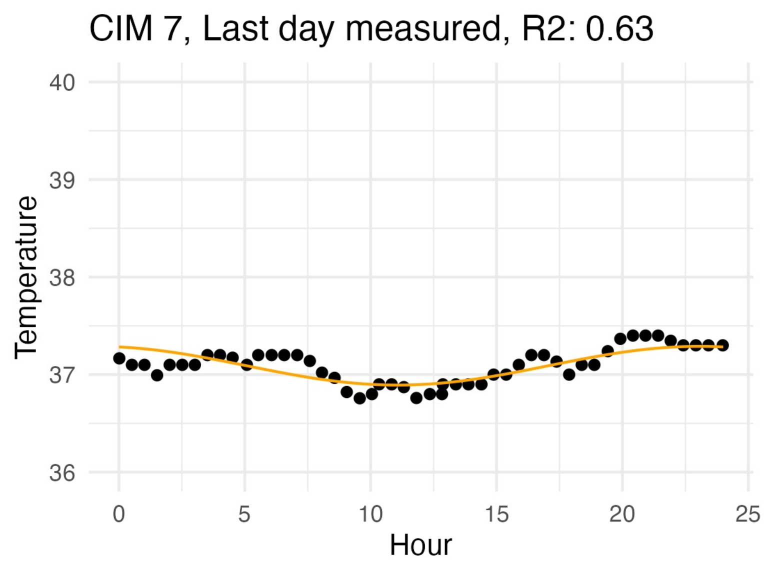
CIM 7, Last day measured, R2: 0.63
Temperature
40
39
38
37
36
0
5
10
15
20
25
Hour

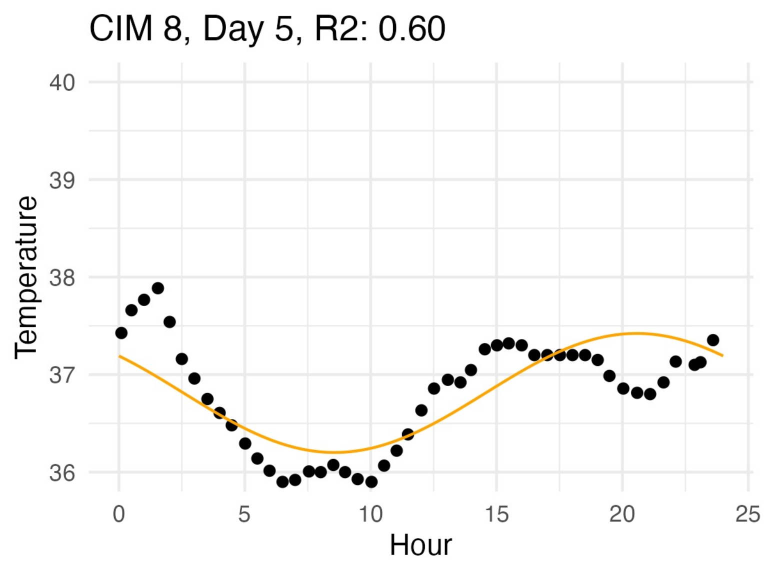
CIM 8, Day 5, R2: 0.60
Temperature
40
39
38
37
36
0
5
10
15
20
25
Hour

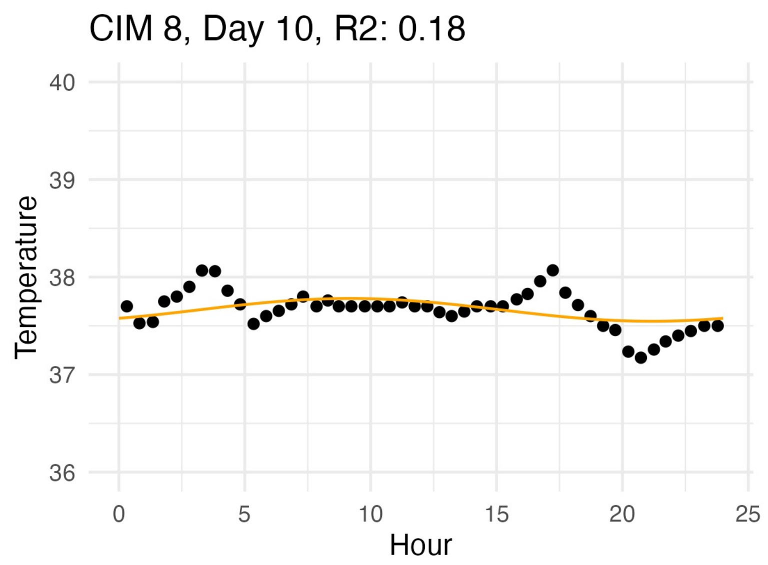
CIM 8, Day 10, R2: 0.18
40
39
38
37
36
Temperature
0
5
10
15
20
25
Hour

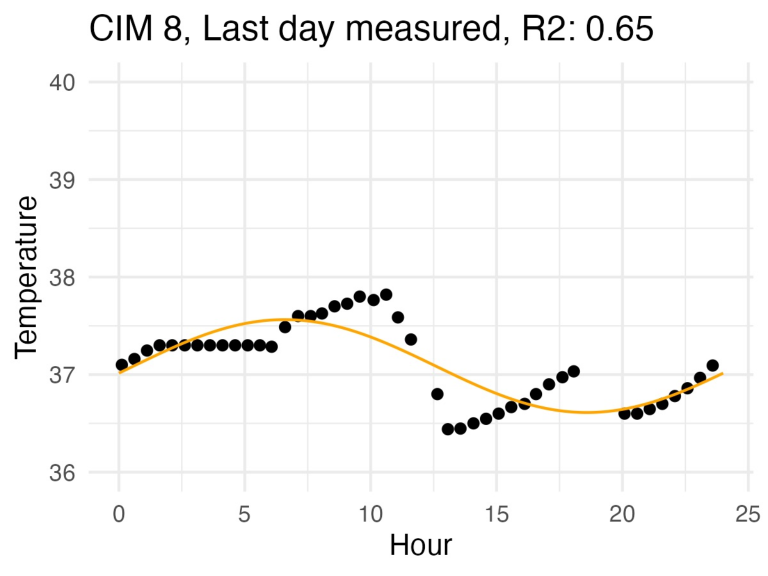
CIM 8, Last day measured, R2: 0.65
Temperature
40
39
38
37
36
Hour
0
5
10
15
20
25

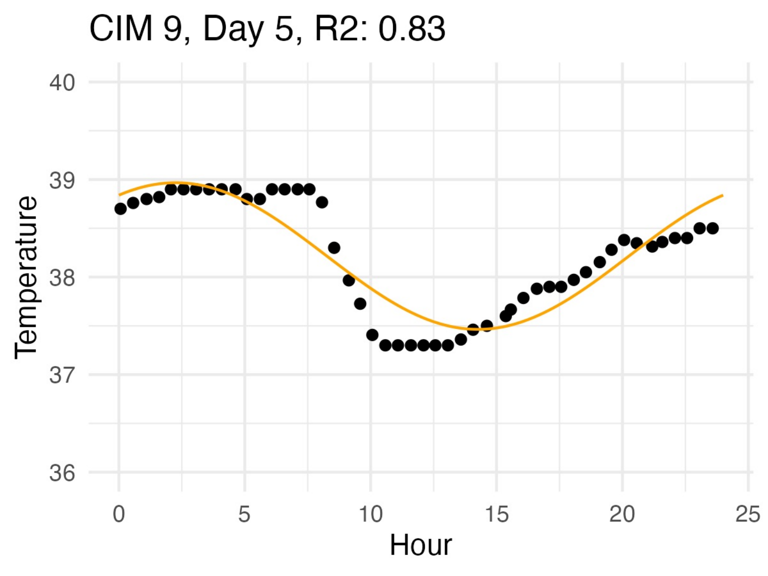
CIM 9, Day 5, R2: 0.83
Temperature
40
39
38
37
36
Hour
0
5
10
15
20
25

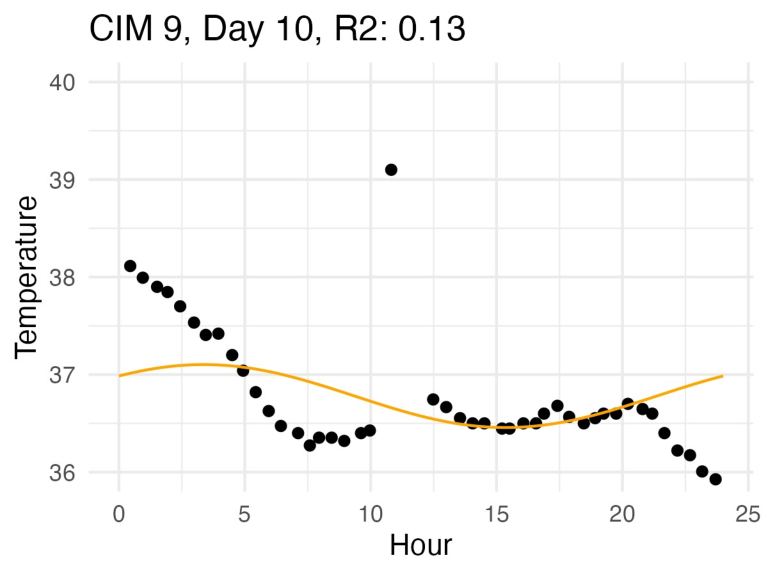

CIM 9, Day 10, R2: 0.13
Temperature
40
39
38
37
36
Hour
0
5
10
15
20
25

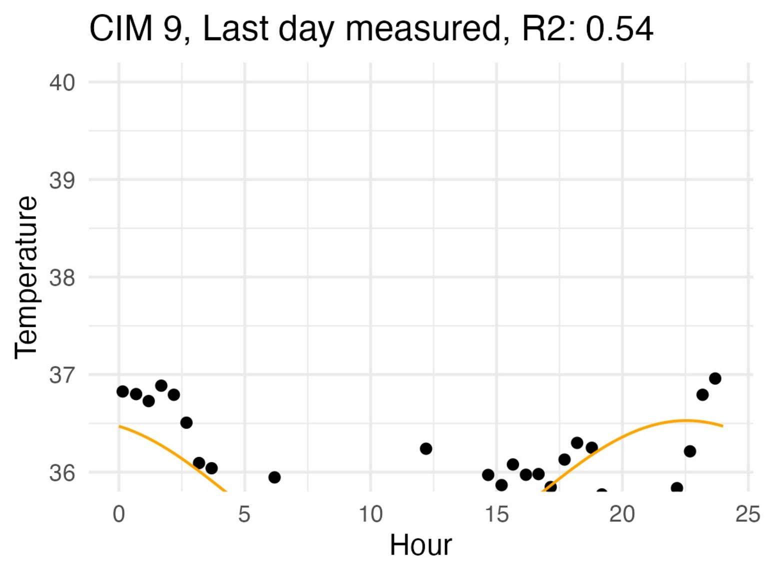
CIM 9, Last day measured, R2: 0.54
Temperature
40
39
38
37
36
0
5
10
15
20
25
Hour

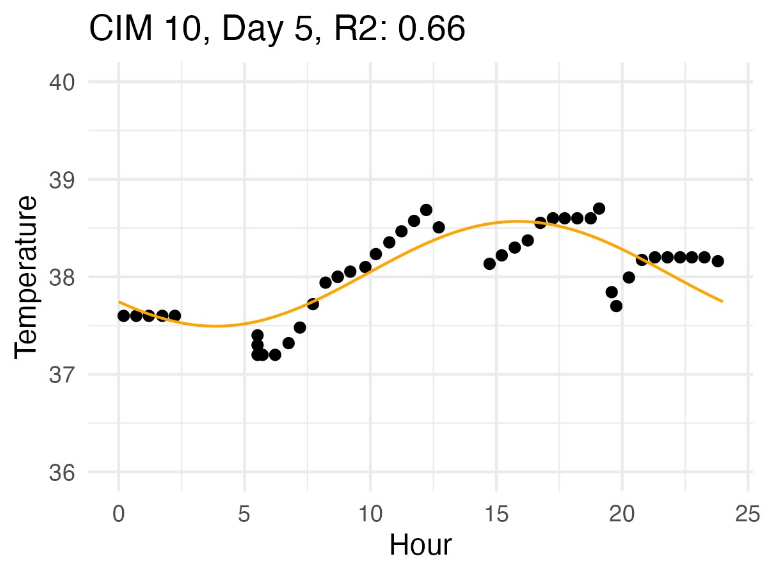
CIM 10, Day 5, R2: 0.66
Temperature
40
39
38
37
36
0
5
10
15
20
25
Hour

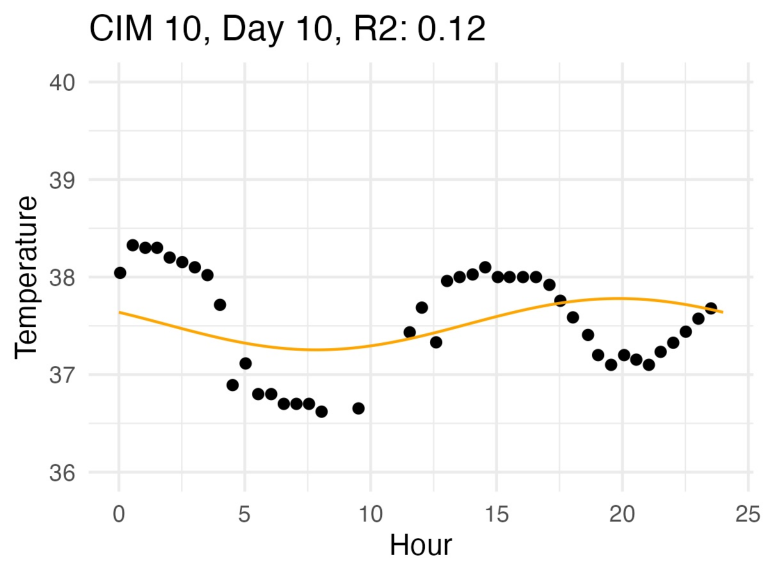

CIM 10, Day 10, R2: 0.12
Temperature
40
39
38
37
36
0
5
10
15
20
25
Hour

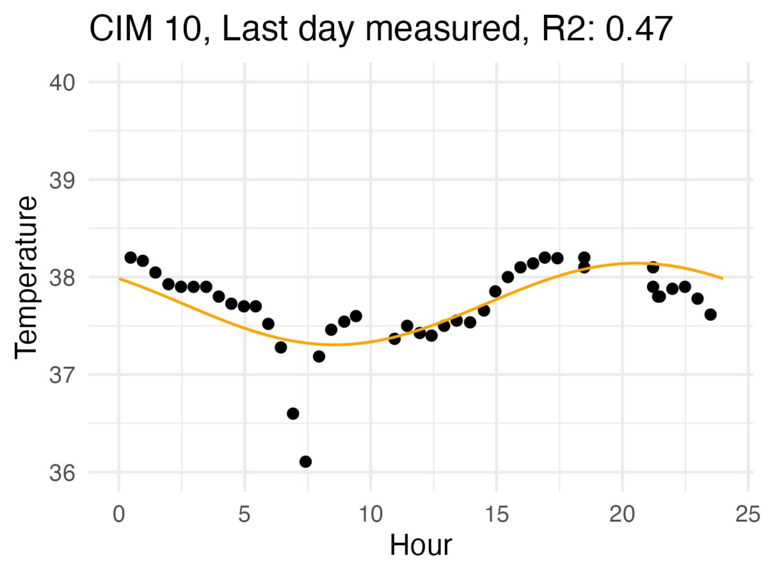

CIM 10, Last day measured, R2: 0.47
40
39
38
37
36
Temperature
0
5
10
15
20
25
Hour

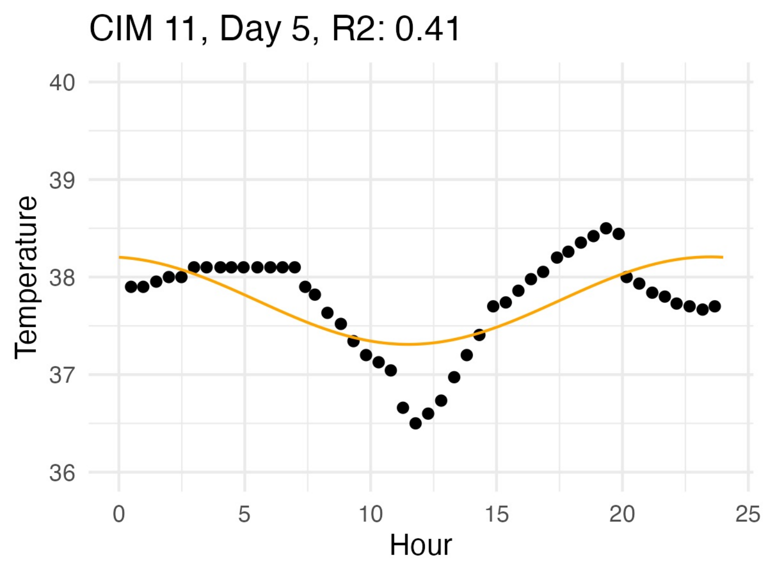

CIM 11, Day 5, R2: 0.41
40
39
38
37
36
Temperature
0
5
10
15
20
25
Hour

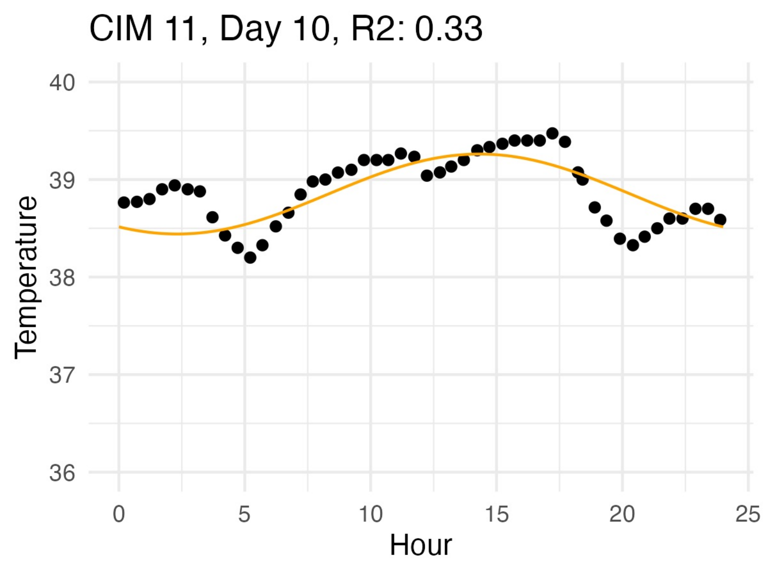

CIM 11, Day 10, R2: 0.33
Temperature
40
39
38
37
36
Hour
0
5
10
15
20
25

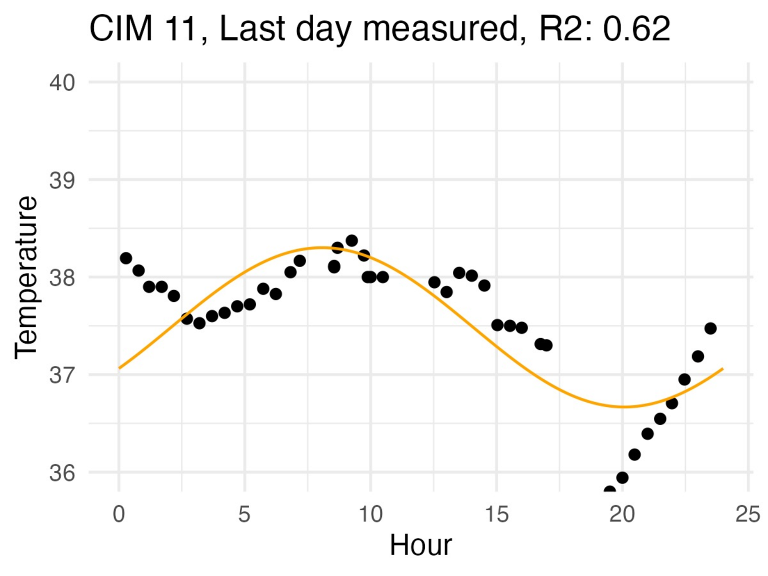

CIM 11, Last day measured, R2: 0.62
40
39
38
37
36
Temperature
0
5
10
15
20
25
Hour

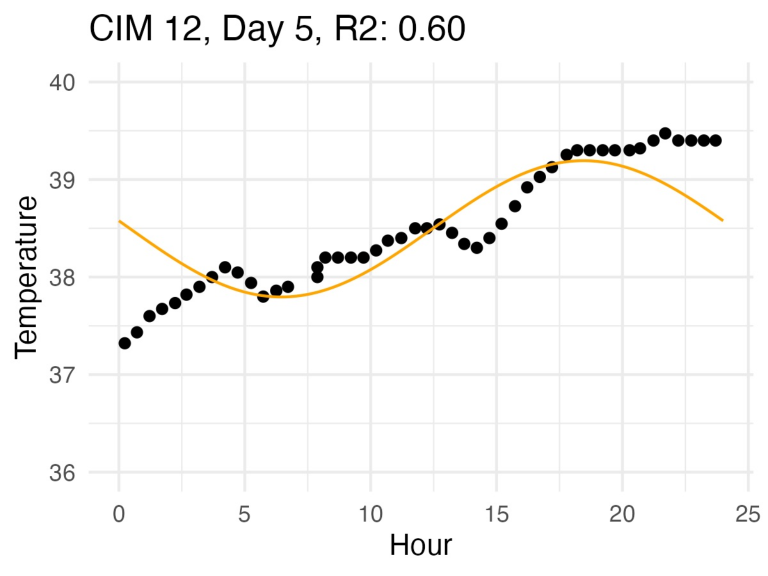
CIM 12, Day 5, R2: 0.60
Temperature
40
39
38
37
36
0
5
10
15
20
25
Hour

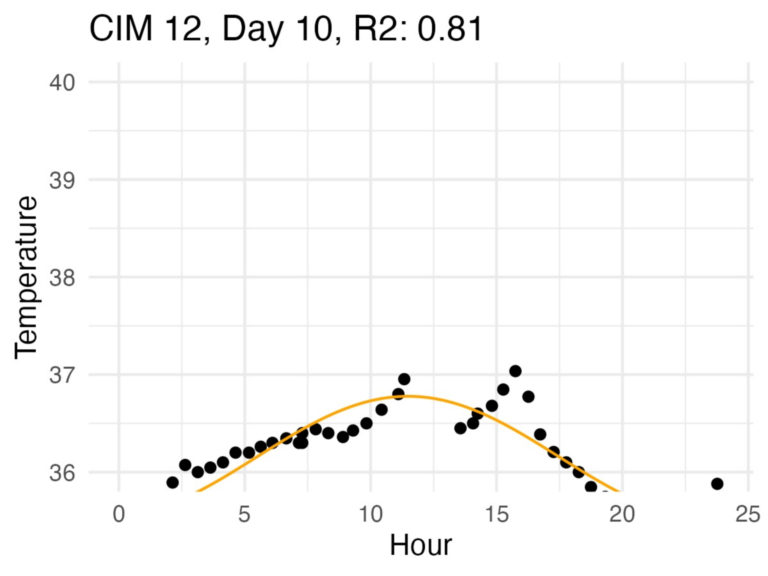
CIM 12, Day 10, R2: 0.81
Temperature
40
39
38
37
36
0
5
10
15
20
25
Hour

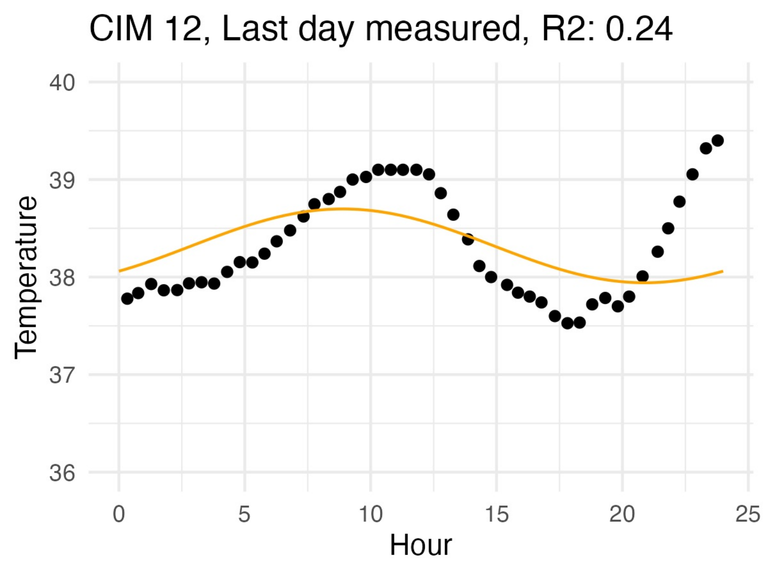
CIM 12, Last day measured, R2: 0.24
Temperature
40
39
38
37
36
0
5
10
15
20
25
Hour

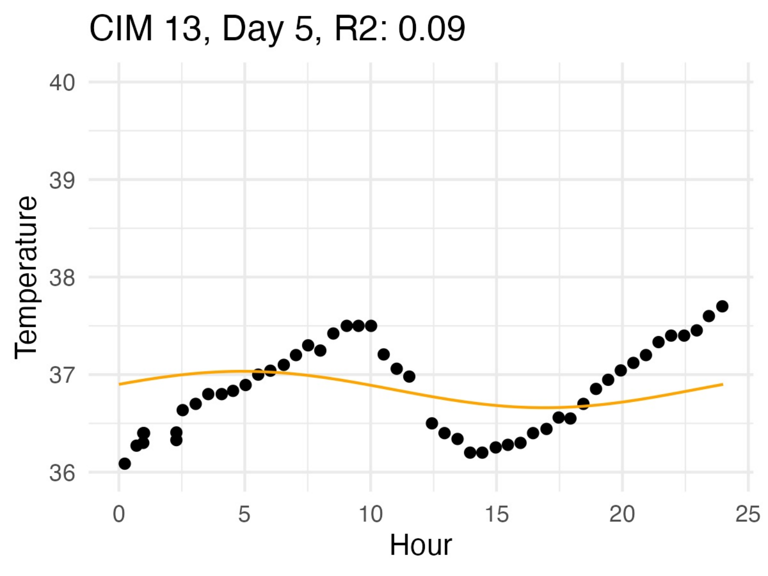
CIM 13, Day 5, R2: 0.09
Temperature
40
39
38
37
36
0
5
10
15
20
25
Hour

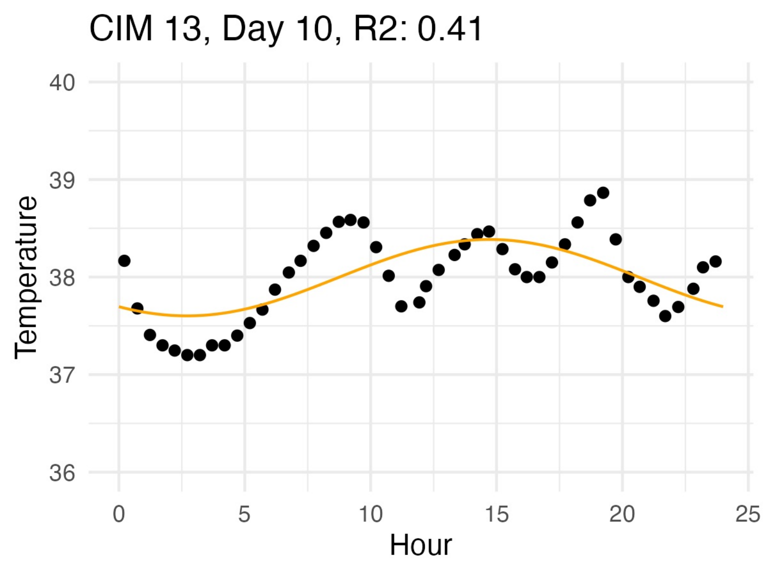
CIM 13, Day 10, R2: 0.41
Temperature
40
39
38
37
36
0
5
10
15
20
25
Hour

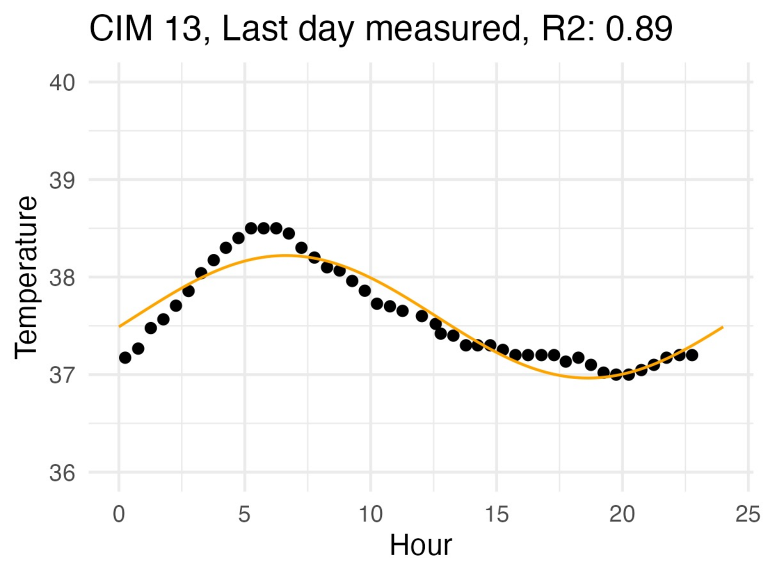

CIM 13, Last day measured, R2: 0.89
Temperature
40
39
38
37
36
0
5
10
15
20
25
Hour

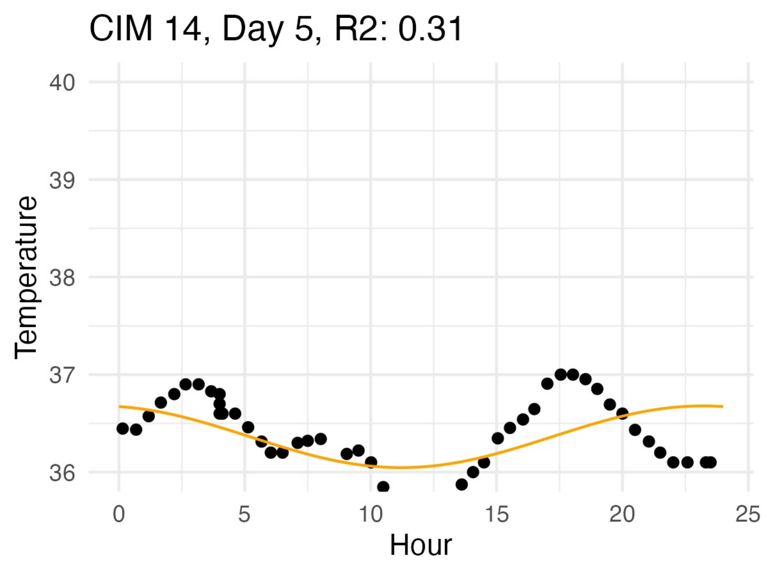

CIM 14, Day 5, R2: 0.31
Temperature
40
39
38
37
36
0
5
10
15
20
25
Hour

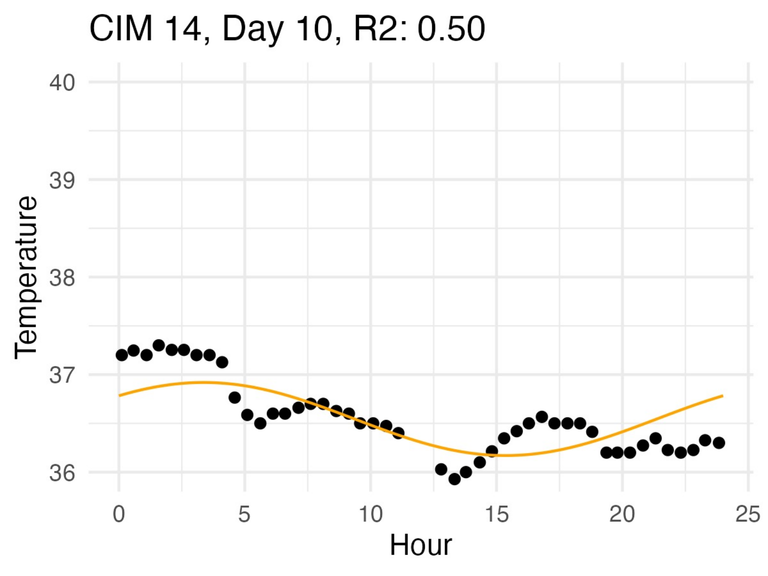
CIM 14, Day 10, R2: 0.50
40
39
38
37
36
Temperature
0
5
10
15
20
25
Hour

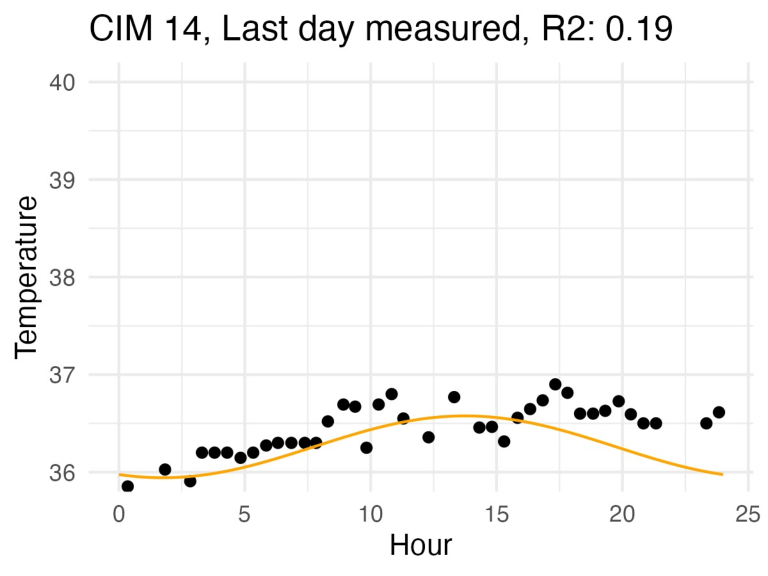
CIM 14, Last day measured, R2: 0.19
Temperature
40
39
38
37
36
0
5
10
15
20
25
Hour

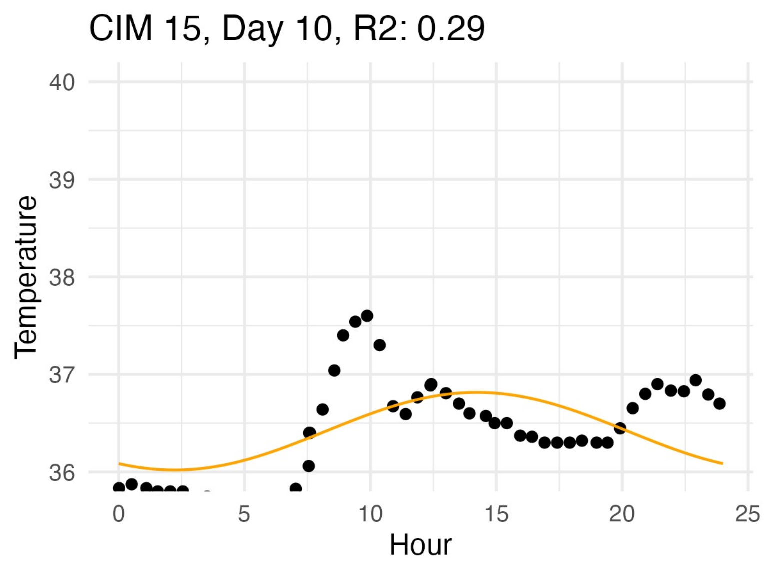

CIM 15, Day 10, R2: 0.29
Temperature
40
39
38
37
36
0
5
10
15
20
25
Hour

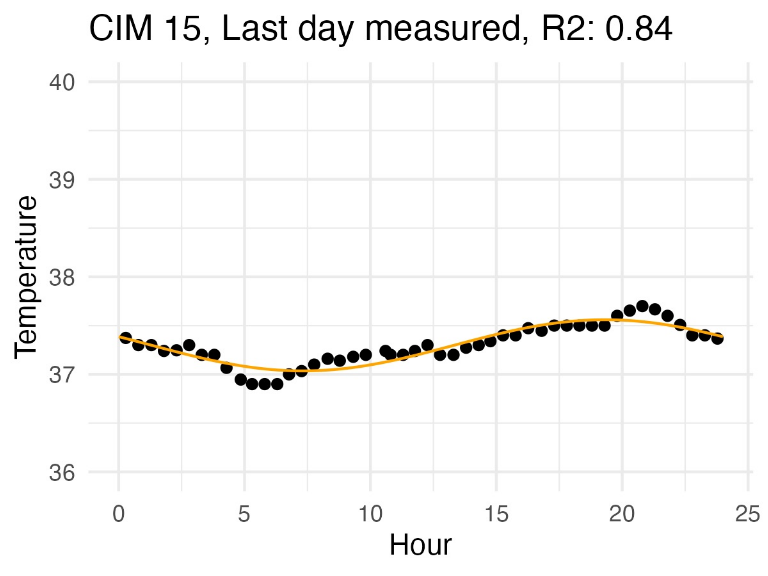

CIM 15, Last day measured, R2: 0.84
Temperature
40
39
38
37
36
Hour
0
5
10
15
20
25

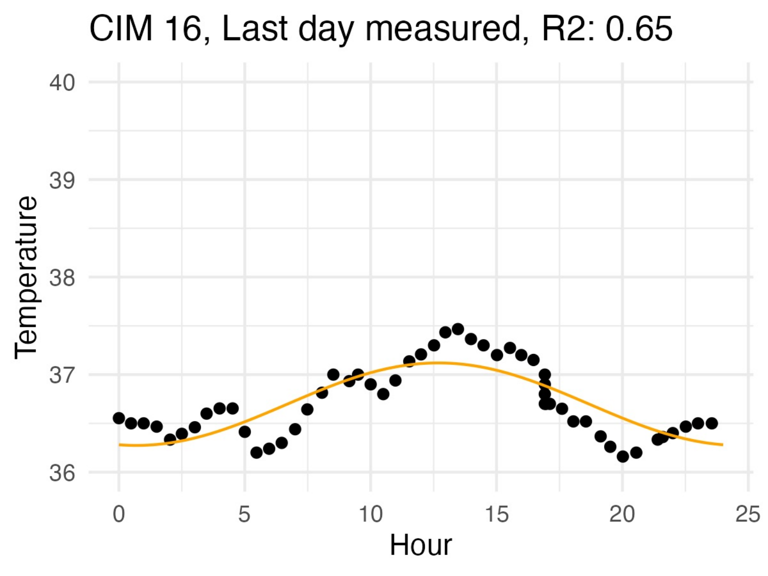
CIM 16, Last day measured, R2: 0.65
40
39
38
37
36
Temperature
0
5
10
15
20
25
Hour

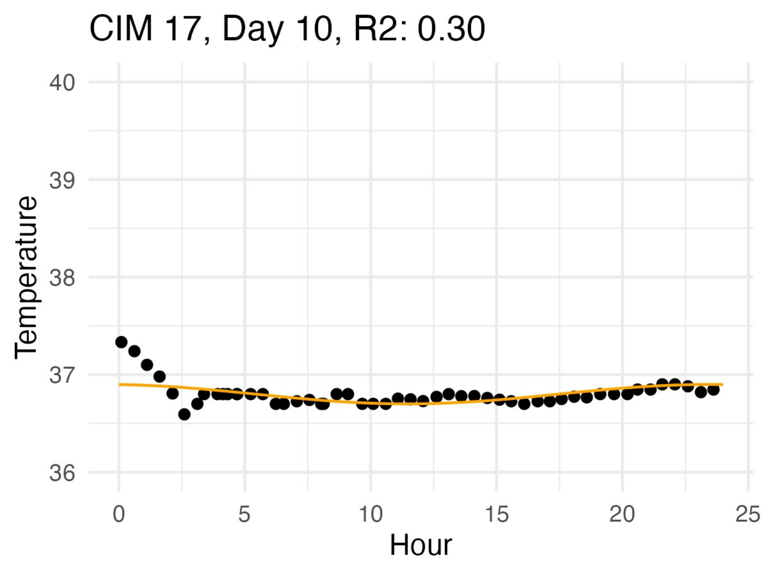
CIM 17, Day 10, R2: 0.30
Temperature
40
39
38
37
36
0
5
10
15
20
25
Hour

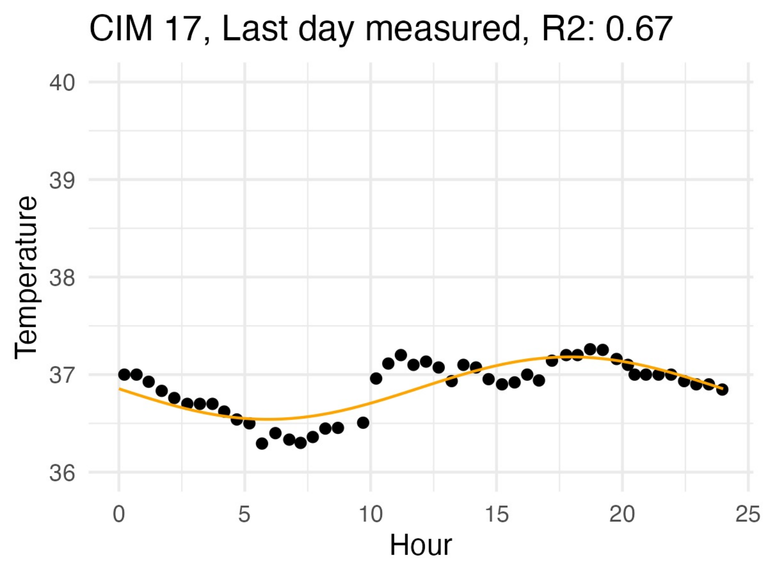
CIM 17, Last day measured, R2: 0.67
Temperature
40
39
38
37
36
0
5
10
15
20
25
Hour

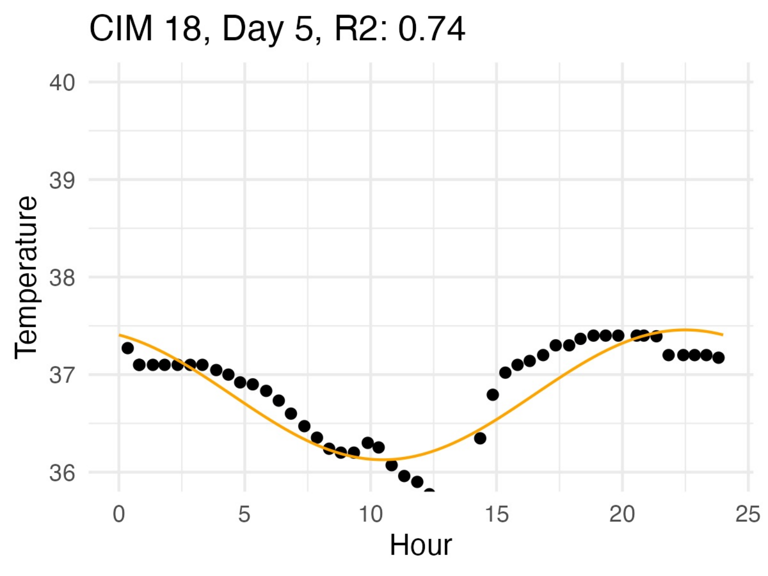

CIM 18, Day 5, R2: 0.74
Temperature
40
39
38
37
36
0
5
10
15
20
25
Hour

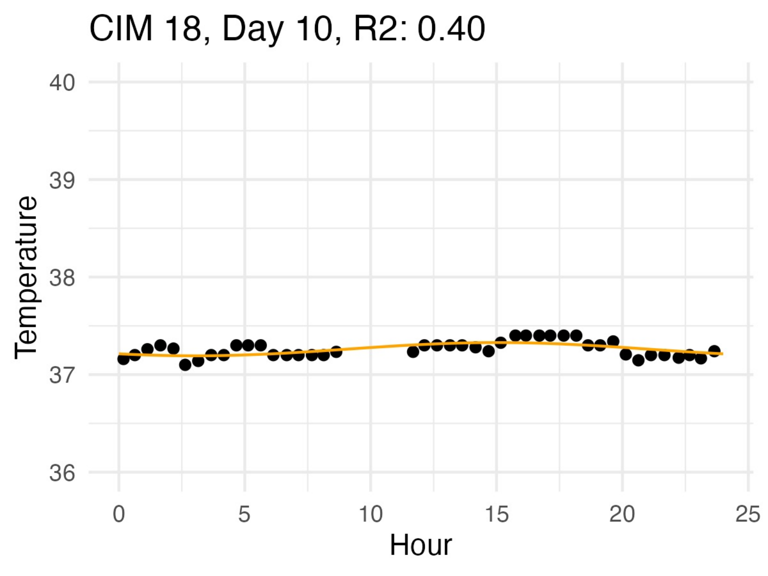

CIM 18, Day 10, R2: 0.40
Temperature
Hour
40
39
38
37
36
0
5
10
15
20
25

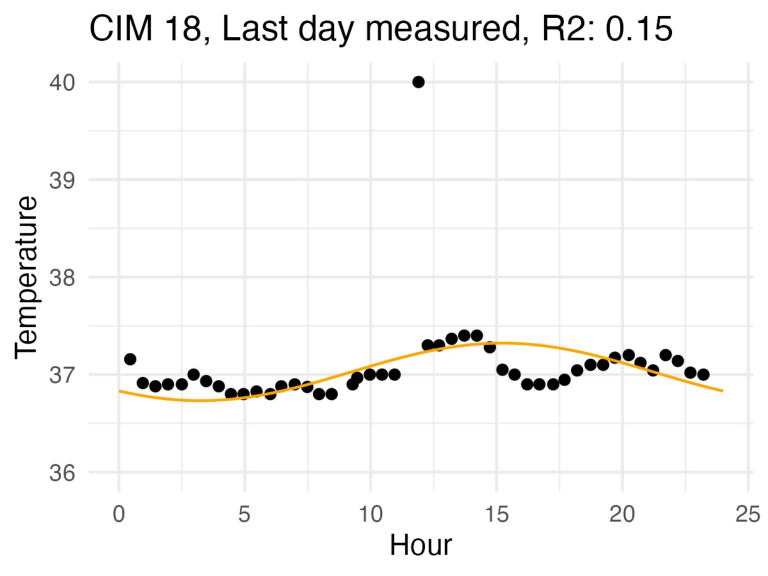
CIM 18, Last day measured, R2: 0.15
Temperature
40
39
38
37
36
Hour
0
5
10
15
20
25

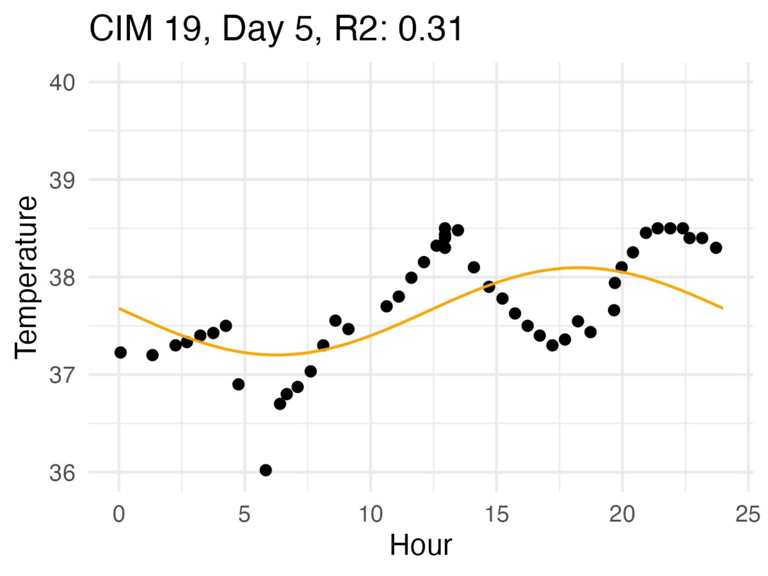

CIM 19, Day 5, R2: 0.31
40
39
38
37
36
Temperature
0
5
10
15
20
25
Hour

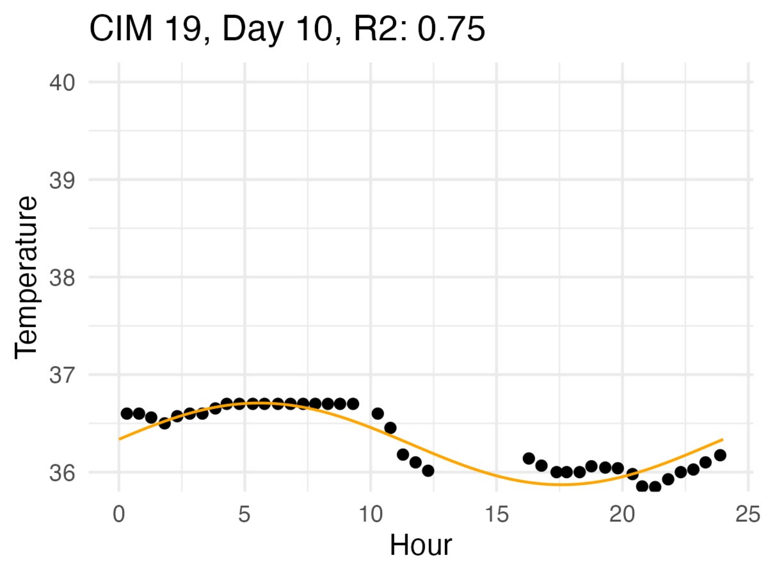
CIM 19, Day 10, R2: 0.75
Temperature
40
39
38
37
36
0
5
10
15
20
25
Hour

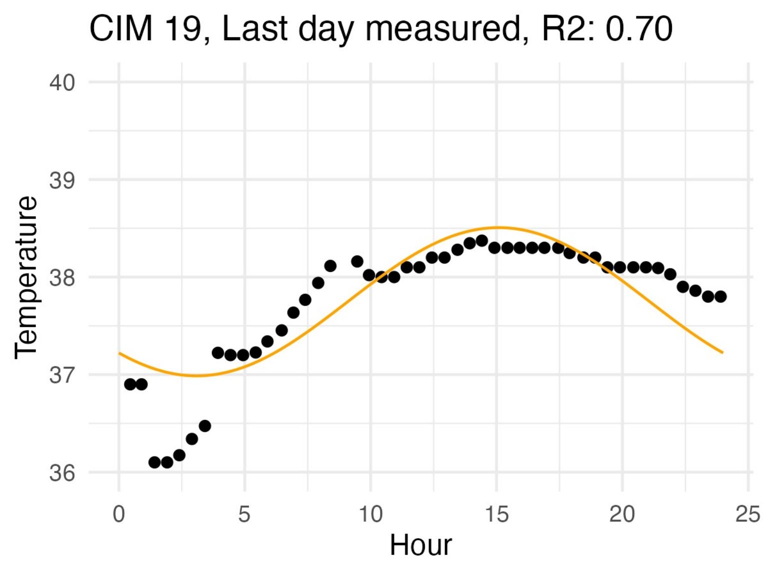
CIM 19, Last day measured, R2: 0.70
Temperature
40
39
38
37
36
0
5
10
15
20
25
Hour

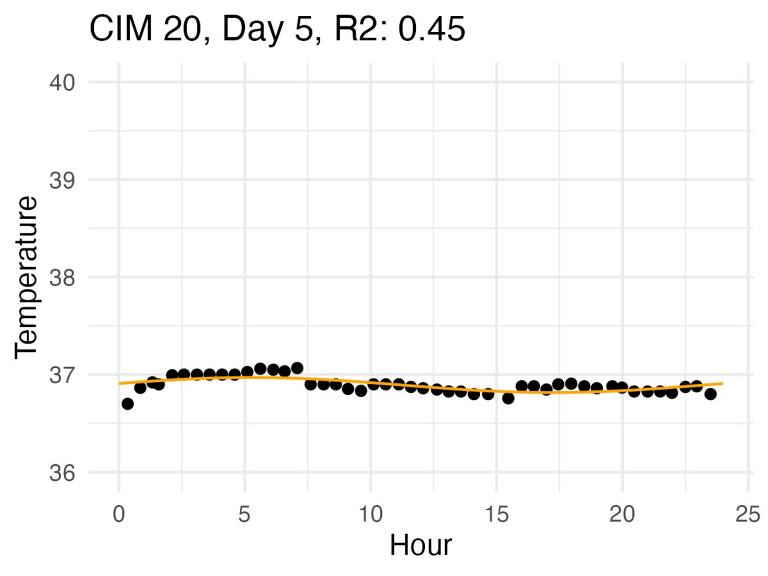
CIM 20, Day 5, R2: 0.45
Temperature
40
39
38
37
36
0
5
10
15
20
25
Hour

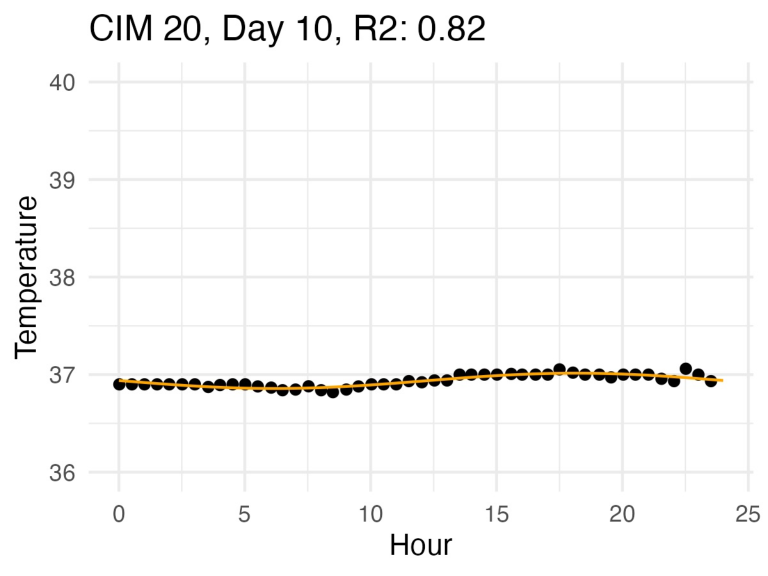

CIM 20, Day 10, R2: 0.82
Temperature
40
39
38
37
36
0
5
10
15
20
25
Hour

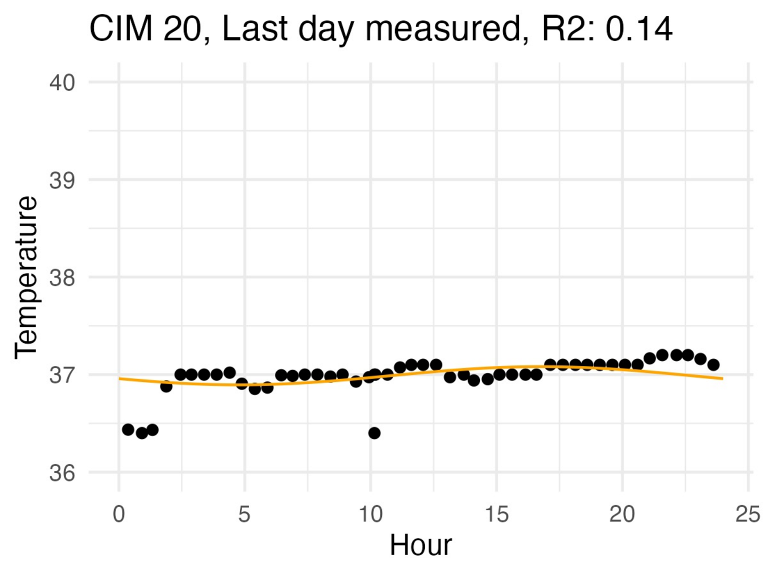

CIM 20, Last day measured, R2: 0.14
Temperature
40
39
38
37
36
0
5
10
15
20
25
Hour

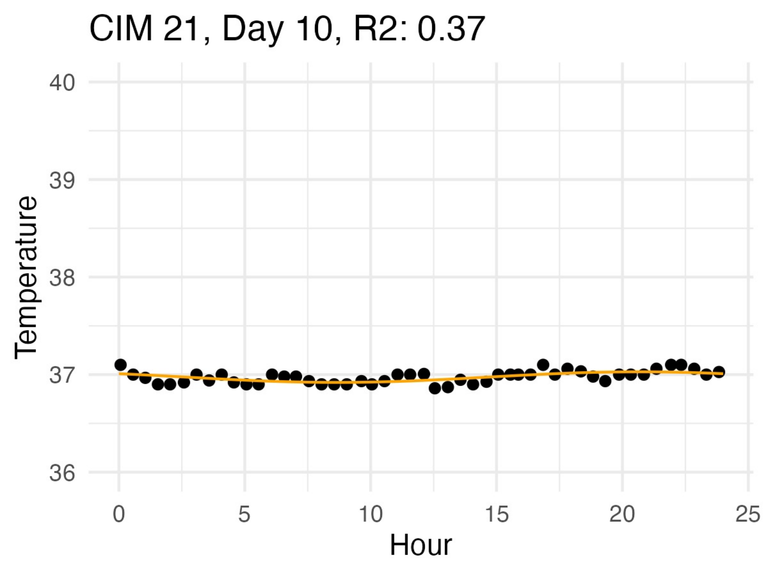

CIM 21, Day 10, R2: 0.37
Temperature
40
39
38
37
36
0
5
10
15
20
25
Hour

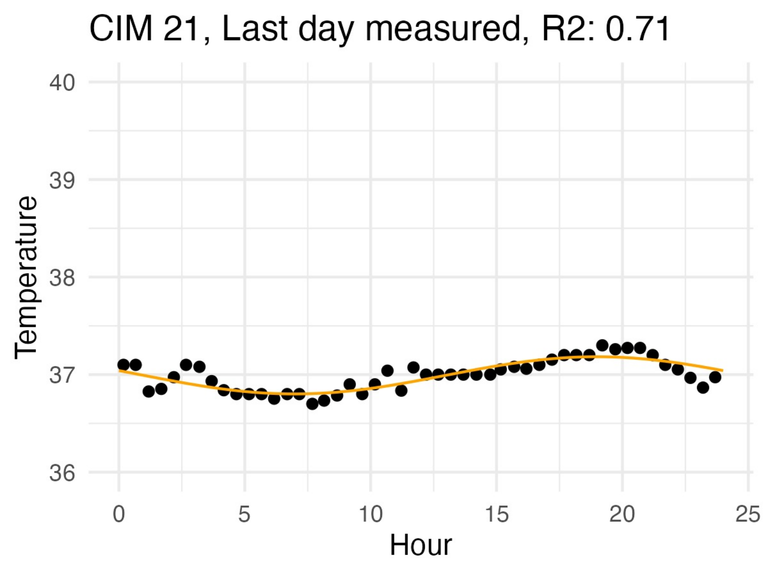
CIM 21, Last day measured, R2: 0.71
Temperature
40
39
38
37
36
Hour
0
5
10
15
20
25

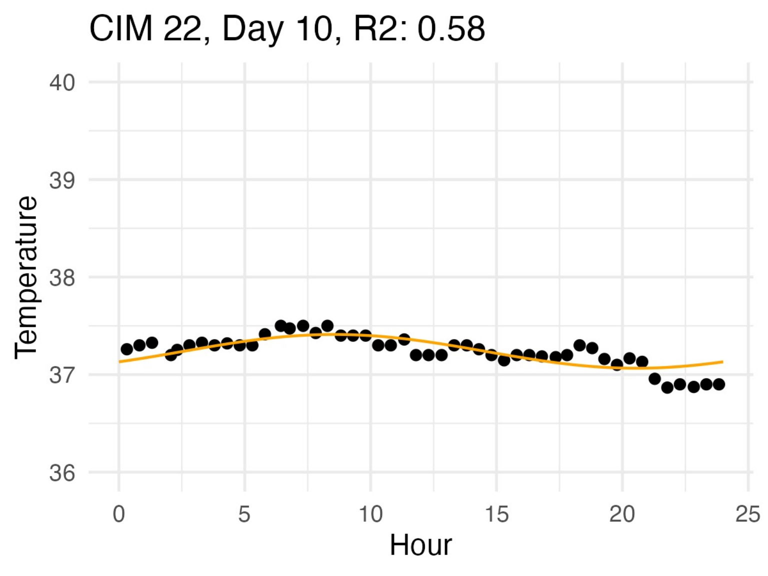
CIM 22, Day 10, R2: 0.58
Temperature
40
39
38
37
36
0
5
10
15
20
25
Hour

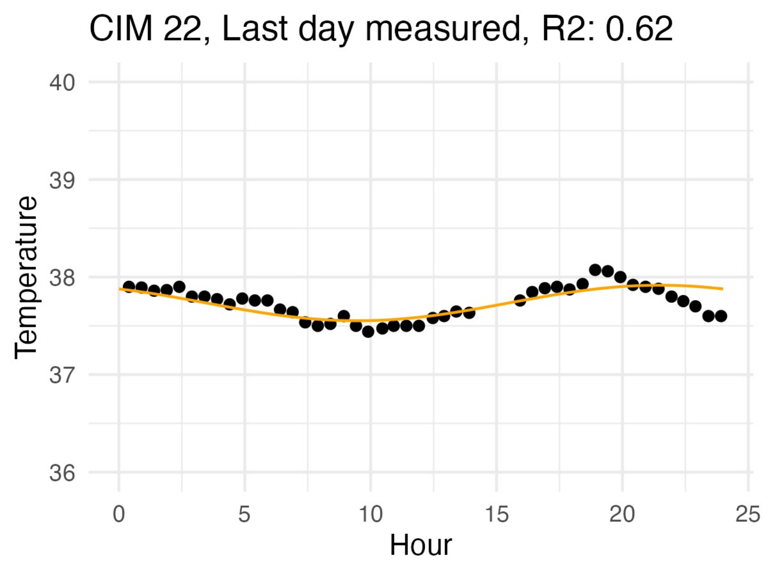
CIM 22, Last day measured, R2: 0.62
Temperature
40
39
38
37
36
Hour
0
5
10
15
20
25

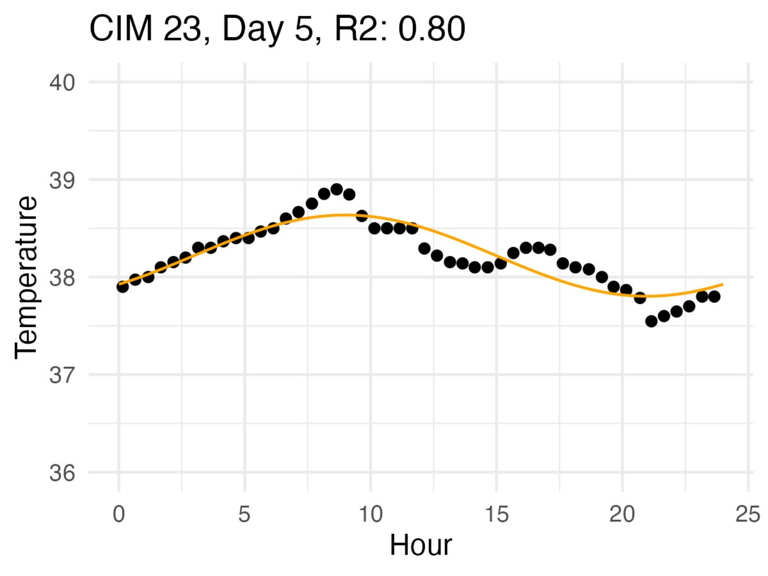
CIM 23, Day 5, R2: 0.80
Temperature
40
39
38
37
36
0
5
10
15
20
25
Hour

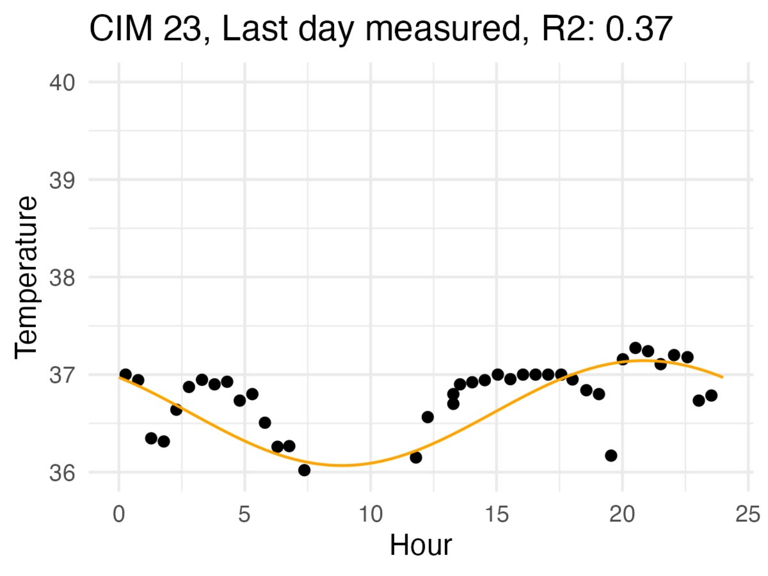
CIM 23, Last day measured, R2: 0.37
Temperature
40
39
38
37
36
0
5
10
15
20
25
Hour

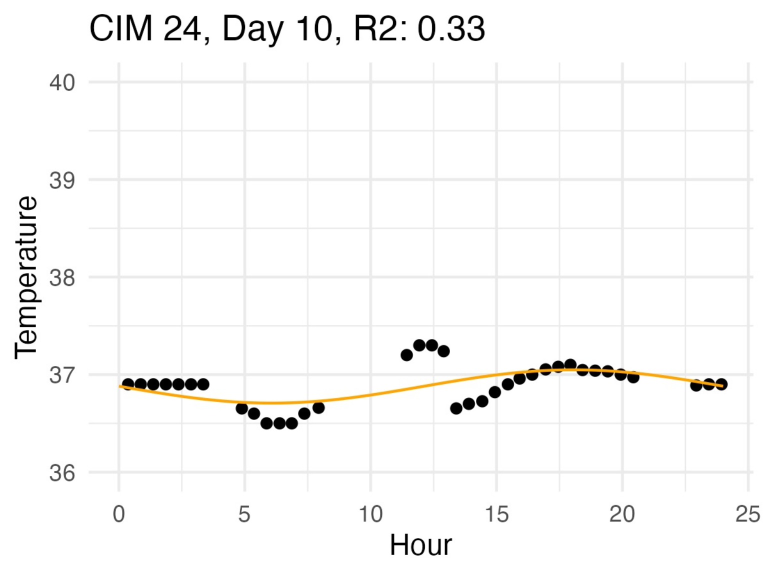
CIM 24, Day 10, R2: 0.33
Temperature
Hour
40
39
38
37
36
0
5
10
15
20
25

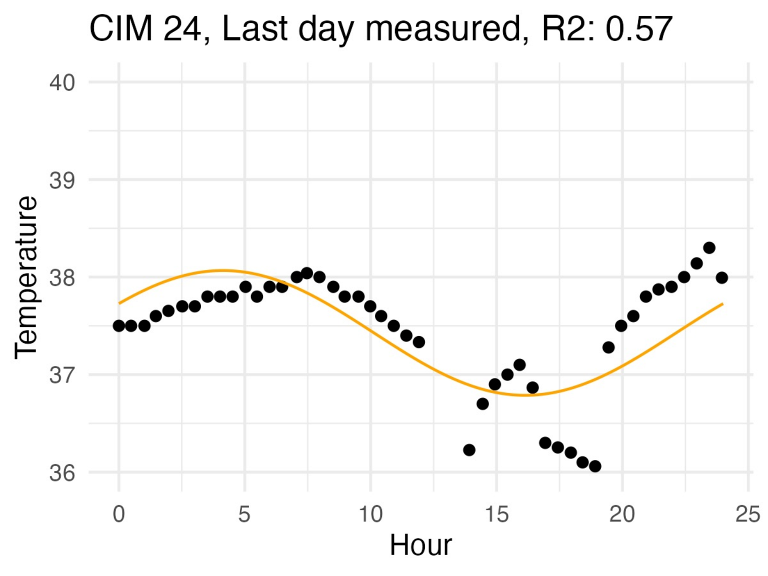
CIM 24, Last day measured, R2: 0.57
Temperature
Hour
40
39
38
37
36
0
5
10
15
20
25

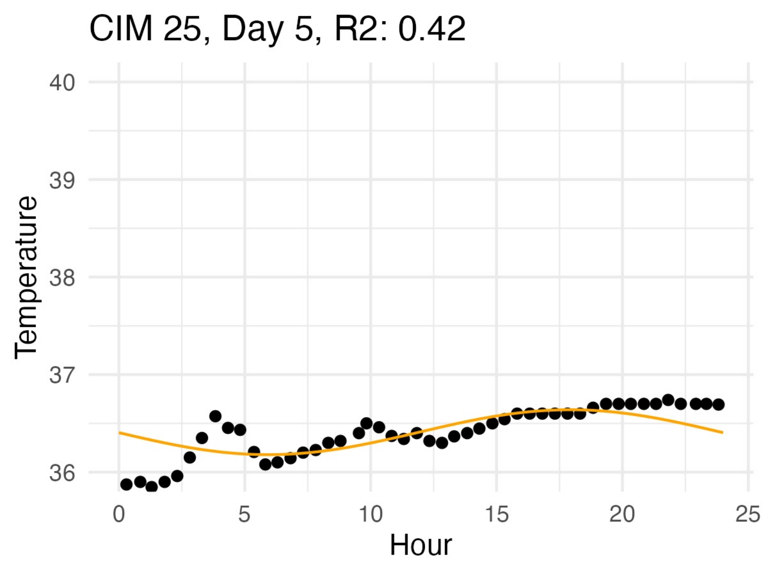
CIM 25, Day 5, R2: 0.42
Temperature
40
39
38
37
36
0
5
10
15
20
25
Hour

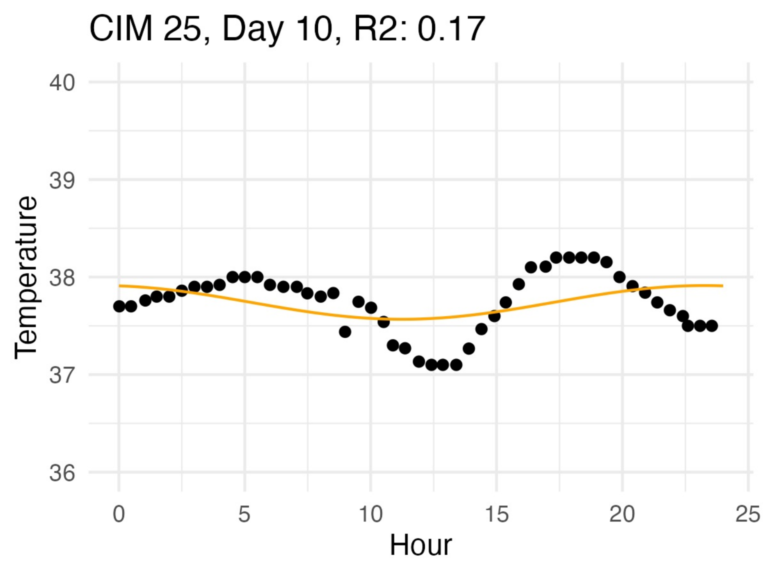

CIM 25, Day 10, R2: 0.17
Temperature
40
39
38
37
36
0
5
10
15
20
25
Hour

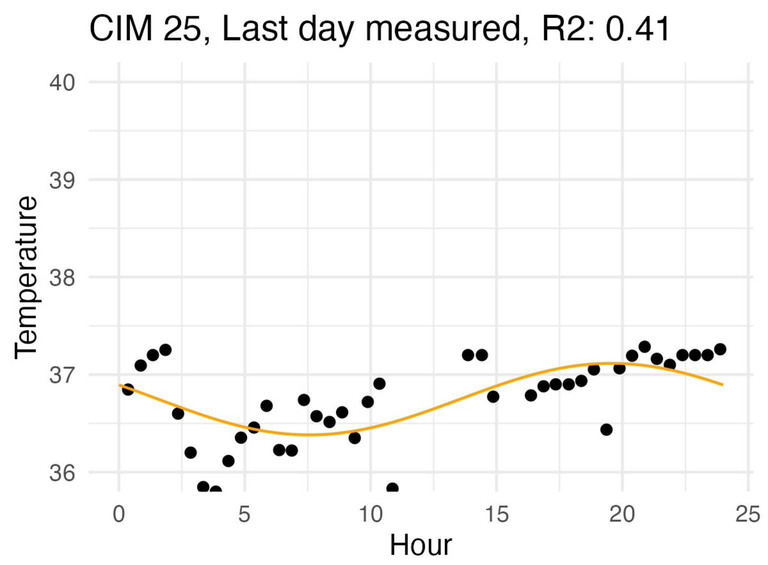
CIM 25, Last day measured, R2: 0.41
Temperature
40
39
38
37
36
0
5
10
15
20
25
Hour

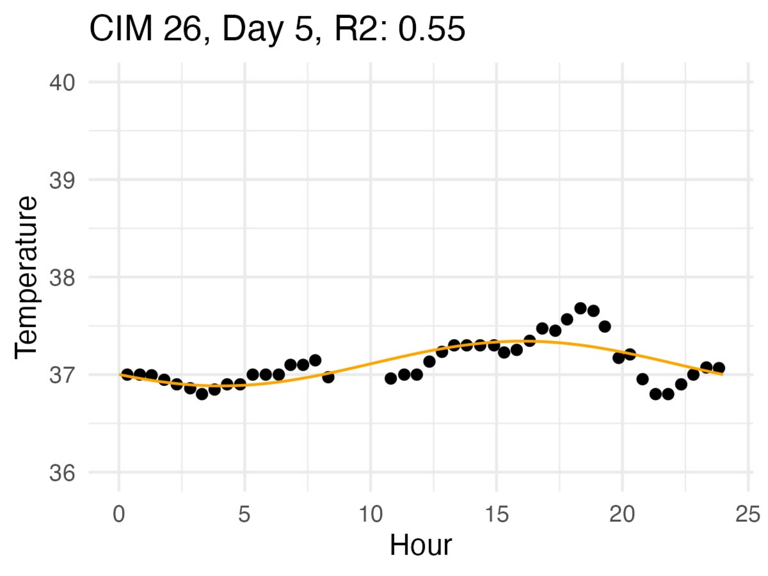
CIM 26, Day 5, R2: 0.55
Temperature
40
39
38
37
36
0
5
10
15
20
25
Hour

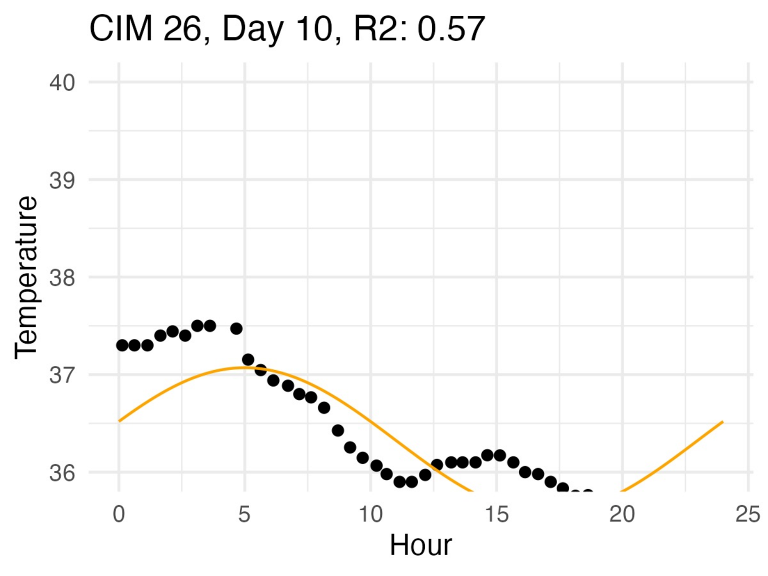
CIM 26, Day 10, R2: 0.57
Temperature
40
39
38
37
36
0
5
10
15
20
25
Hour

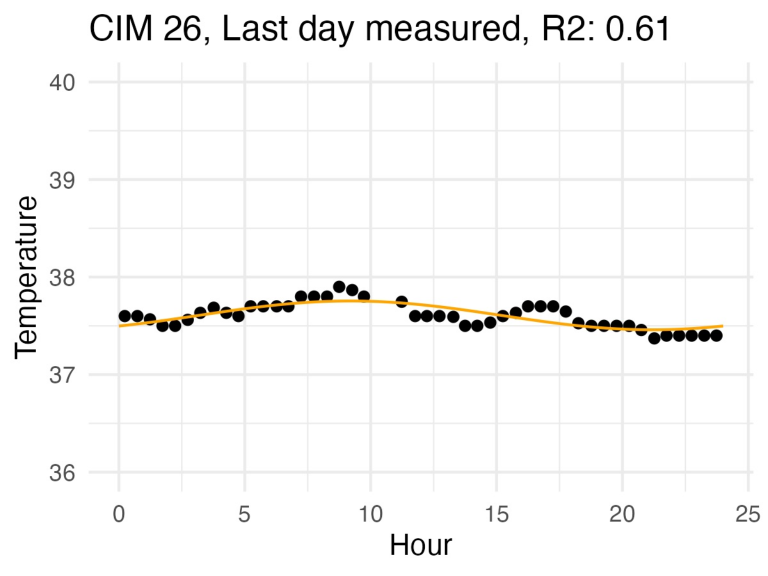

CIM 26, Last day measured, R2: 0.61
Temperature
40
39
38
37
36
Hour
0
5
10
15
20
25

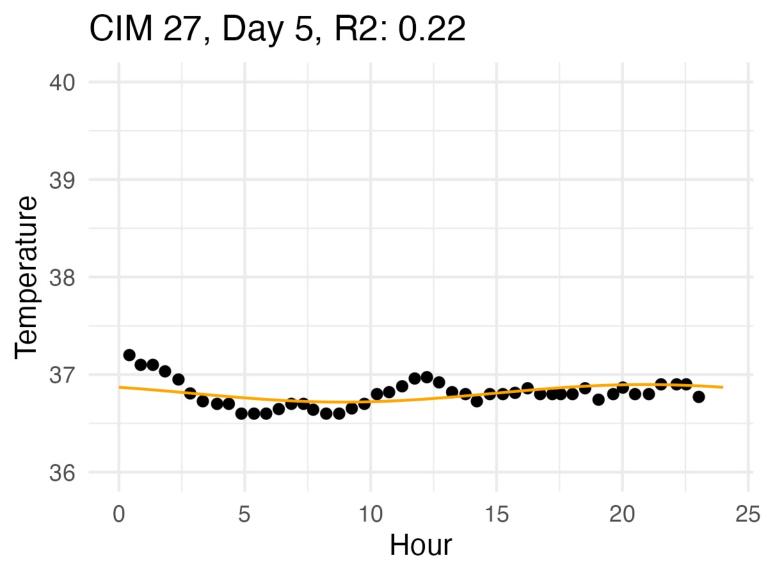
CIM 27, Day 5, R2: 0.22
Temperature
40
39
38
37
36
Hour
0
5
10
15
20
25

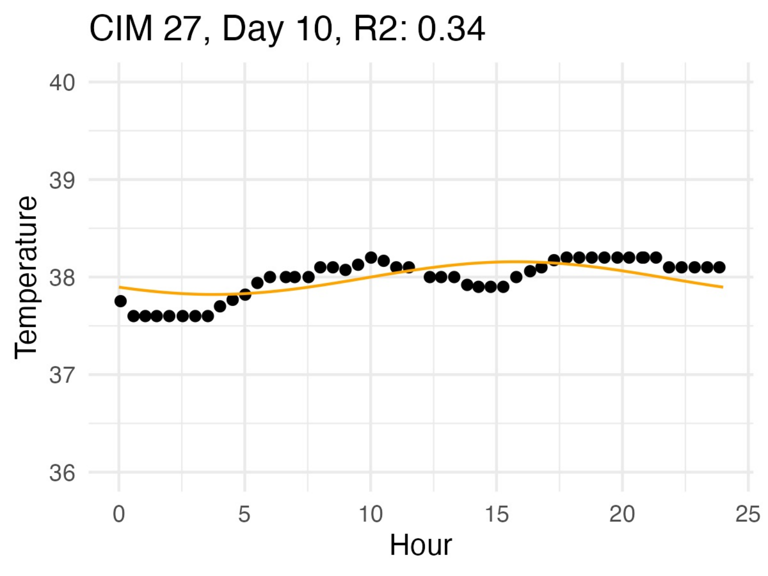
CIM 27, Day 10, R2: 0.34
Temperature
Hour
40
39
38
37
36
0
5
10
15
20
25

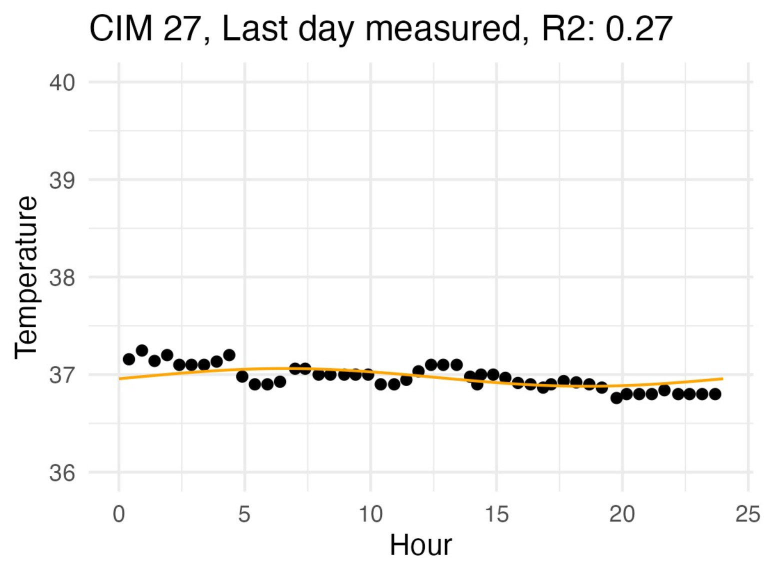
CIM 27, Last day measured, R2: 0.27
40
39
38
37
36
Temperature
0
5
10
15
20
25
Hour

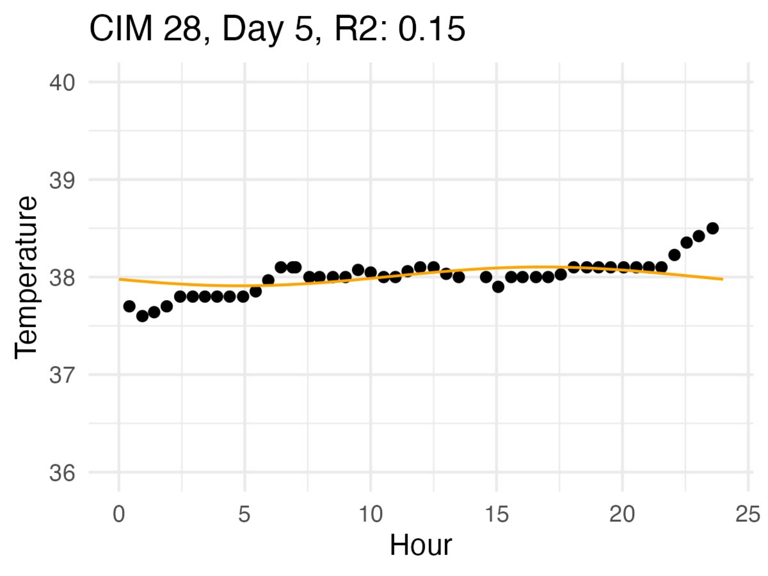

CIM 28, Day 5, R2: 0.15
Temperature
40
39
38
37
36
0
5
10
15
20
25
Hour

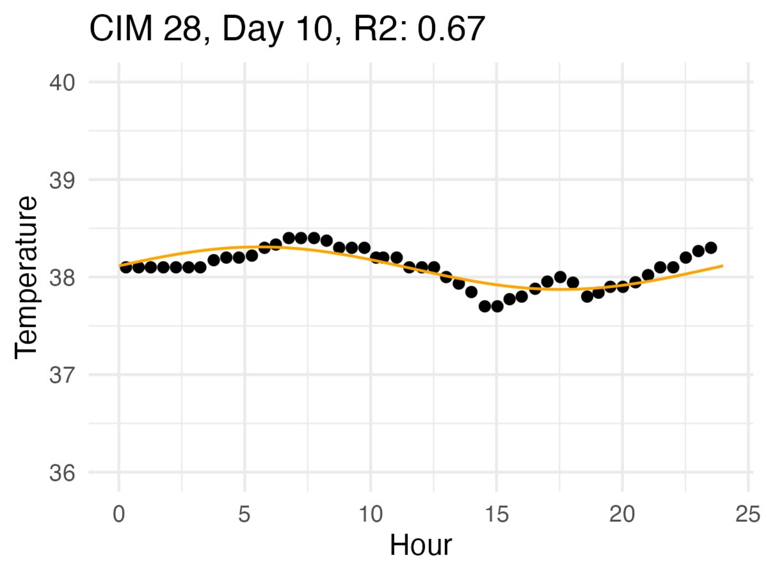

CIM 28, Day 10, R2: 0.67
Temperature
40
39
38
37
36
Hour
0
5
10
15
20
25

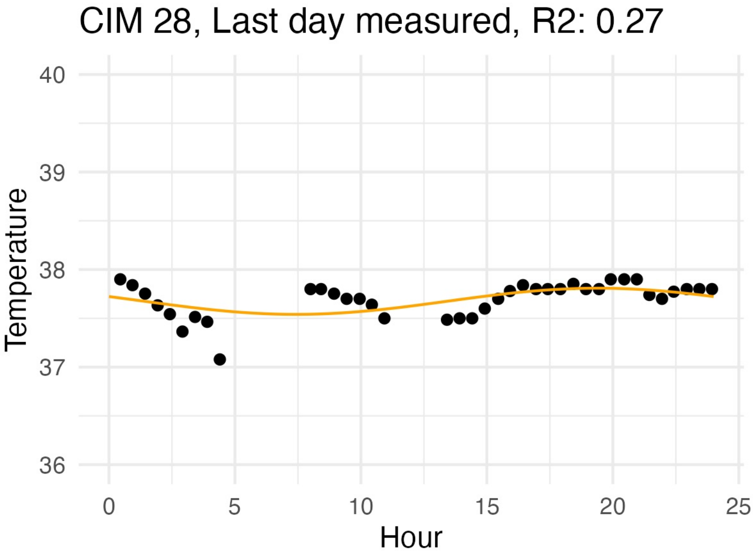
CIM 28, Last day measured, R2: 0.27
Temperature
40
39
38
37
36
0
5
10
15
20
25
Hour

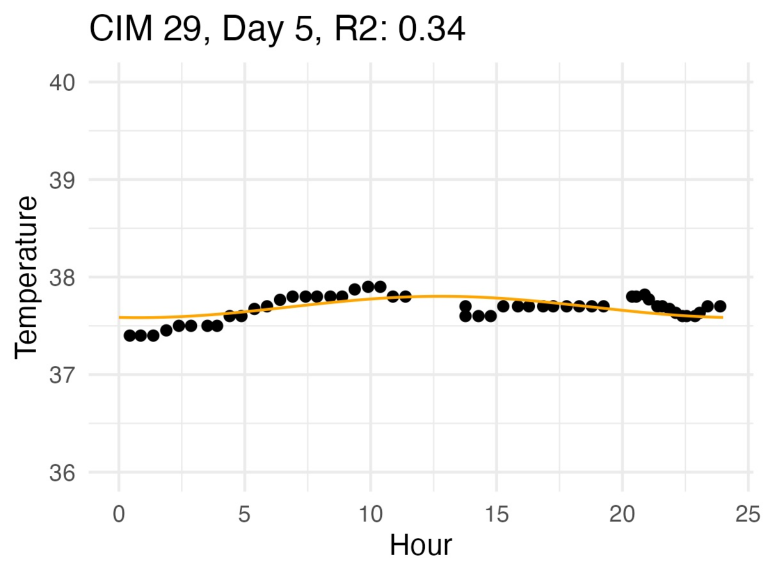
CIM 29, Day 5, R2: 0.34
Temperature
40
39
38
37
36
0
5
10
15
20
25
Hour

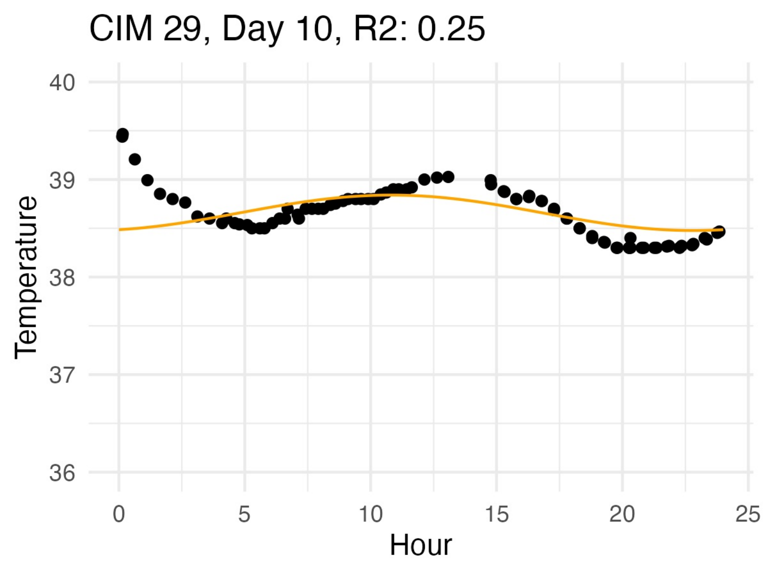
CIM 29, Day 10, R2: 0.25
Temperature
40
39
38
37
36
Hour
0
5
10
15
20
25

CIM 29, Last day measured, R2: 0.85

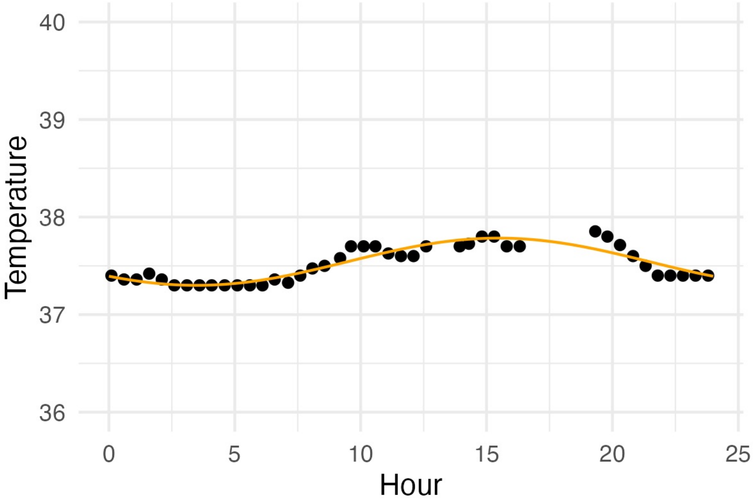

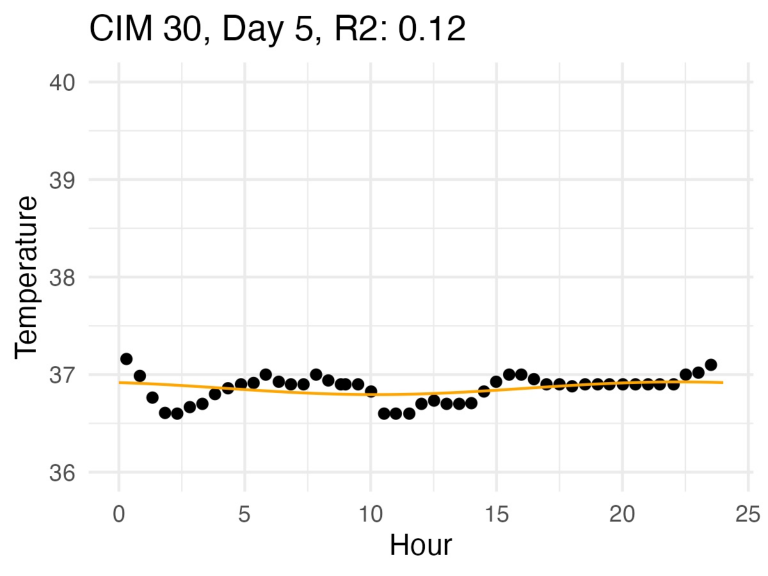
CIM 30, Day 5, R2: 0.12
Temperature
40
39
38
37
36
0
5
10
15
20
25
Hour

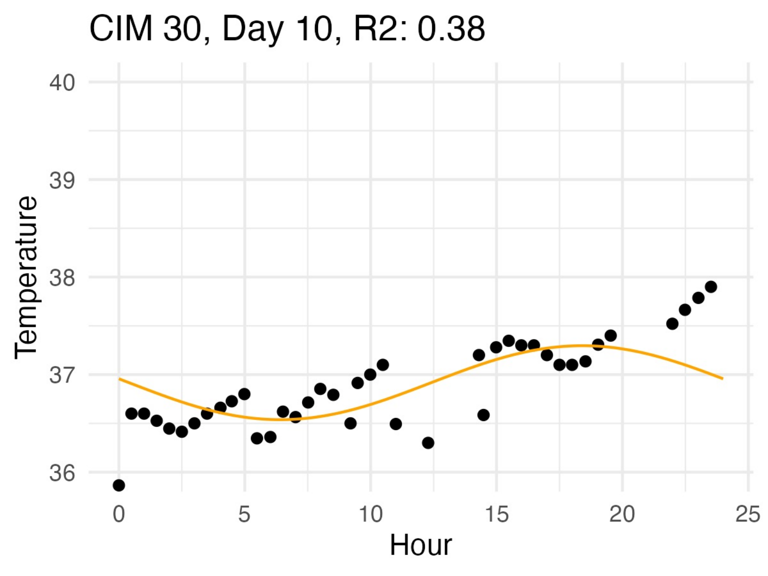
CIM 30, Day 10, R2: 0.38
Temperature
40
39
38
37
36
0
5
10
15
20
25
Hour

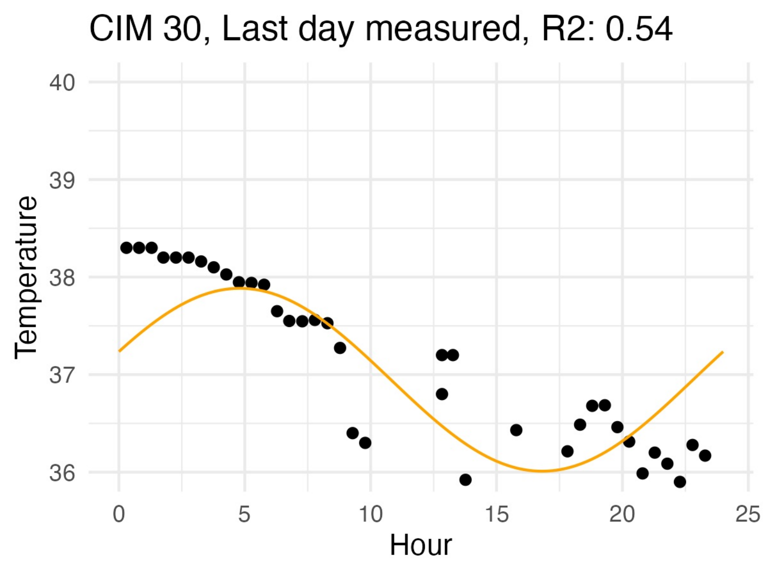

CIM 30, Last day measured, R2: 0.54
Temperature
40
39
38
37
36
Hour
0
5
10
15
20
25

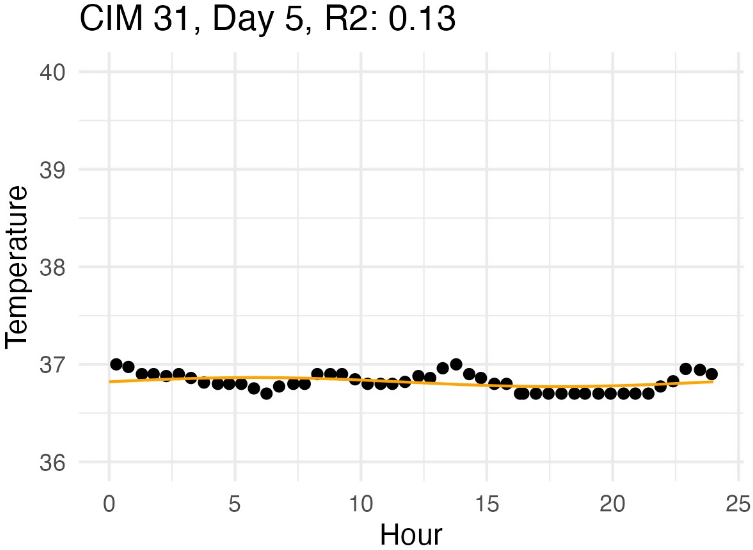

CIM 31, Day 5, R2: 0.13
Temperature
40
39
38
37
36
0
5
10
15
20
25
Hour

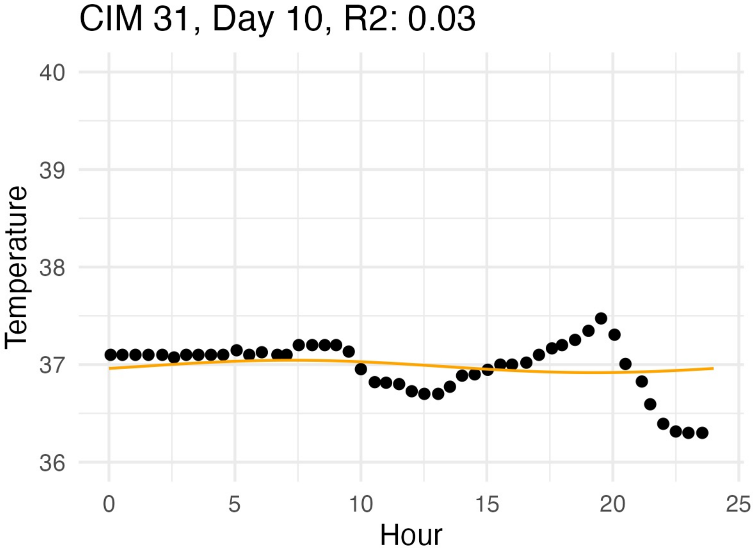
CIM 31, Day 10, R2: 0.03
Temperature
40
39
38
37
36
0
5
10
15
20
25
Hour

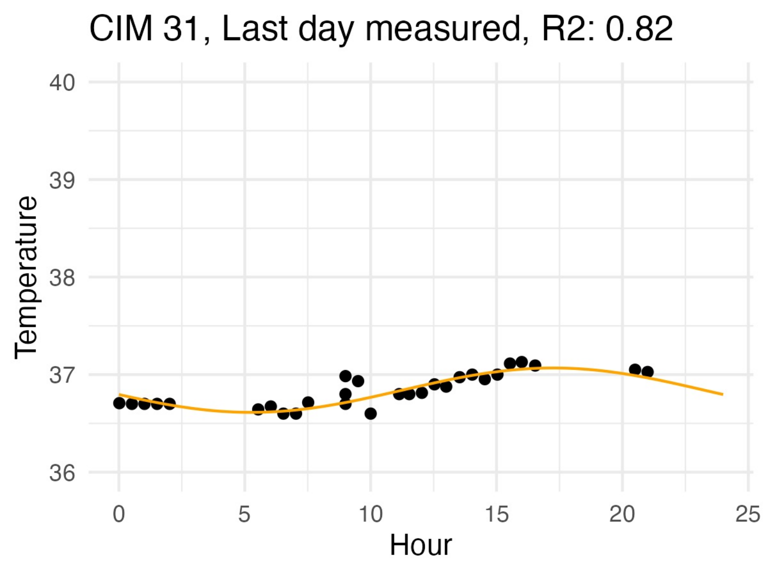
CIM 31, Last day measured, R2: 0.82
Temperature
Hour
40
39
38
37
36
0
5
10
15
20
25

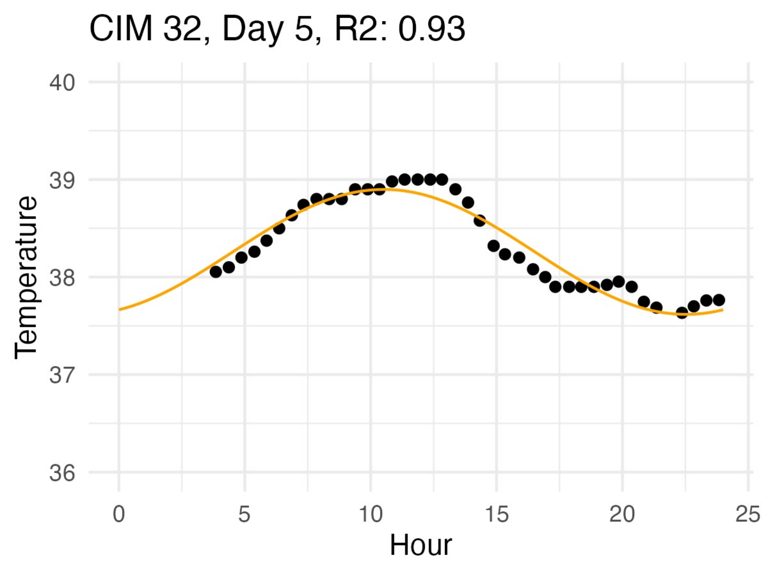
CIM 32, Day 5, R2: 0.93
Temperature
40
39
38
37
36
Hour
0
5
10
15
20
25

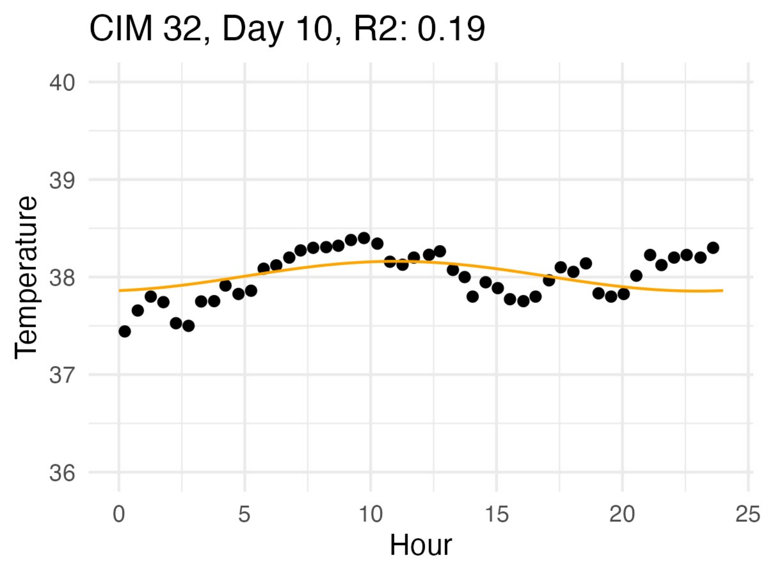
CIM 32, Day 10, R2: 0.19
Temperature
40
39
38
37
36
0
5
10
15
20
25
Hour

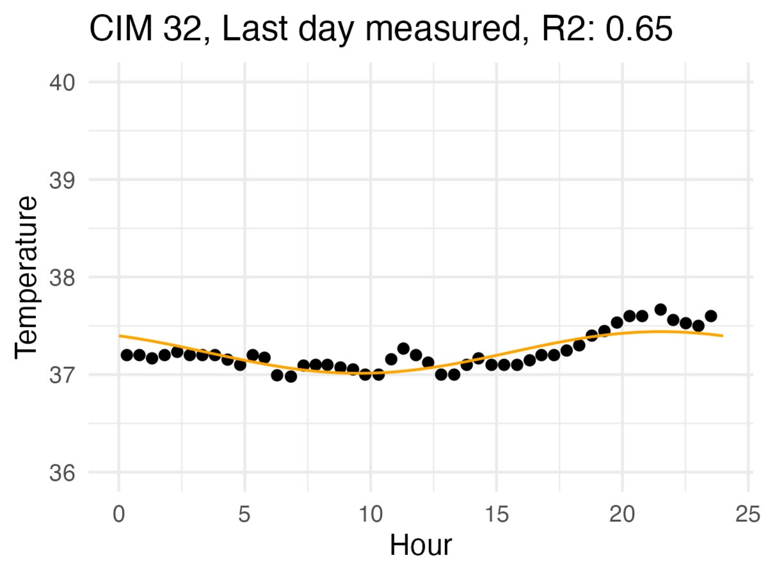
CIM 32, Last day measured, R2: 0.65
Temperature
40
39
38
37
36
Hour
0
5
10
15
20
25

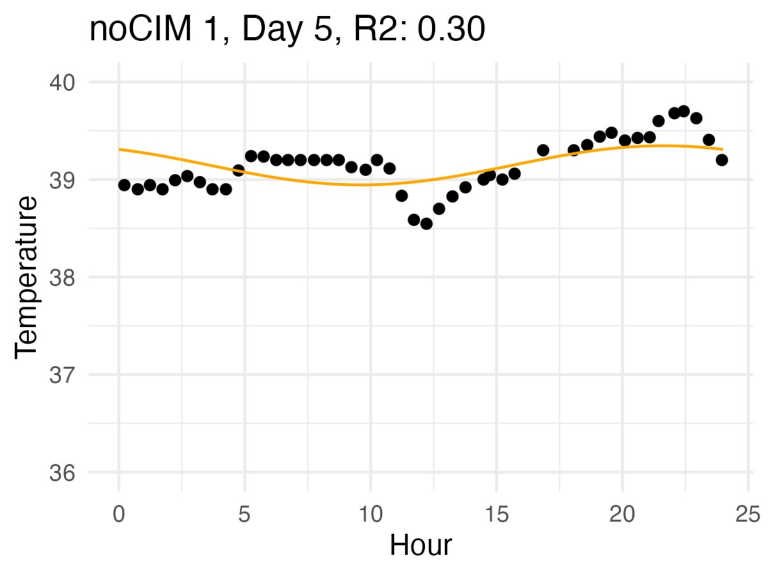

noCIM 1, Day 5, R2: 0.30
40
39
38
37
36
Temperature
0
5
10
15
20
25
Hour

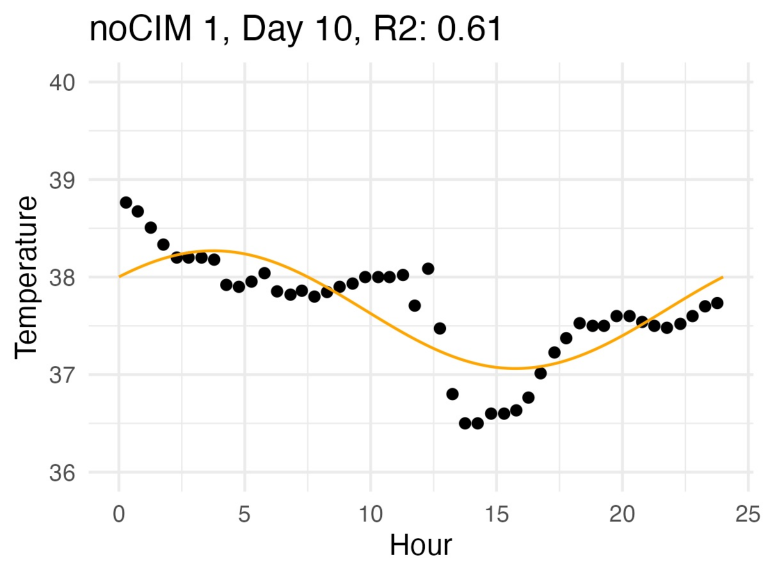
noCIM 1, Day 10, R2: 0.61
Temperature
40
39
38
37
36
0
5
10
15
20
25
Hour

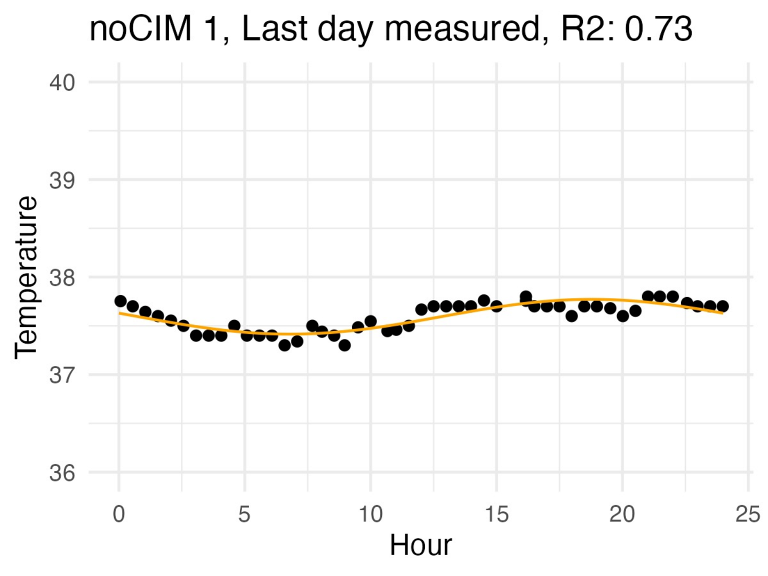

noCIM 1, Last day measured, R2: 0.73
Temperature
40
39
38
37
36
0
5
10
15
20
25
Hour

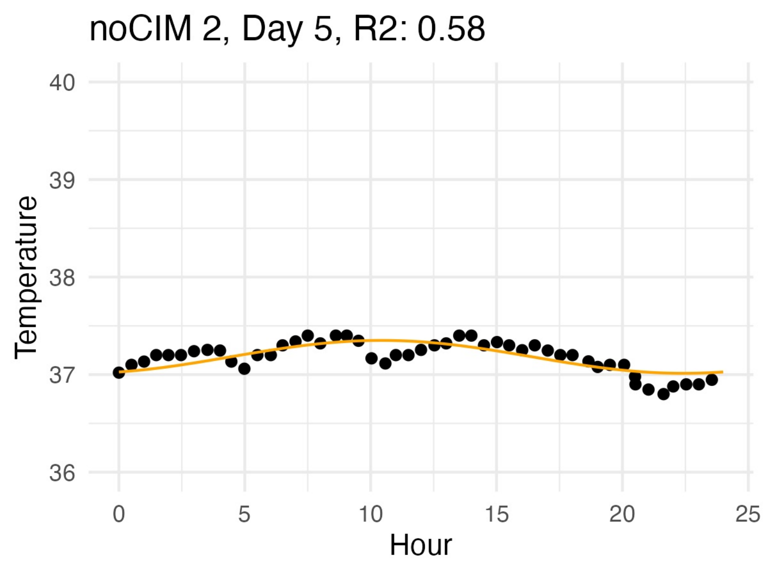
noCIM 2, Day 5, R2: 0.58
Temperature
40
39
38
37
36
0
5
10
15
20
25
Hour

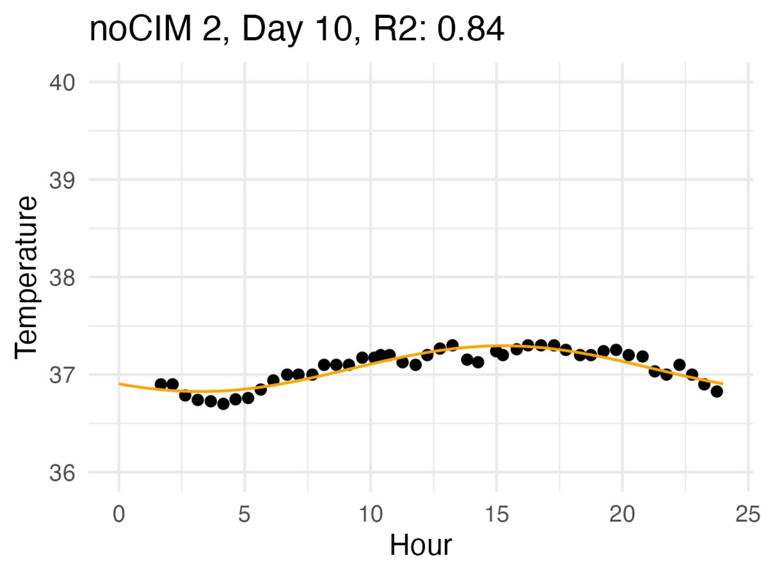
noCIM 2, Day 10, R2: 0.84
Temperature
40
39
38
37
36
Hour
0
5
10
15
20
25

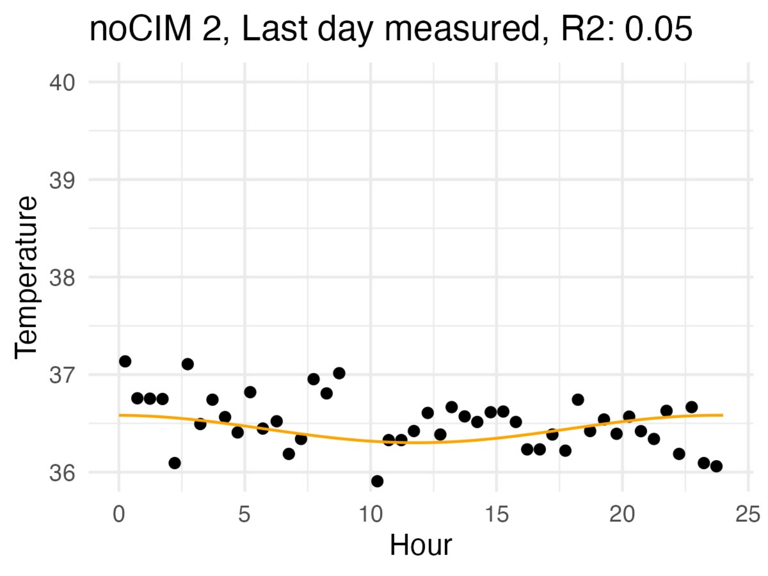

noCIM 2, Last day measured, R2: 0.05
Temperature
40
39
38
37
36
0
5
10
15
20
25
Hour

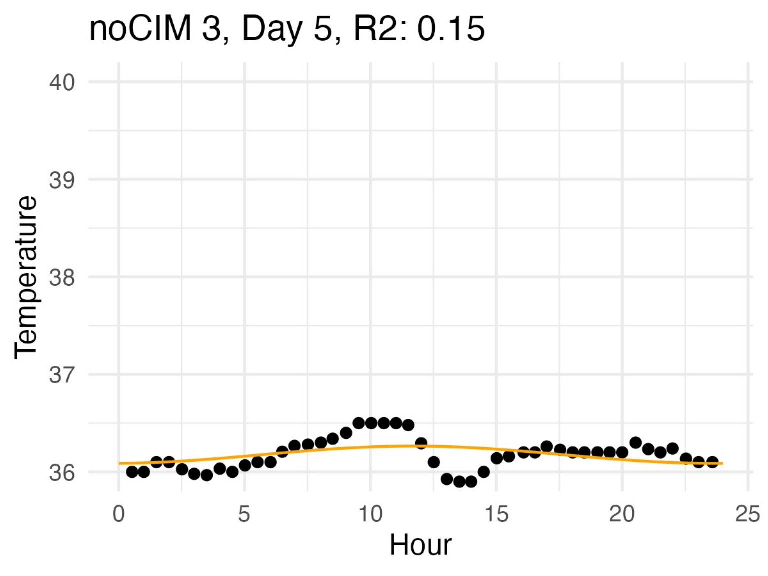
noCIM 3, Day 5, R2: 0.15
Temperature
40
39
38
37
36
0
5
10
15
20
25
Hour

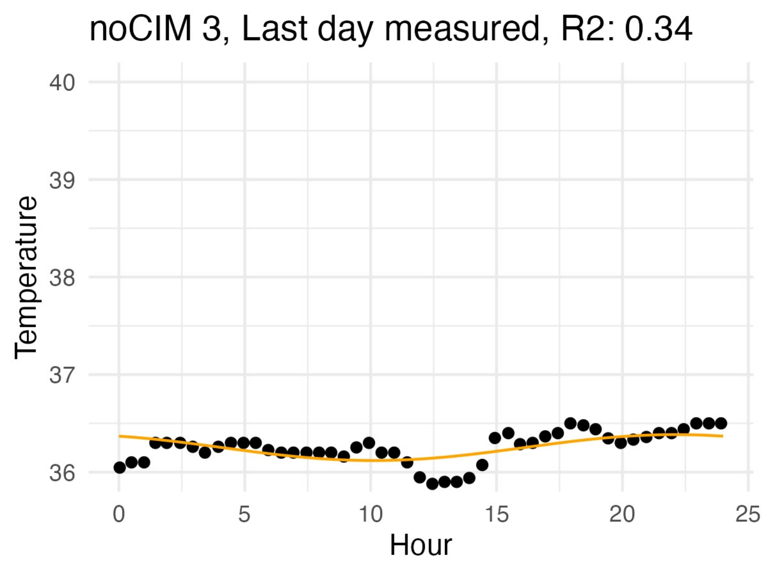

noCIM 3, Last day measured, R2: 0.34
Temperature
40
39
38
37
36
0
5
10
15
20
25
Hour

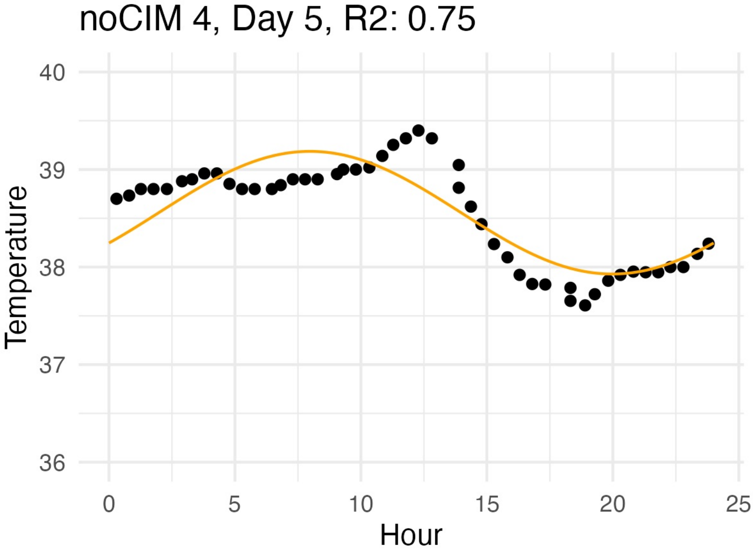

noCIM 4, Day 5, R2: 0.75
Temperature
40
39
38
37
36
0
5
10
15
20
25
Hour

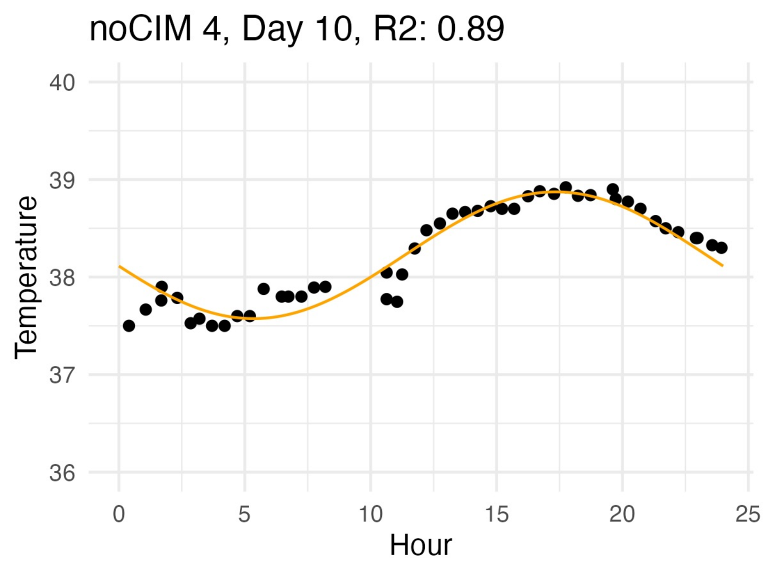

noCIM 4, Day 10, R2: 0.89
40
39
38
37
36
Temperature
0
5
10
15
20
25
Hour

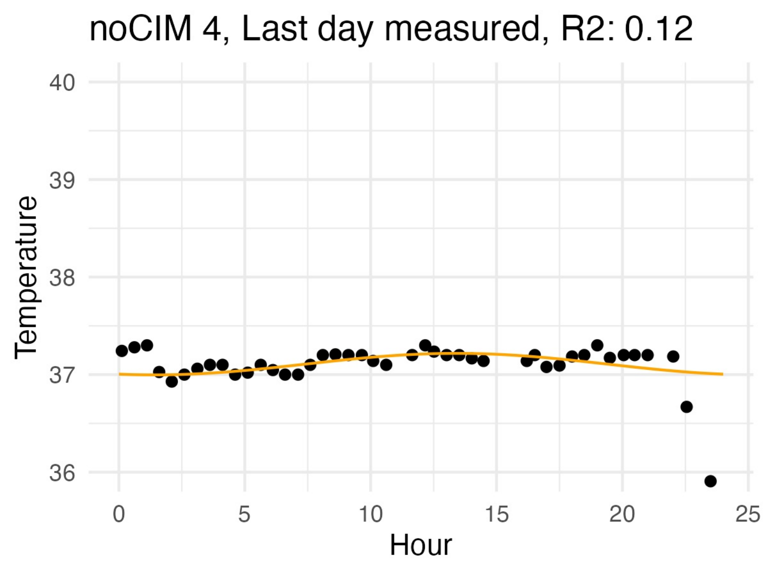
noCIM 4, Last day measured, R2: 0.12
Temperature
40
39
38
37
36
0
5
10
15
20
25
Hour

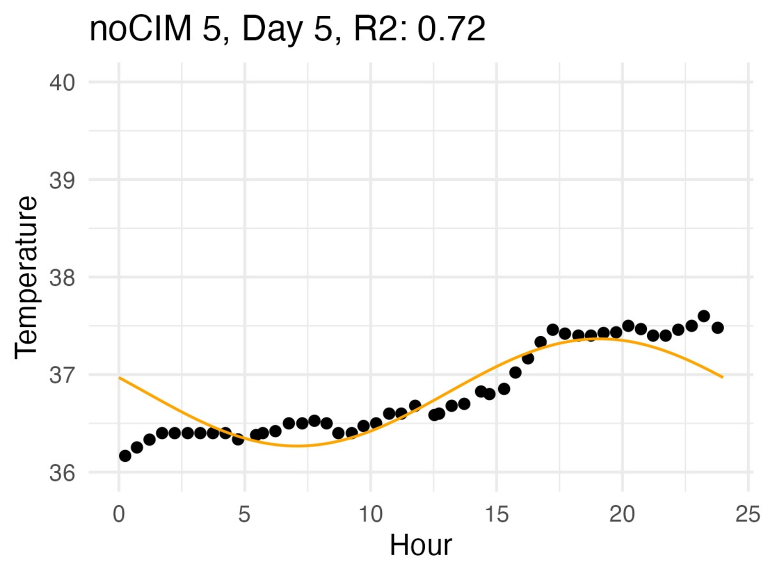

noCIM 5, Day 5, R2: 0.72
Temperature
40
39
38
37
36
Hour
0
5
10
15
20
25

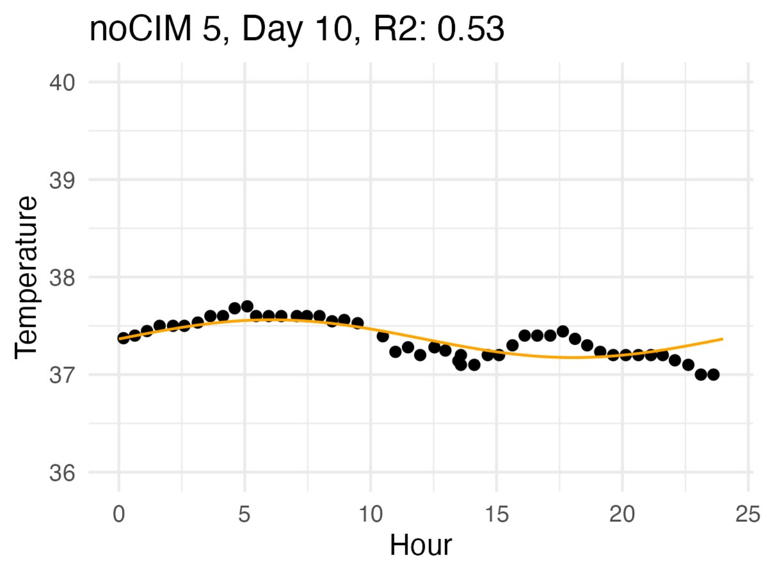
noCIM 5, Day 10, R2: 0.53
Temperature
40
39
38
37
36
0
5
10
15
20
25
Hour

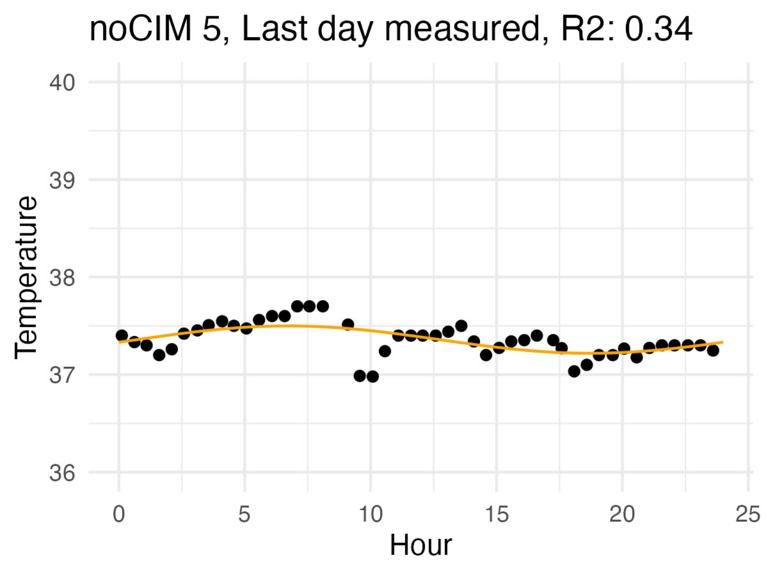
noCIM 5, Last day measured, R2: 0.34
40
39
38
37
36
Temperature
0
5
10
15
20
25
Hour

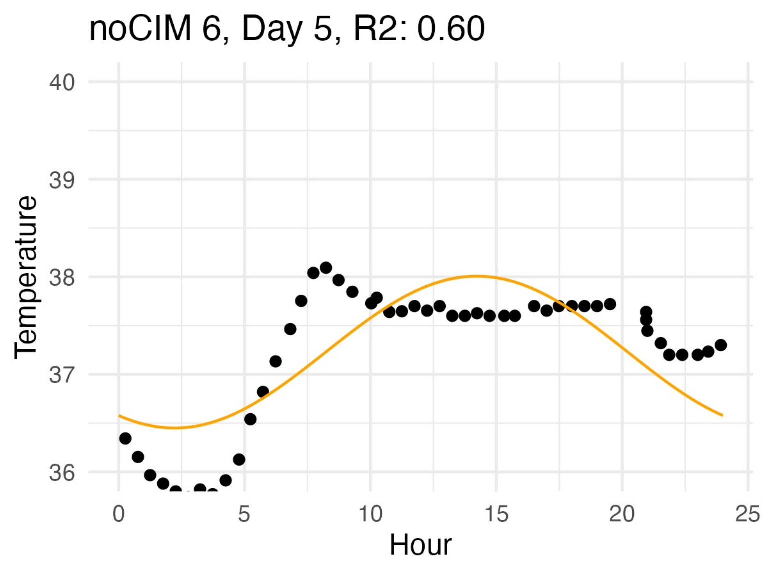
noCIM 6, Day 5, R2: 0.60
Temperature
40
39
38
37
36
0
5
10
15
20
25
Hour

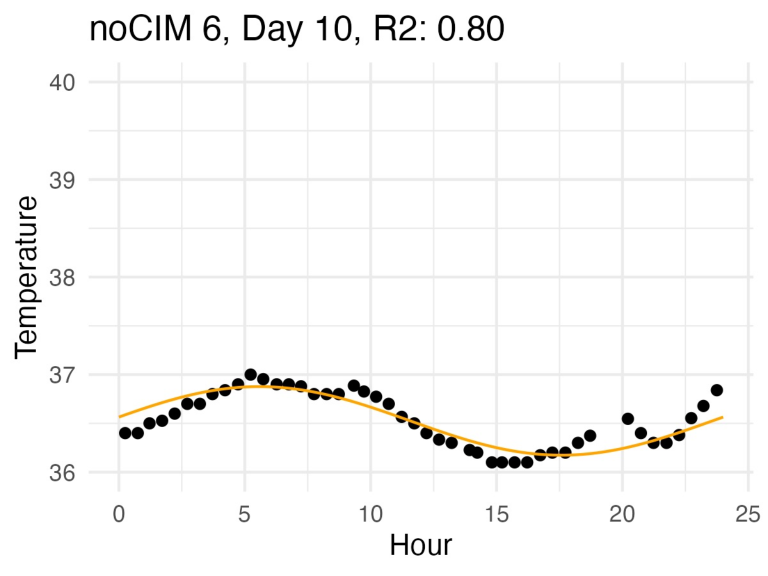

noCIM 6, Day 10, R2: 0.80
Temperature
40
39
38
37
36
0
5
10
15
20
25
Hour

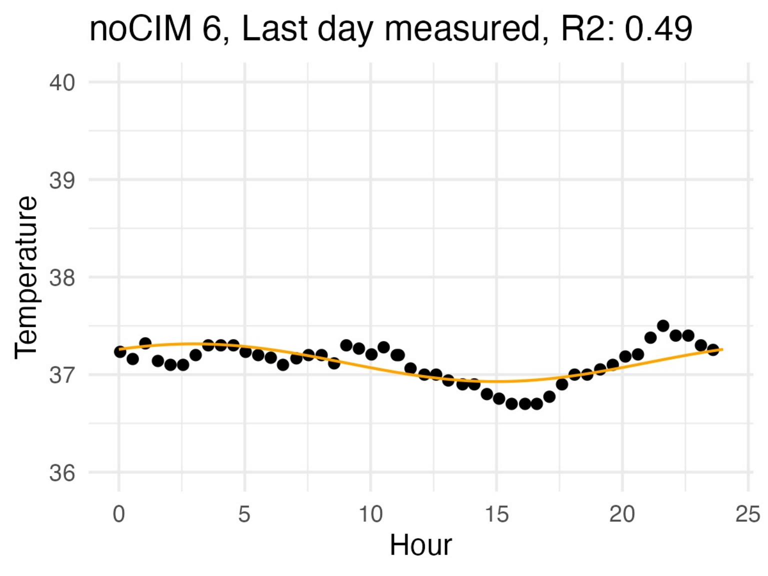
noCIM 6, Last day measured, R2: 0.49
Temperature
40
39
38
37
36
0
5
10
15
20
25
Hour

noCIM 7, Day 5, R2: 0.58

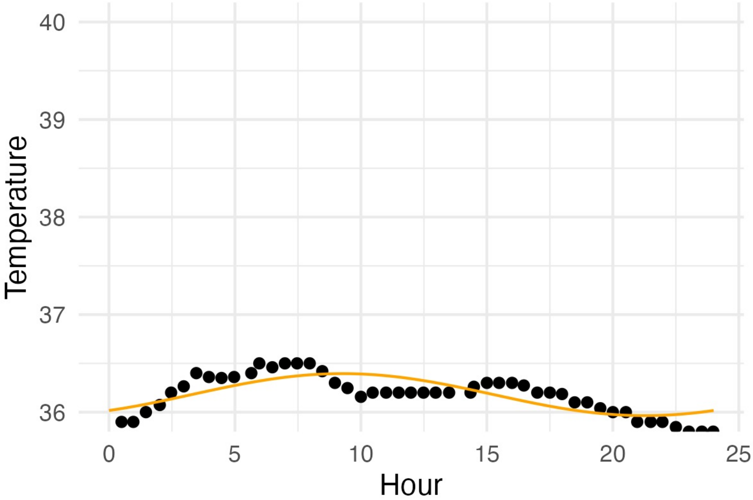

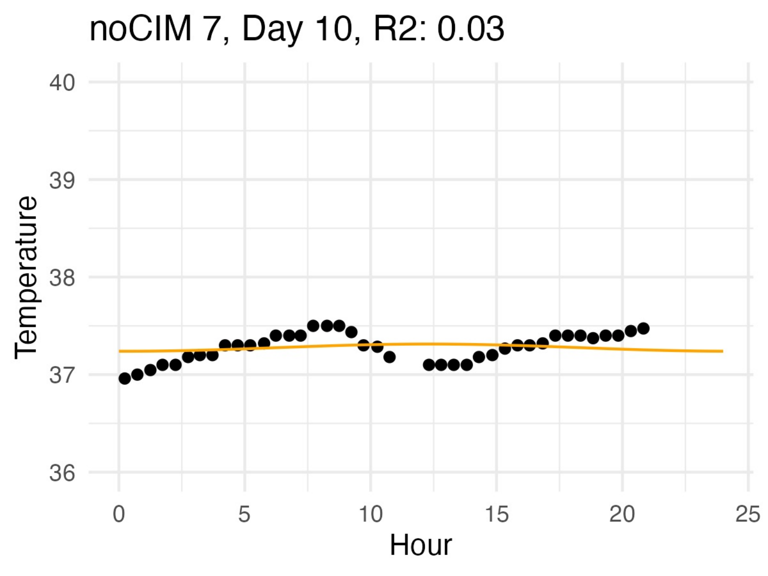

noCIM 7, Day 10, R2: 0.03
Temperature
40
39
38
37
36
0
5
10
15
20
25
Hour

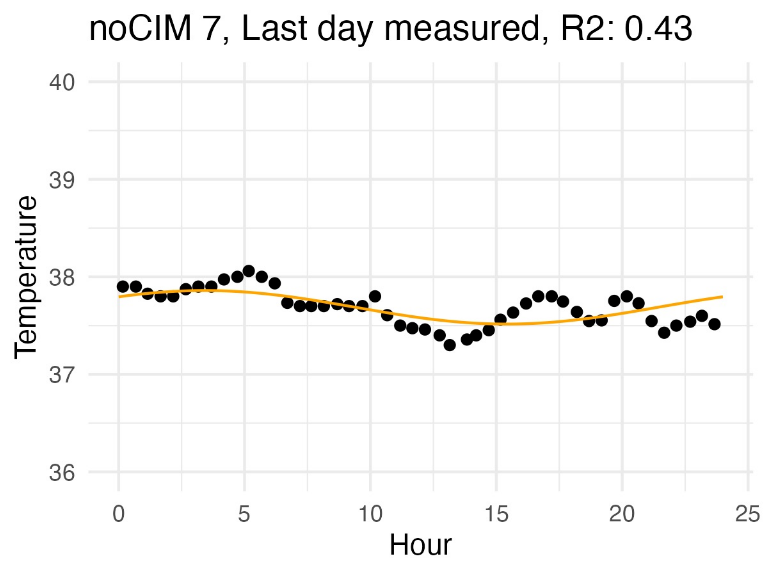

noCIM 7, Last day measured, R2: 0.43
Temperature
40
39
38
37
36
Hour
0
5
10
15
20
25

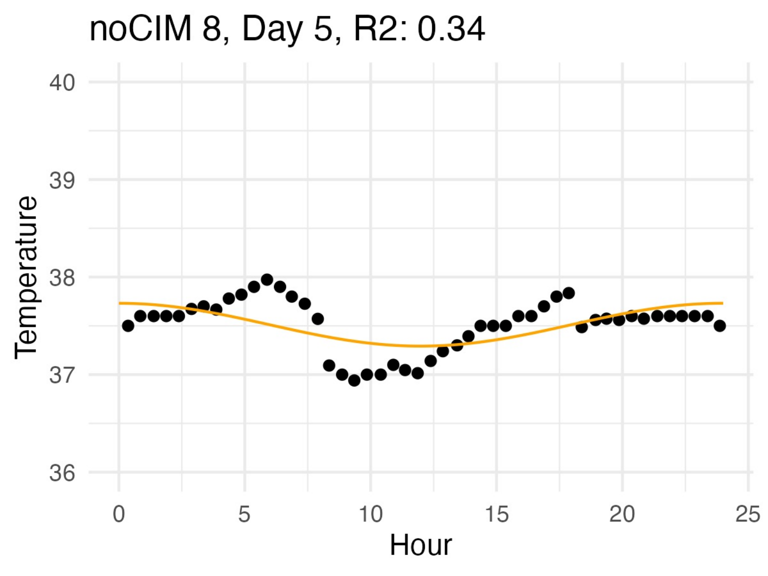
noCIM 8, Day 5, R2: 0.34
Temperature
40
39
38
37
36
Hour
0
5
10
15
20
25

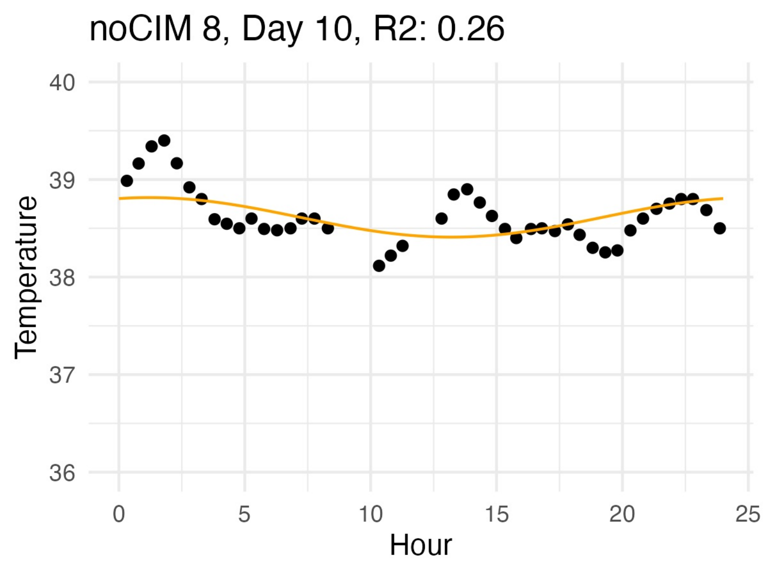
noCIM 8, Day 10, R2: 0.26
Temperature
40
39
38
37
36
Hour
0
5
10
15
20
25

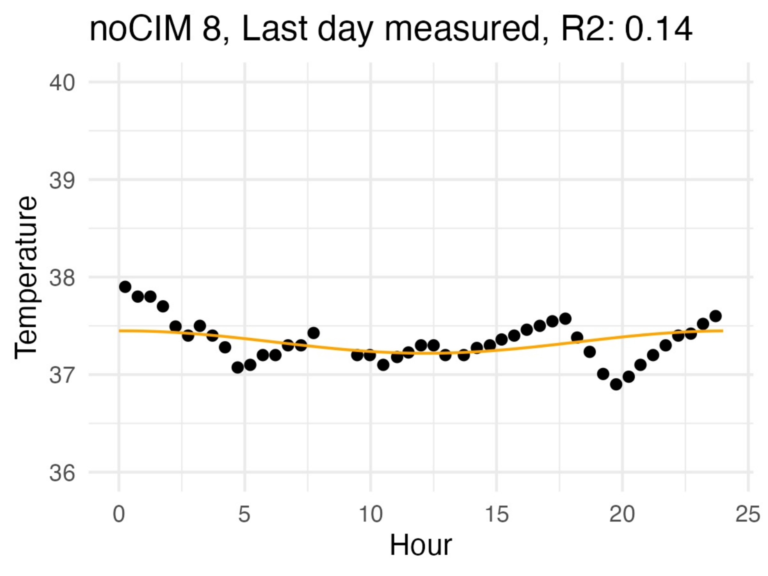
noCIM 8, Last day measured, R2: 0.14
Temperature
40
39
38
37
36
Hour
0
5
10
15
20
25

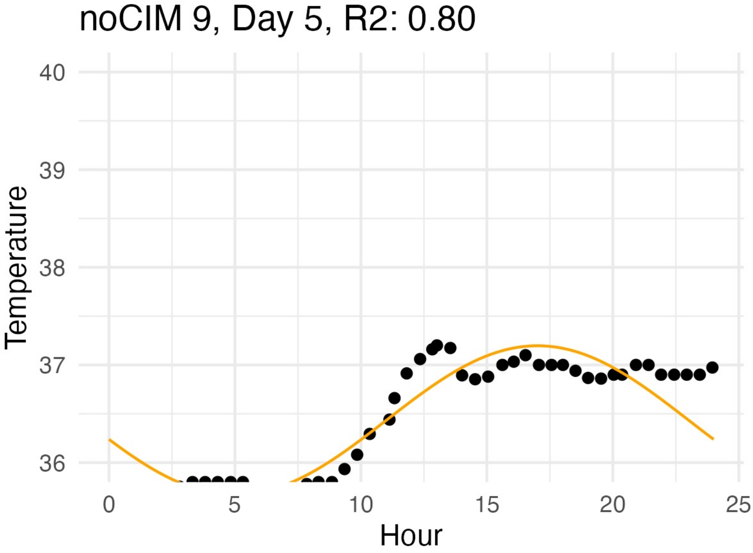
noCIM 9, Day 5, R2: 0.80
Temperature
40
39
38
37
36
Hour
0
5
10
15
20
25

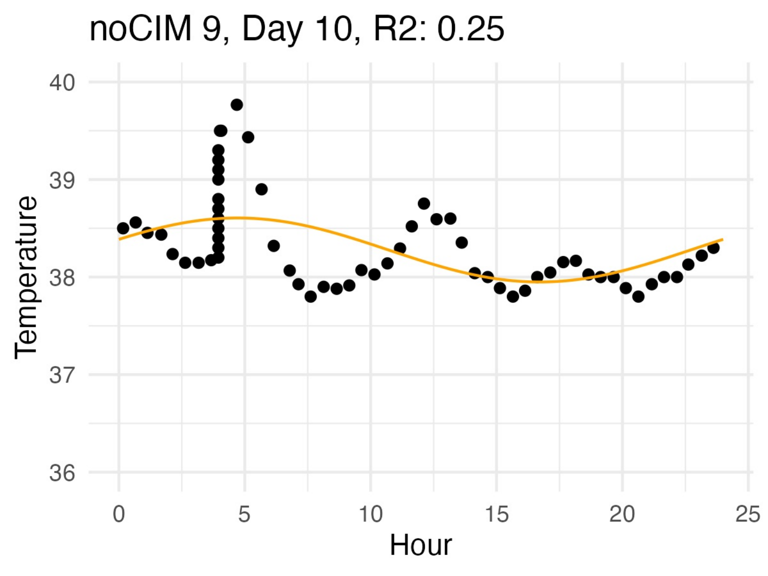
noCIM 9, Day 10, R2: 0.25
Temperature
40
39
38
37
36
0
5
10
15
20
25
Hour

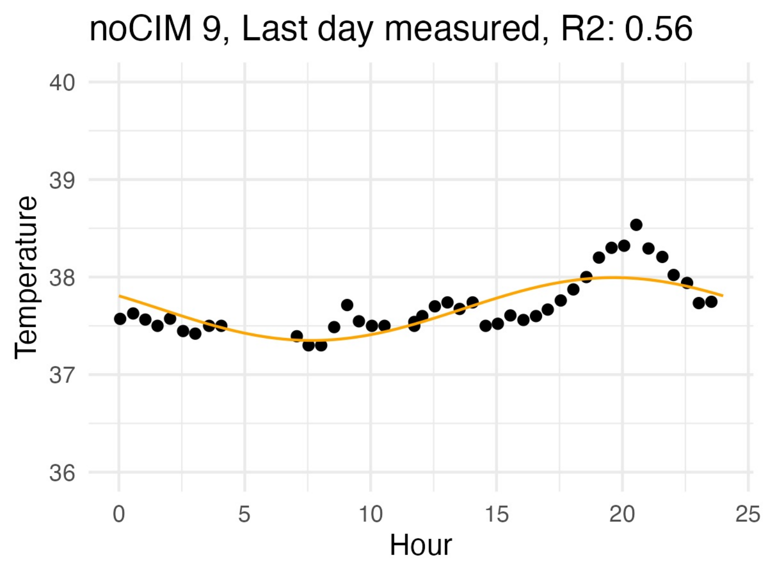
noCIM 9, Last day measured, R2: 0.56
Temperature
40
39
38
37
36
0
5
10
15
20
25
Hour

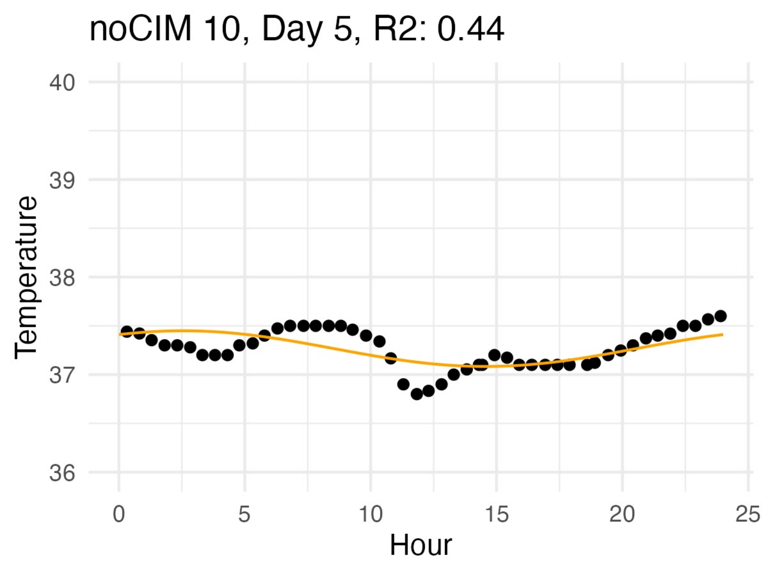

noCIM 10, Day 5, R2: 0.44
Temperature
40
39
38
37
36
Hour
0
5
10
15
20
25

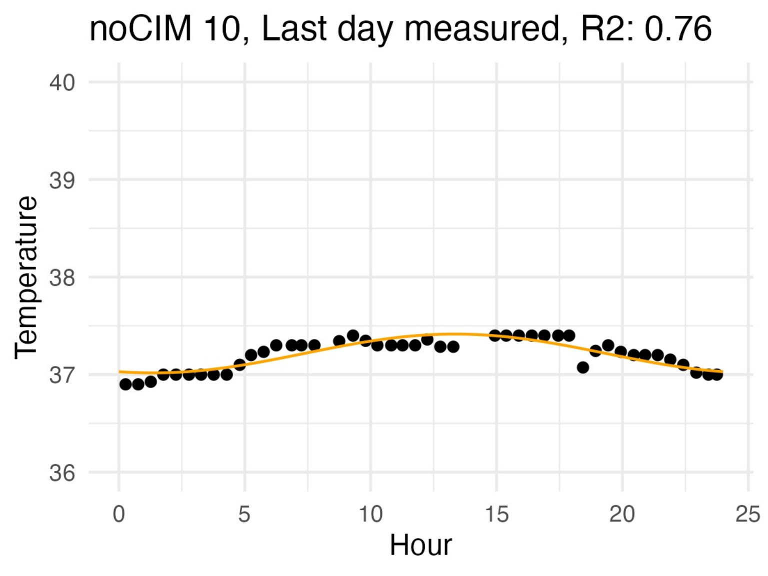

noCIM 10, Last day measured, R2: 0.76
Temperature
40
39
38
37
36
0
5
10
15
20
25
Hour

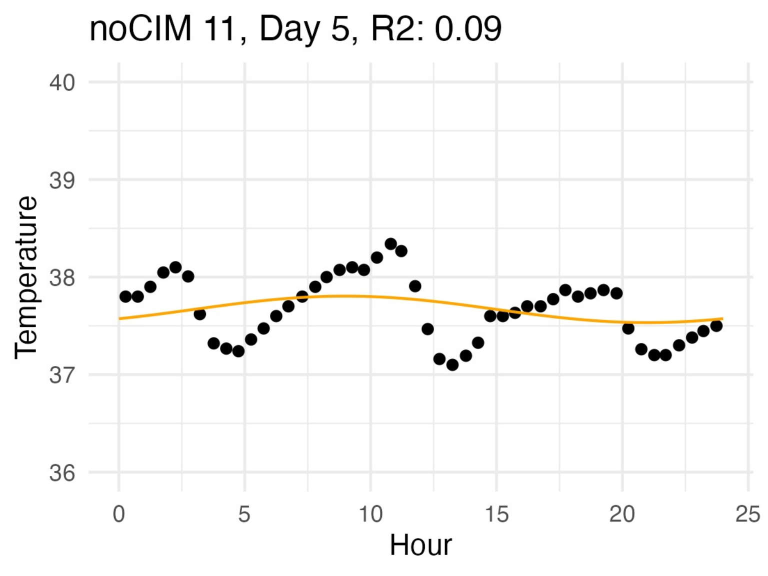
noCIM 11, Day 5, R2: 0.09
Temperature
40
39
38
37
36
Hour
0
5
10
15
20
25

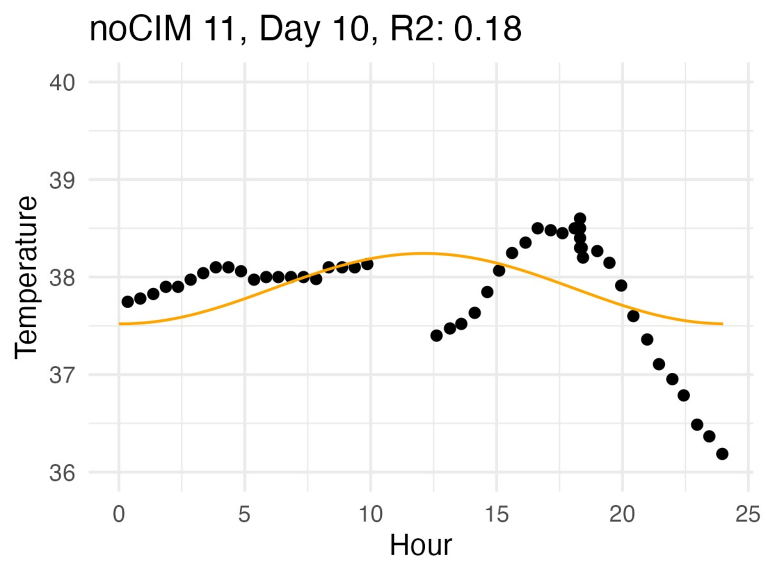
noCIM 11, Day 10, R2: 0.18
Temperature
40
39
38
37
36
0
5
10
15
20
25
Hour

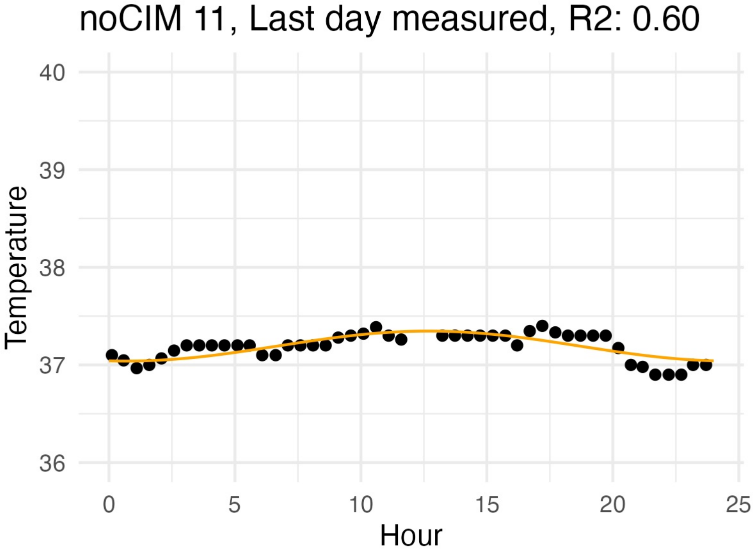
noCIM 11, Last day measured, R2: 0.60
Temperature
40
39
38
37
36
0
5
10
15
20
25
Hour

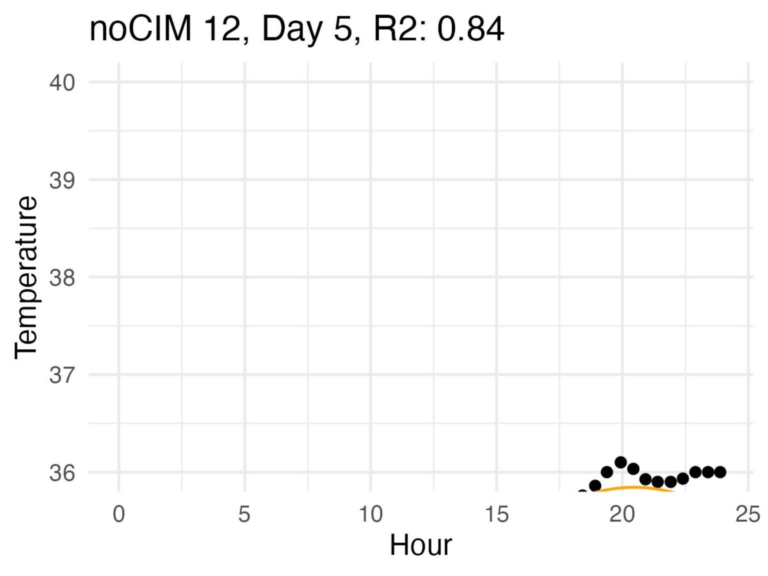

noCIM 12, Day 5, R2: 0.84
40
39
38
37
36
Temperature
0
5
10
15
20
25
Hour

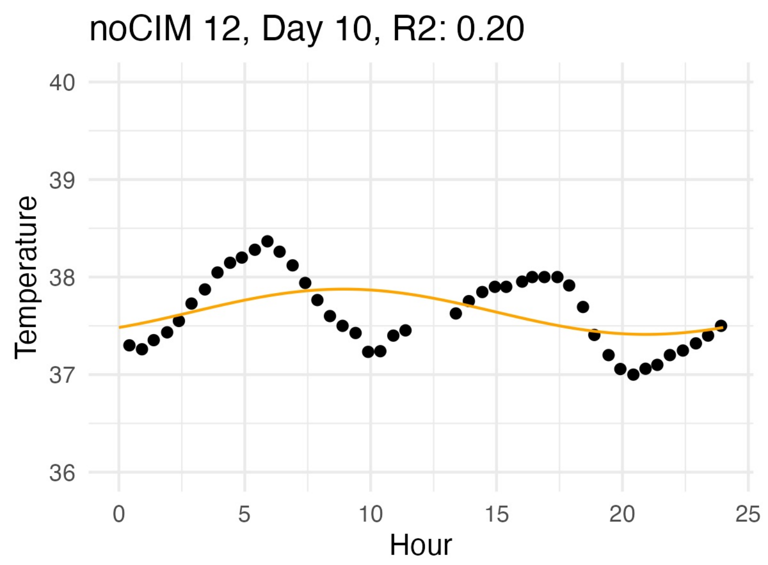

noCIM 12, Day 10, R2: 0.20
Temperature
40
39
38
37
36
0
5
10
15
20
25
Hour

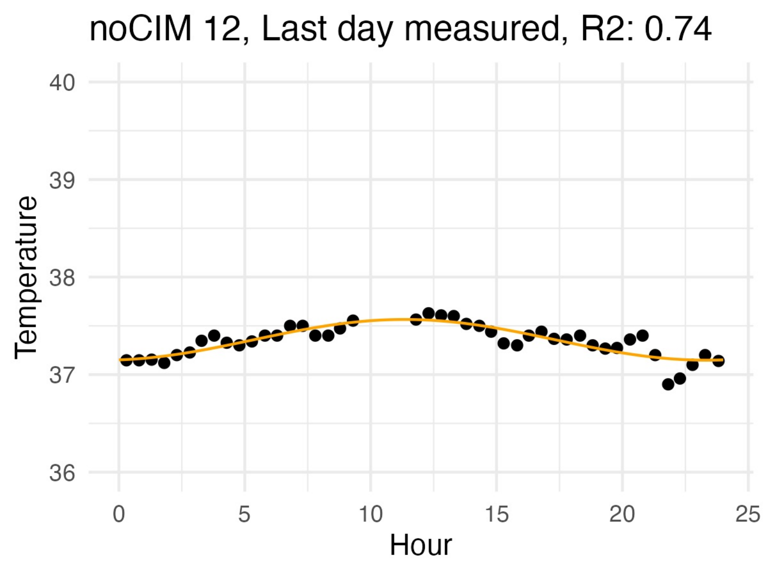
noCIM 12, Last day measured, R2: 0.74
Temperature
40
39
38
37
36
Hour
0
5
10
15
20
25

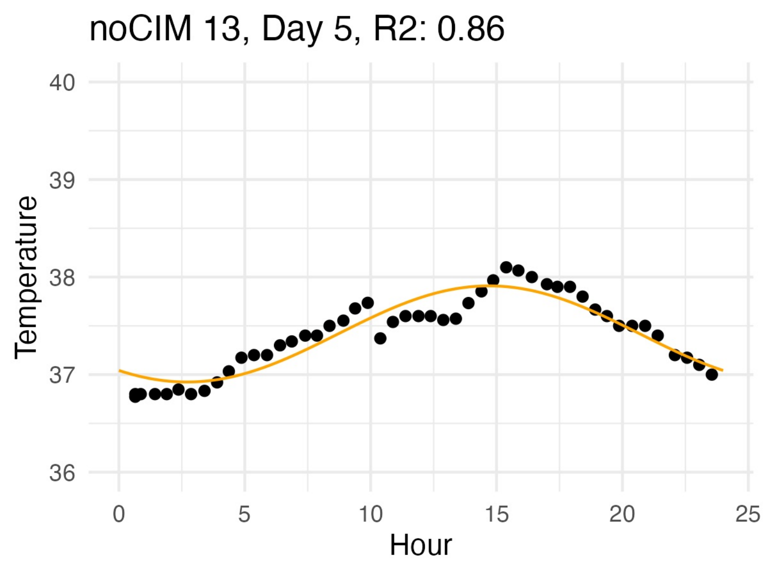

noCIM 13, Day 5, R2: 0.86
Temperature
40
39
38
37
36
0
5
10
15
20
25
Hour

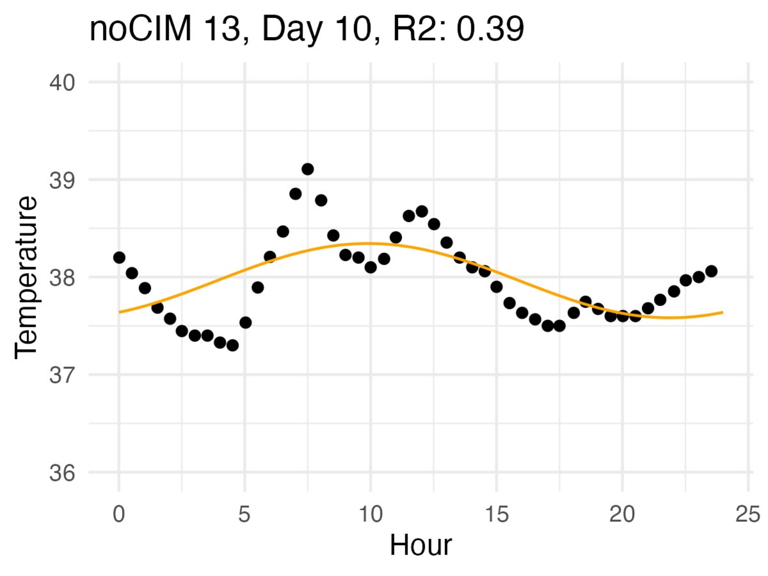
noCIM 13, Day 10, R2: 0.39
Temperature
40
39
38
37
36
0
5
10
15
20
25
Hour

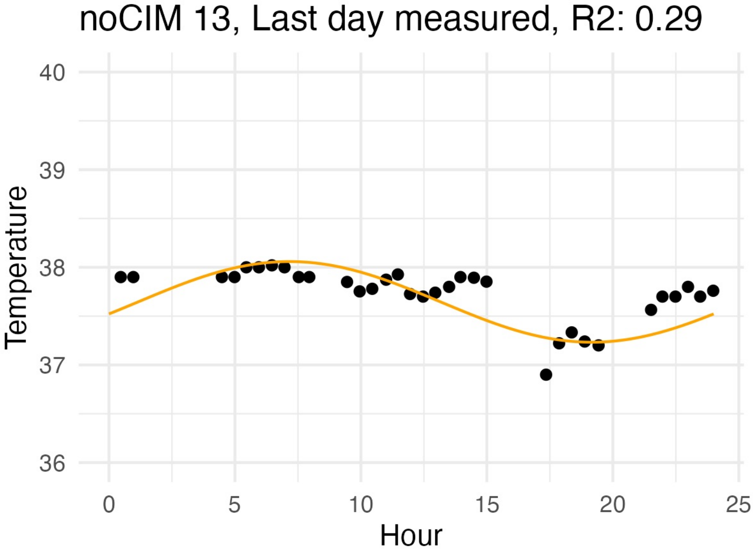
noCIM 13, Last day measured, R2: 0.29
Temperature
40
39
38
37
36
0
5
10
15
20
25
Hour

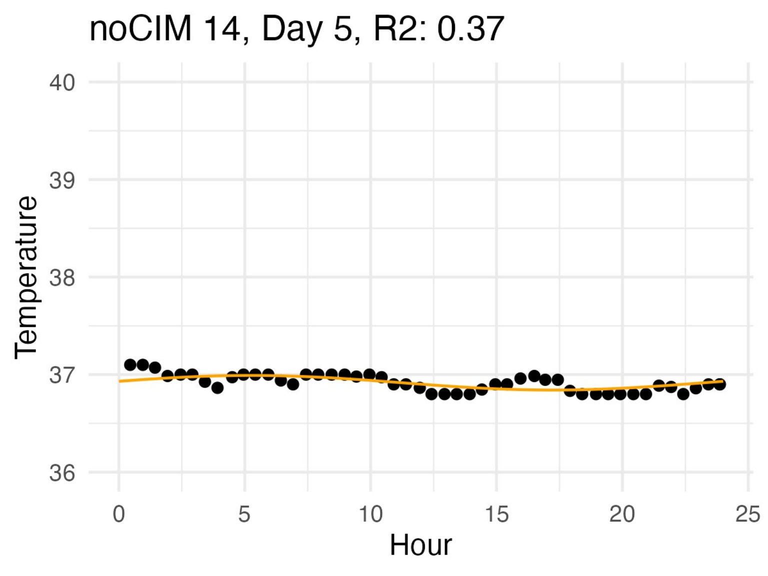
noCIM 14, Day 5, R2: 0.37
Temperature
40
39
38
37
36
0
5
10
15
20
25
Hour

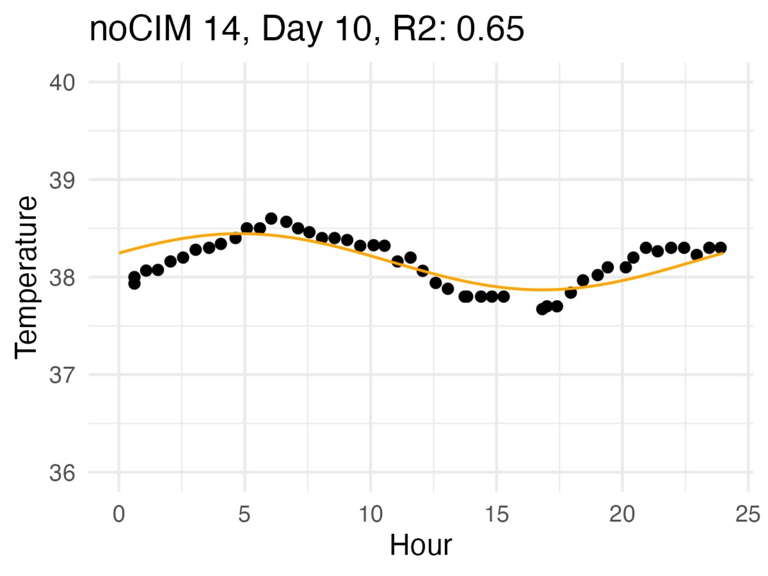

noCIM 14, Day 10, R2: 0.65
Temperature
40
39
38
37
36
Hour
0
5
10
15
20
25

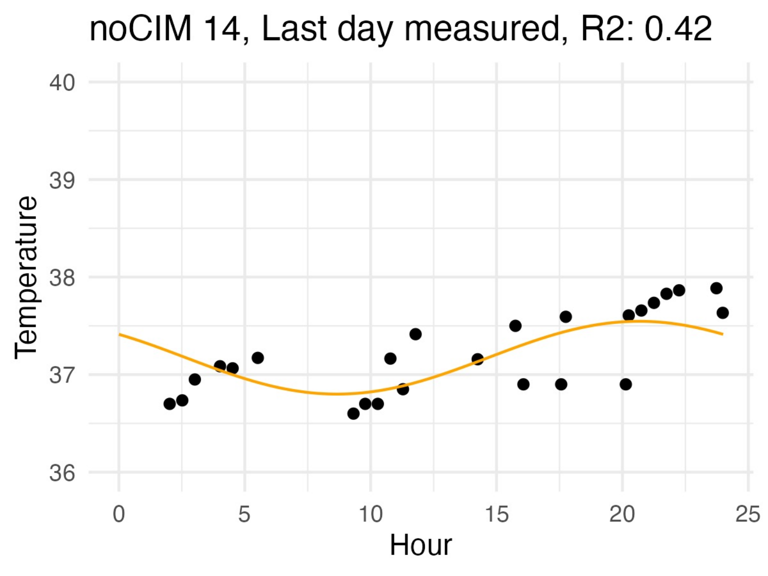

noCIM 14, Last day measured, R2: 0.42
Temperature
36
37
38
39
40
Hour
0
5
10
15
20
25

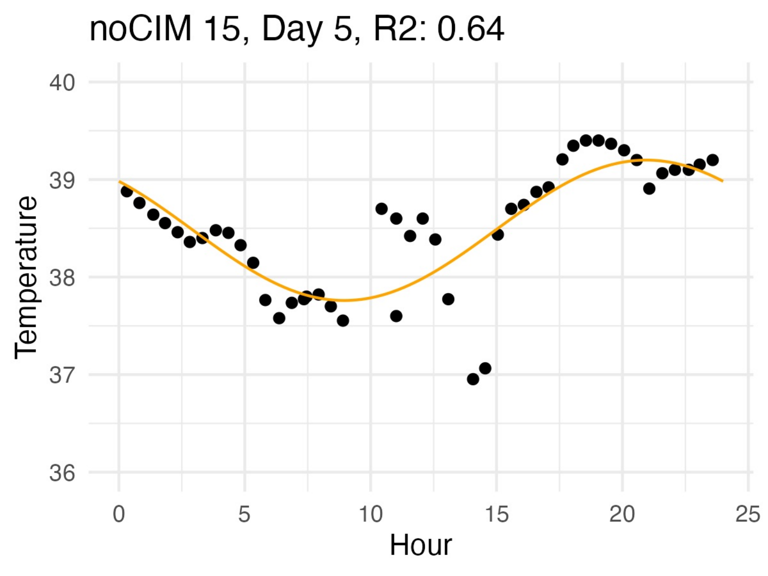
noCIM 15, Day 5, R2: 0.64
Temperature
40
39
38
37
36
0
5
10
15
20
25
Hour

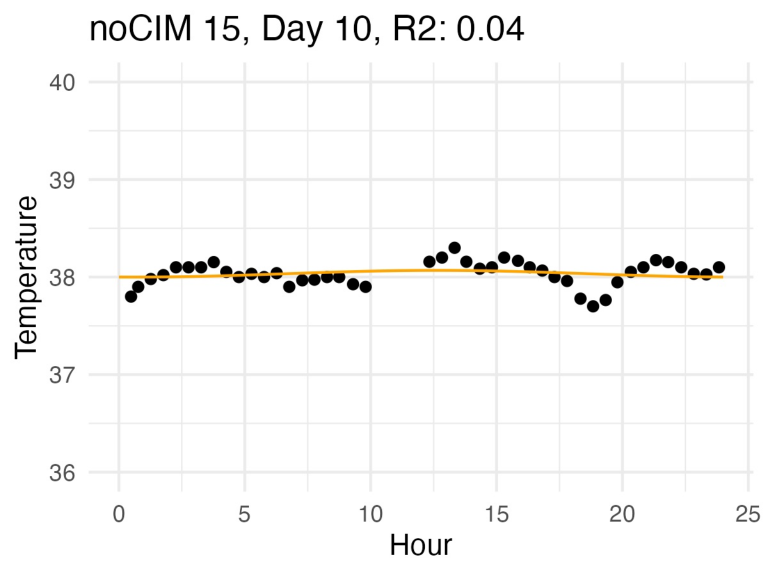

noCIM 15, Day 10, R2: 0.04
40
39
38
37
36
Temperature
0
5
10
15
20
25
Hour

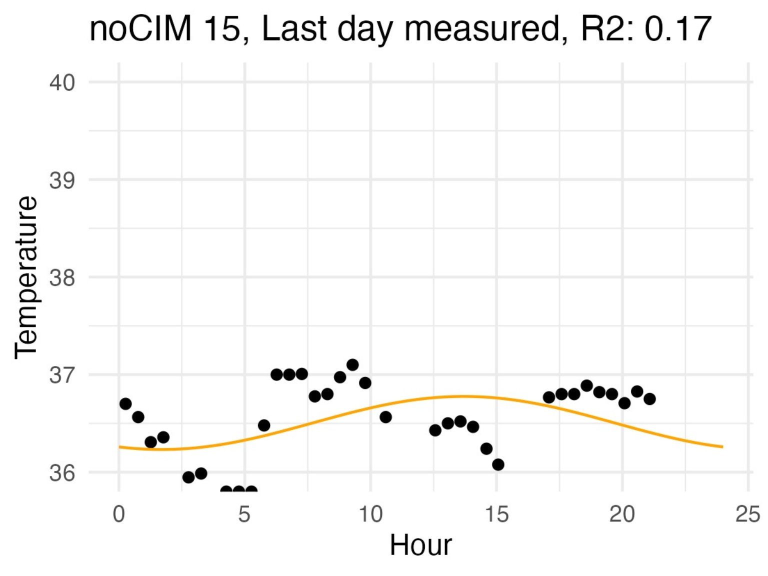

noCIM 15, Last day measured, R2: 0.17
Temperature
40
39
38
37
36
Hour
0
5
10
15
20
25

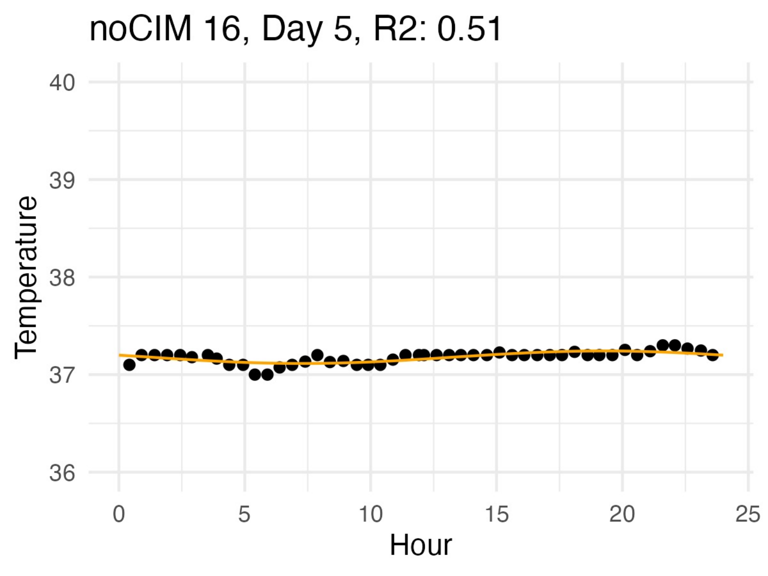

noCIM 16, Day 5, R2: 0.51
40
39
38
37
36
Temperature
0
5
10
15
20
25
Hour

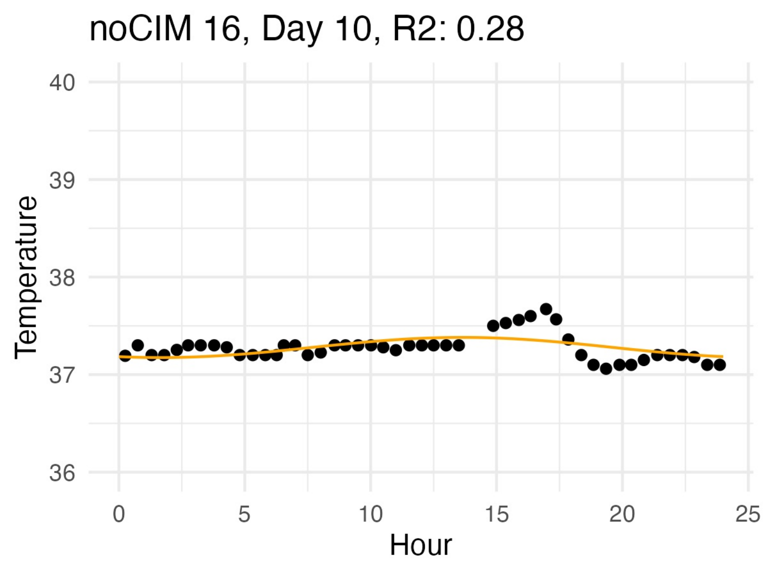

noCIM 16, Day 10, R2: 0.28
Temperature
40
39
38
37
36
0
5
10
15
20
25
Hour

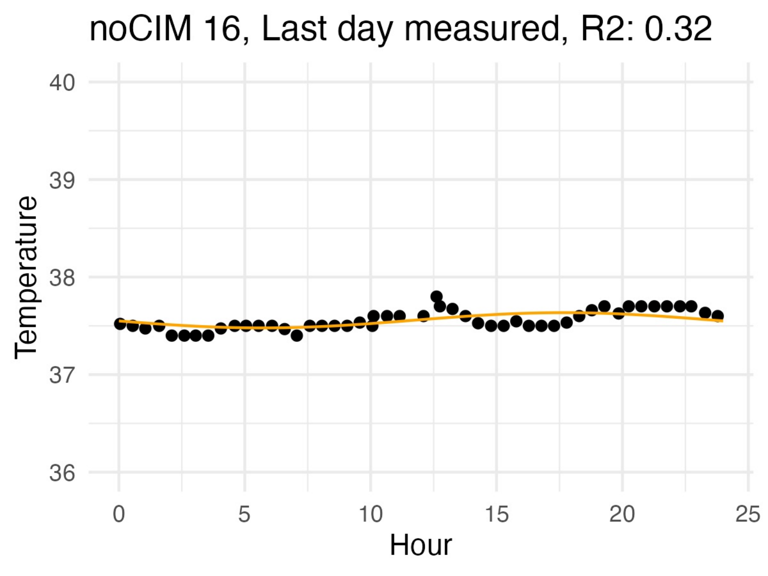
noCIM 16, Last day measured, R2: 0.32
Temperature
40
39
38
37
36
0
5
10
15
20
25
Hour

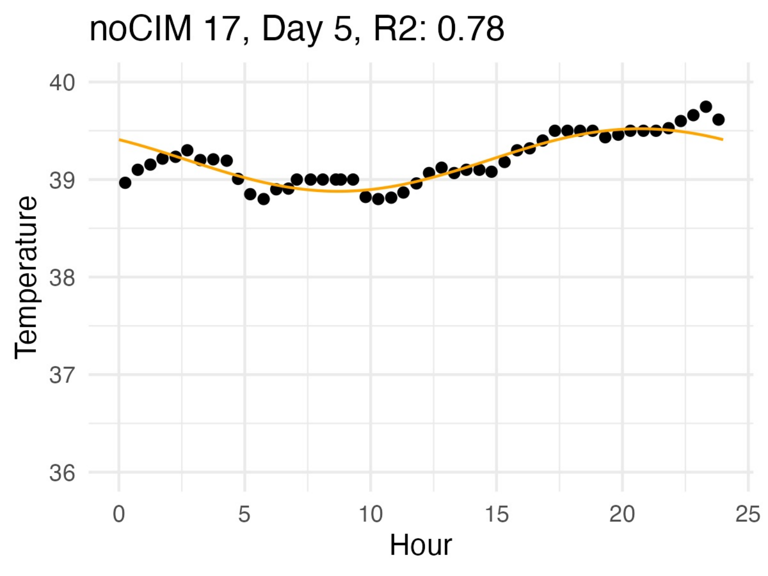

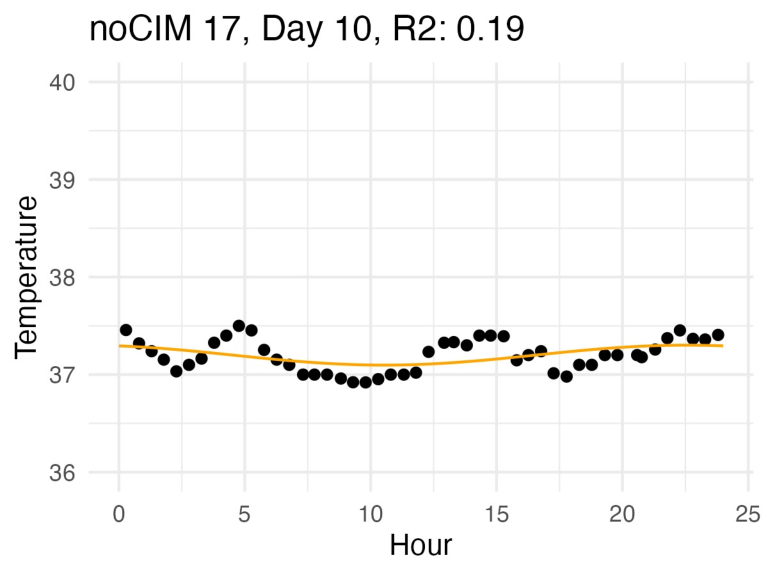
noCIM 17, Day 10, R2: 0.19
Temperature
40
39
38
37
36
Hour
0
5
10
15
20
25

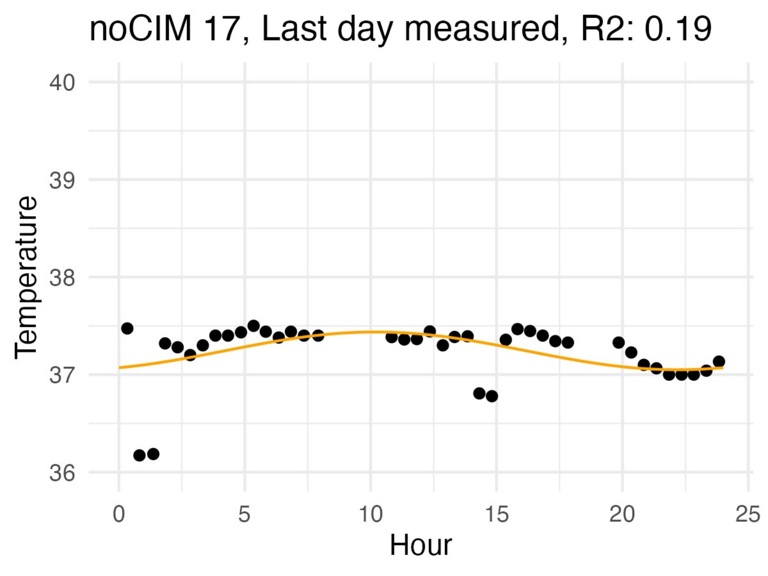

noCIM 17, Last day measured, R2: 0.19
Temperature
40
39
38
37
36
0
5
10
15
20
25
Hour

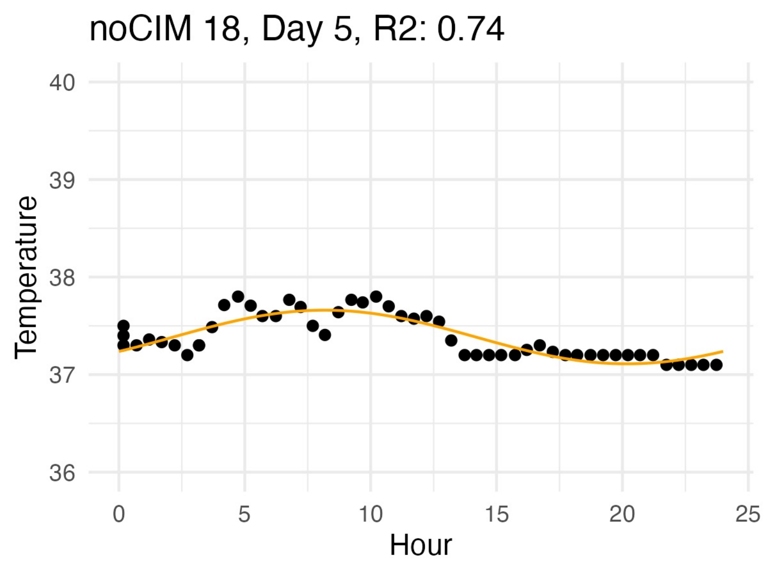

noCIM 18, Day 5, R2: 0.74
Temperature
40
39
38
37
36
Hour
0
5
10
15
20
25

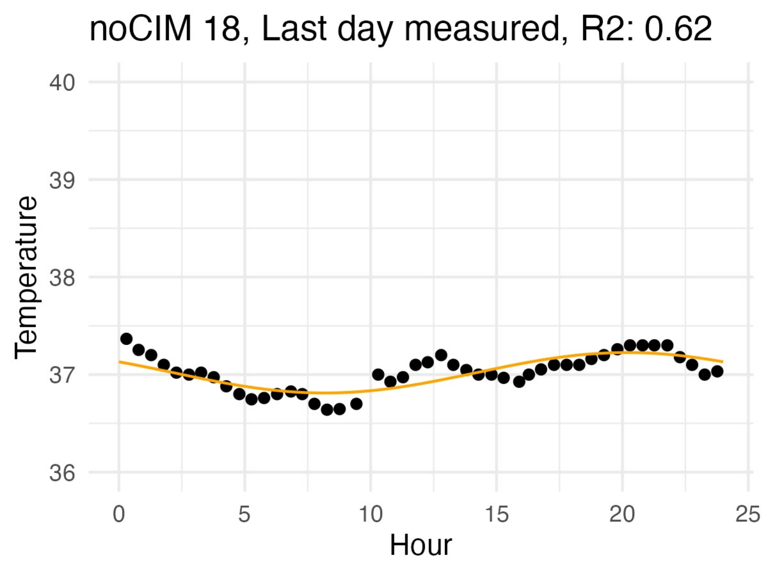
noCIM 18, Last day measured, R2: 0.62
Temperature
40
39
38
37
36
Hour
0
5
10
15
20
25

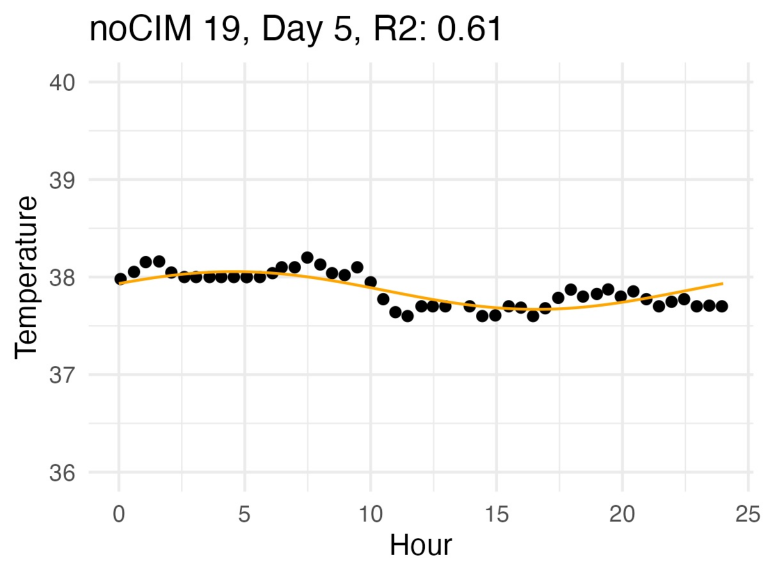
noCIM 19, Day 5, R2: 0.61
40
39
38
37
36
Temperature
0
5
10
15
20
25
Hour

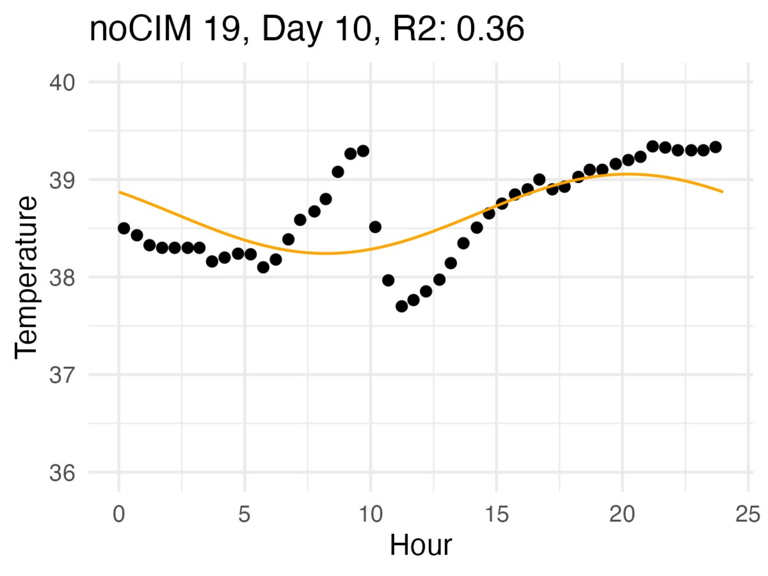
noCIM 19, Day 10, R2: 0.36
Temperature
40
39
38
37
36
Hour
0
5
10
15
20
25

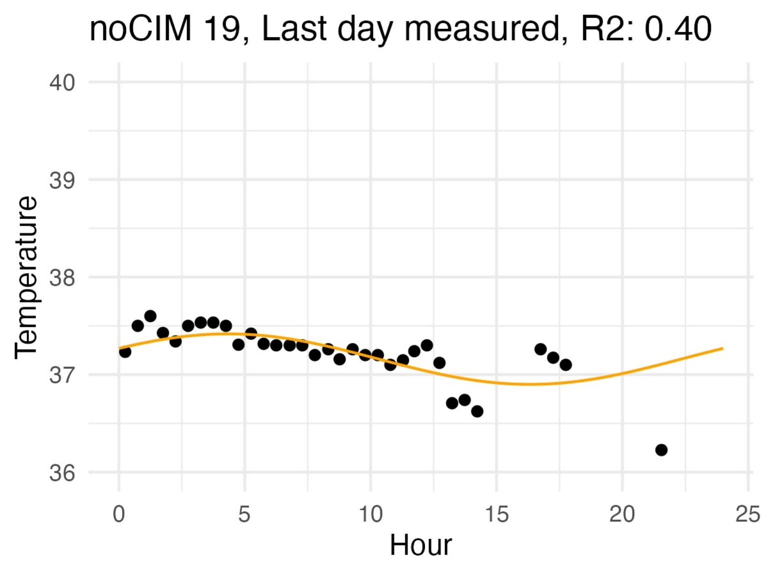
noCIM 19, Last day measured, R2: 0.40
Temperature
40
39
38
37
36
0
5
10
15
20
25
Hour

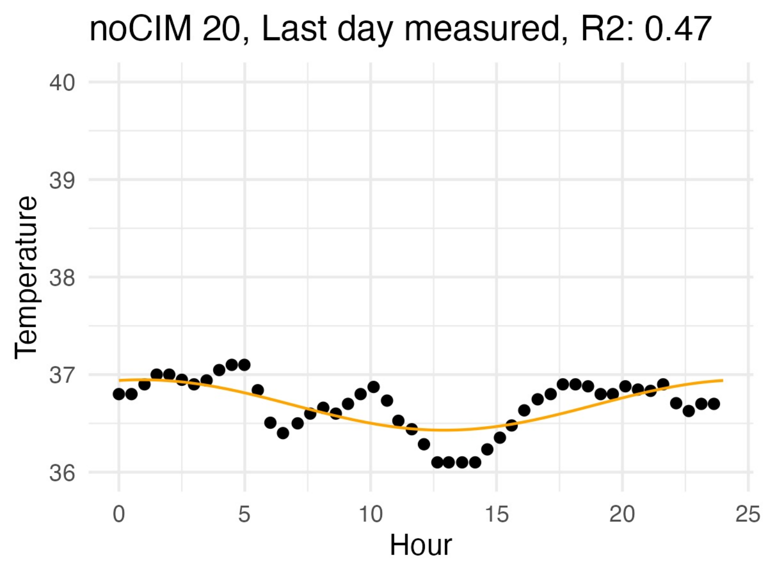
noCIM 20, Last day measured, R2: 0.47
Temperature
40
39
38
37
36
0
5
10
15
20
25
Hour

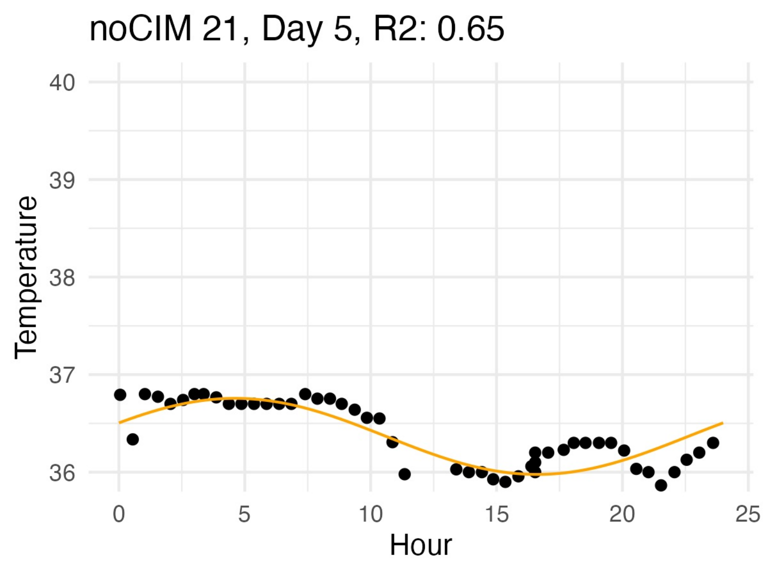

noCIM 21, Day 5, R2: 0.65
40
39
38
37
36
Temperature
0
5
10
15
20
25
Hour

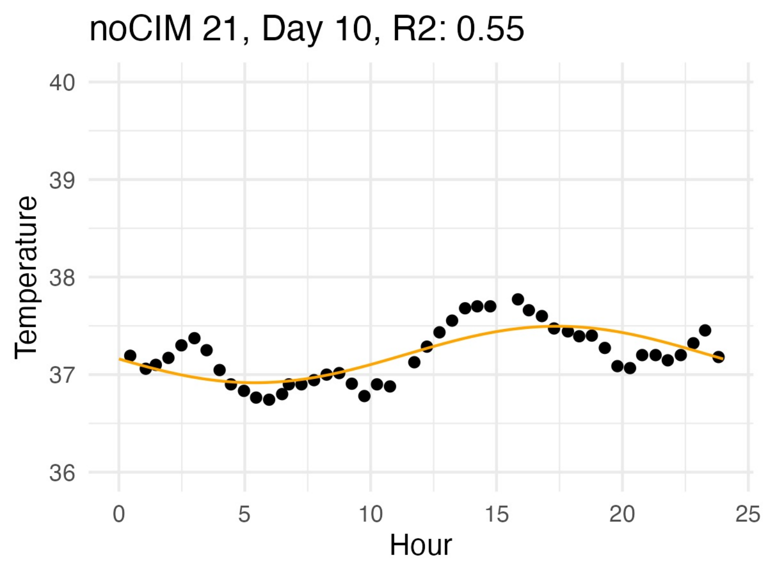
noCIM 21, Day 10, R2: 0.55
Temperature
40
39
38
37
36
0
5
10
15
20
25
Hour

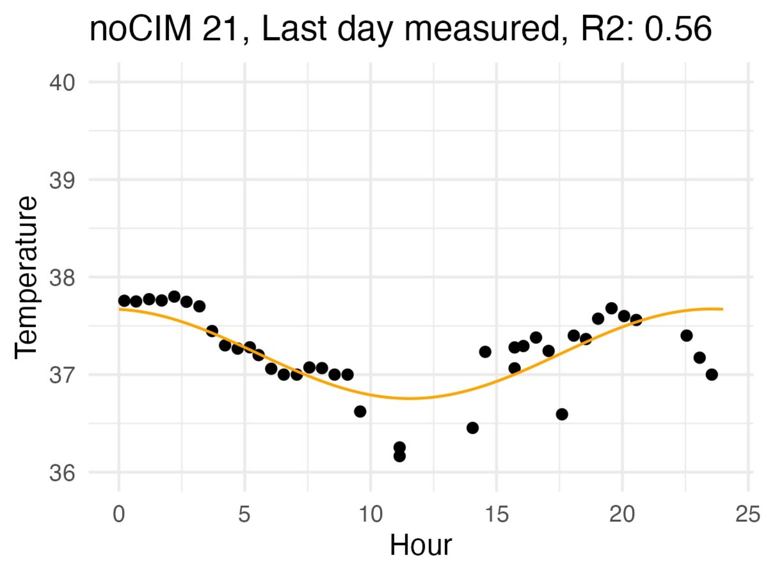
noCIM 21, Last day measured, R2: 0.56
Temperature
40
39
38
37
36
0
5
10
15
20
25
Hour

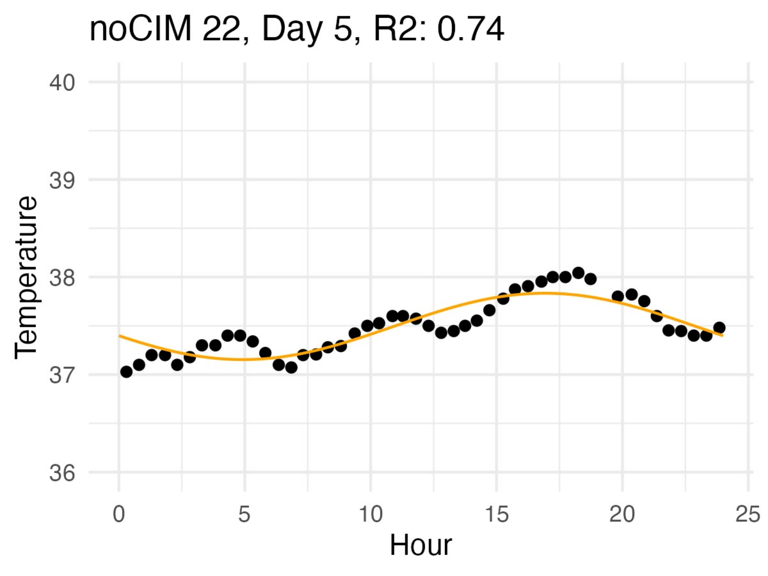

noCIM 22, Day 5, R2: 0.74
40
39
38
37
36
Temperature
0
5
10
15
20
25
Hour

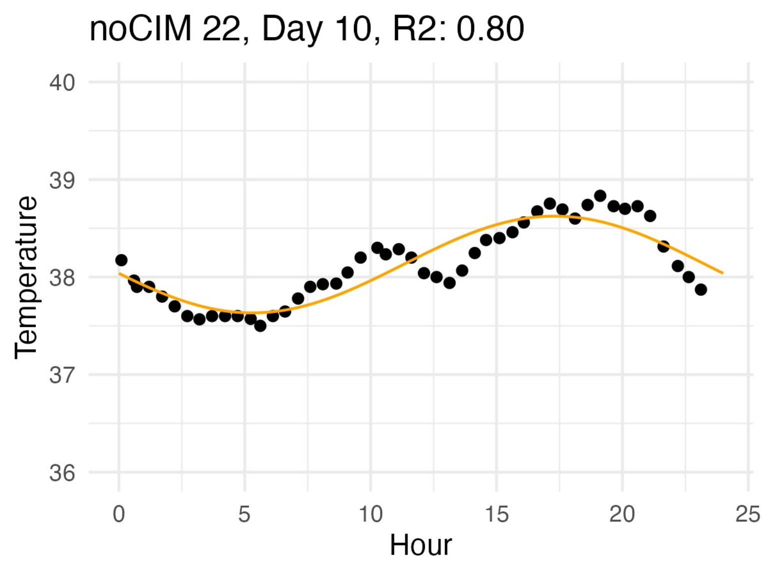
noCIM 22, Day 10, R2: 0.80
Temperature
40
39
38
37
36
0
5
10
15
20
25
Hour

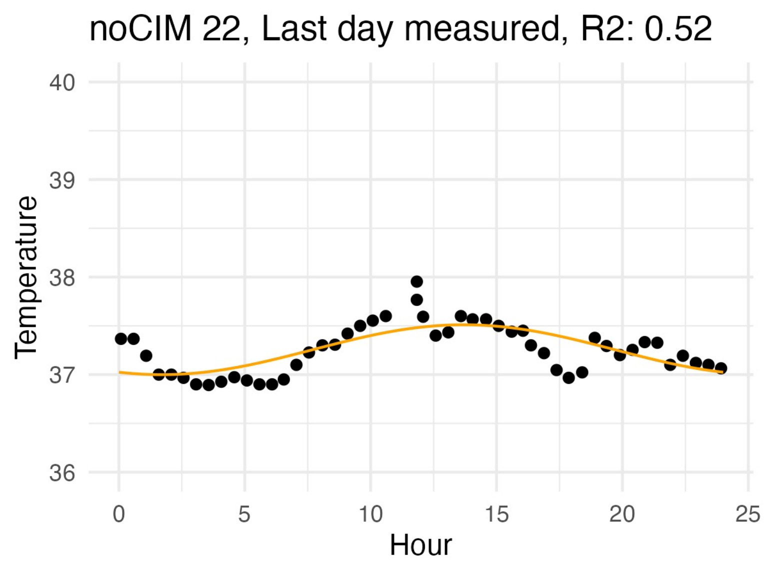
noCIM 22, Last day measured, R2: 0.52
Temperature
40
39
38
37
36
0
5
10
15
20
25
Hour

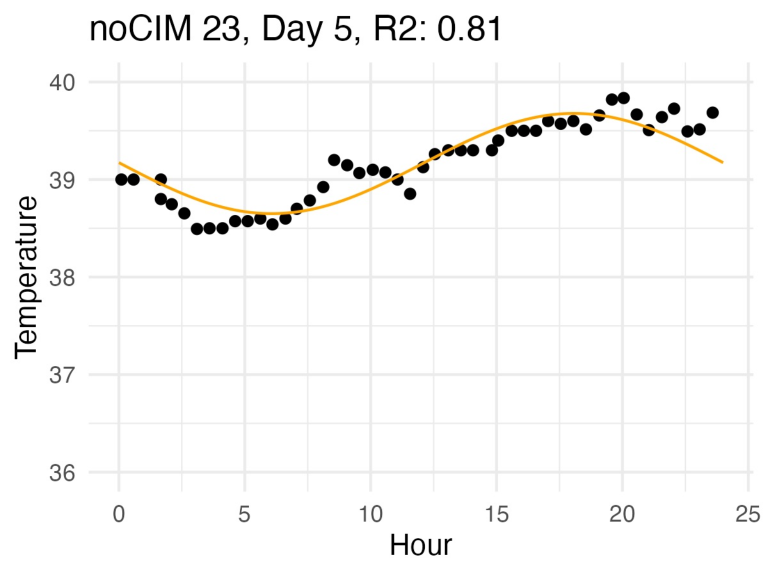
noCIM 23, Day 5, R2: 0.81
Temperature
36
37
38
39
40
Hour
0
5
10
15
20
25

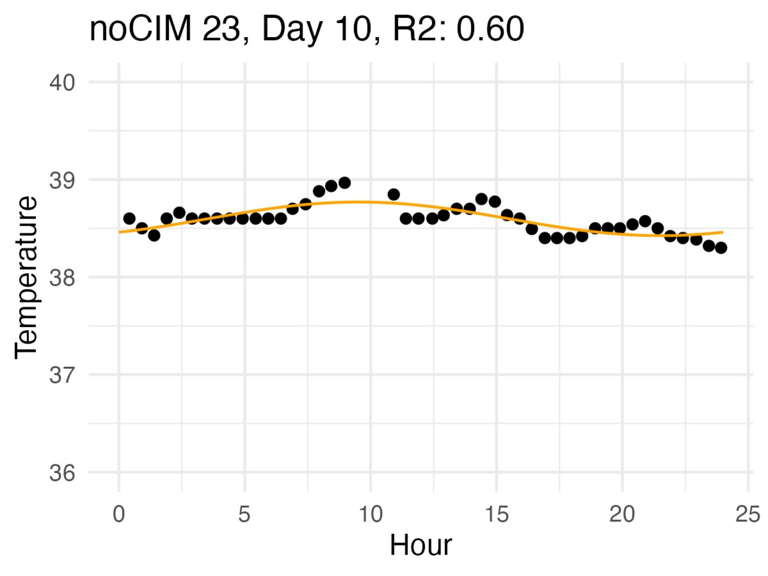
noCIM 23, Day 10, R2: 0.60
Temperature
40
39
38
37
36
0
5
10
15
20
25
Hour

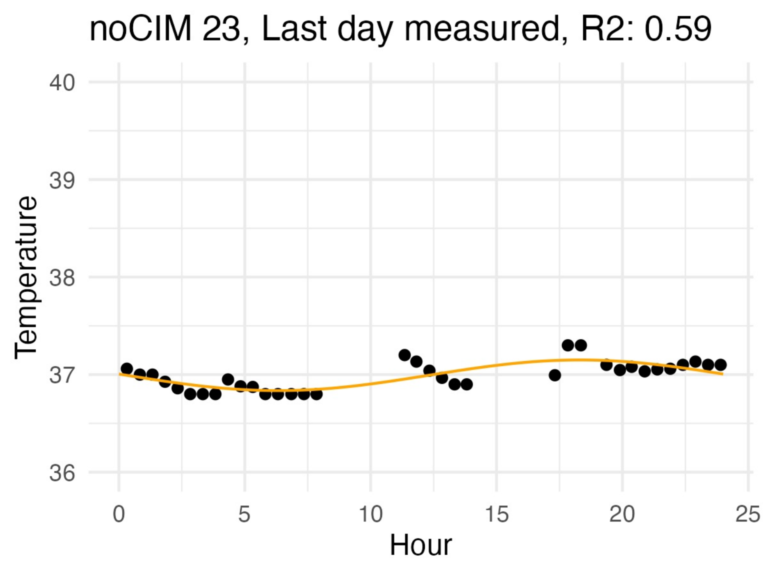
noCIM 23, Last day measured, R2: 0.59
Temperature
40
39
38
37
36
0
5
10
15
20
25
Hour

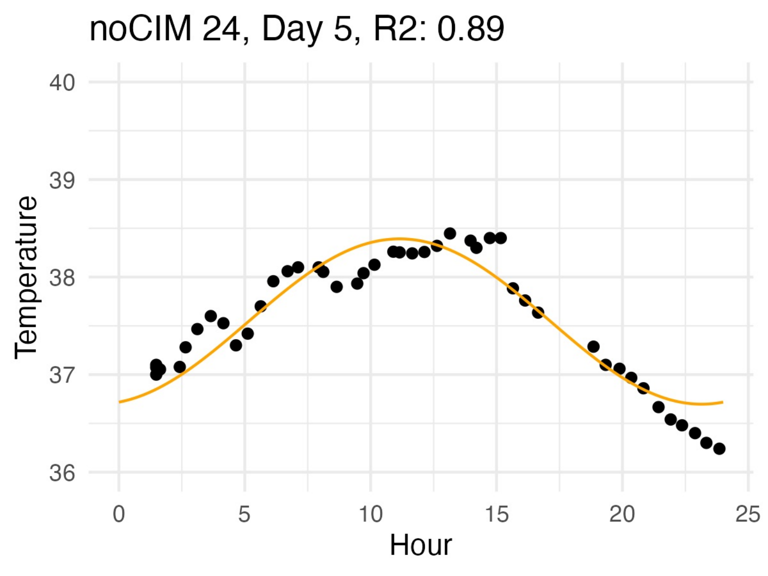
noCIM 24, Day 5, R2: 0.89
Temperature
40
39
38
37
36
Hour
0
5
10
15
20
25

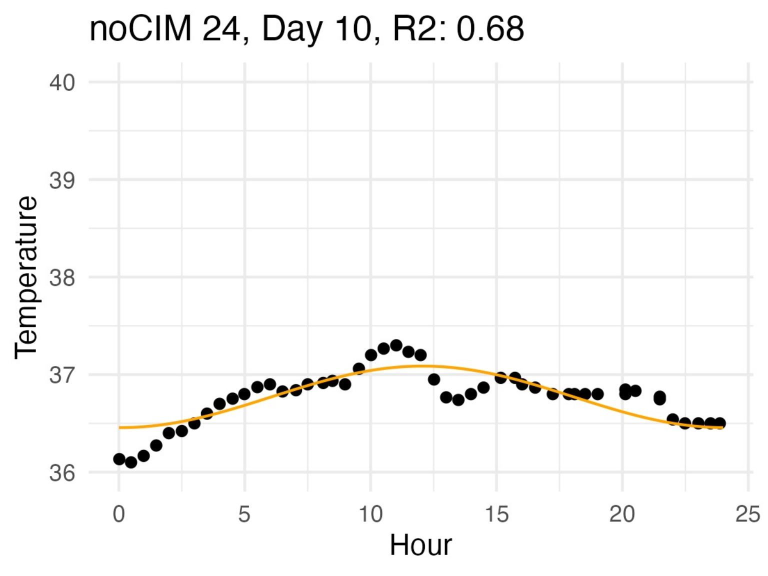
noCIM 24, Day 10, R2: 0.68
Temperature
40
39
38
37
36
0
5
10
15
20
25
Hour

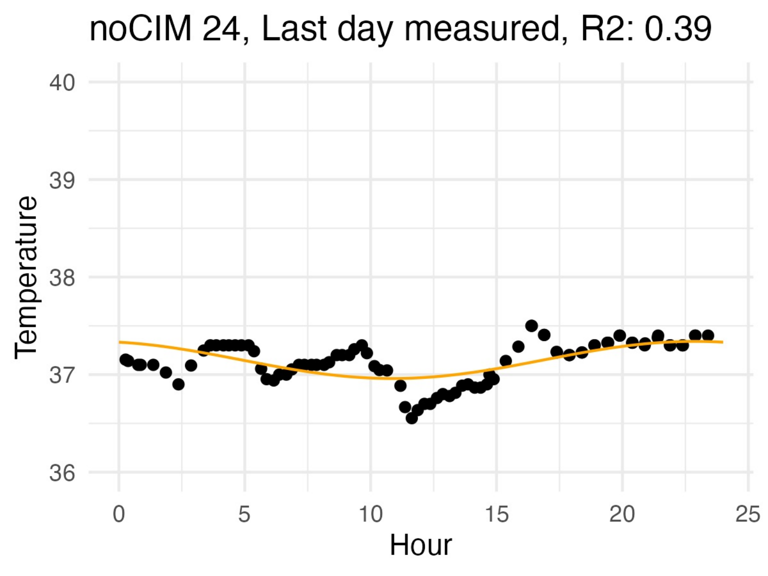
noCIM 24, Last day measured, R2: 0.39
Temperature
40
39
38
37
36
0
5
10
15
20
25
Hour

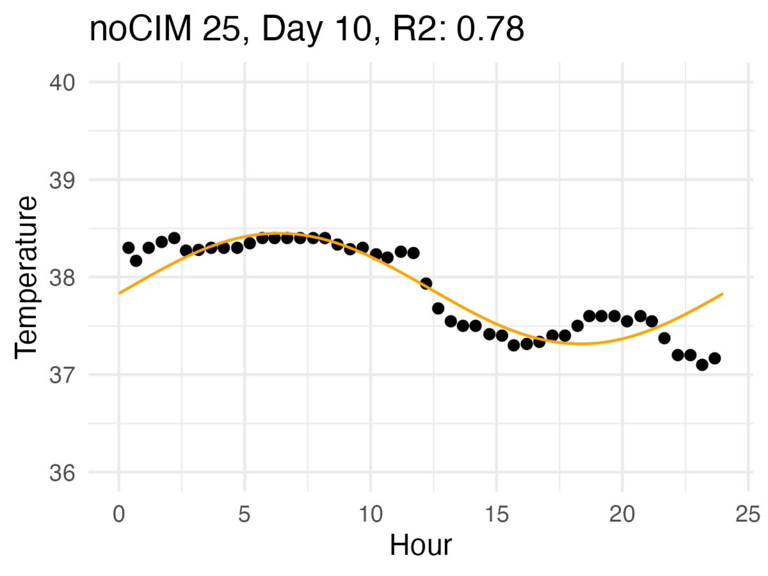

noCIM 25, Day 10, R2: 0.78
Temperature
40
39
38
37
36
0
5
10
15
20
25
Hour

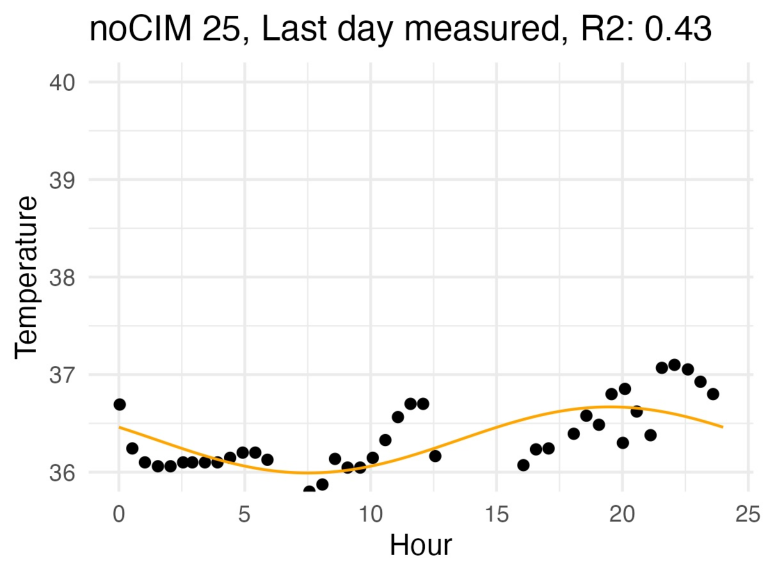
noCIM 25, Last day measured, R2: 0.43
Temperature
40
39
38
37
36
Hour
0
5
10
15
20
25

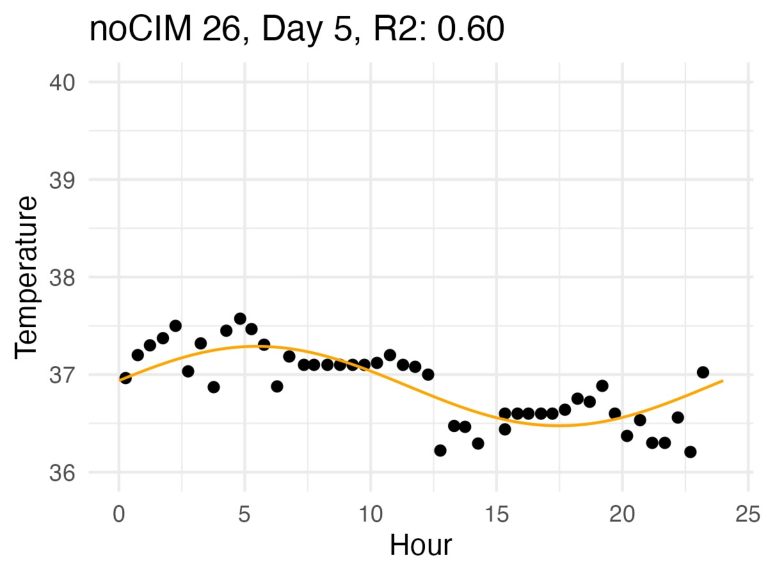
noCIM 26, Day 5, R2: 0.60
Temperature
Hour
40
39
38
37
36
0
5
10
15
20
25

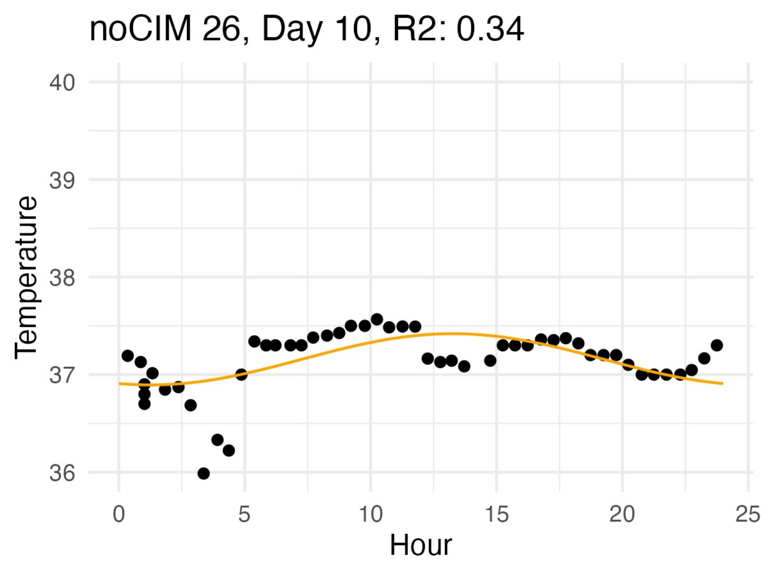
noCIM 26, Day 10, R2: 0.34
Temperature
40
39
38
37
36
0
5
10
15
20
25
Hour

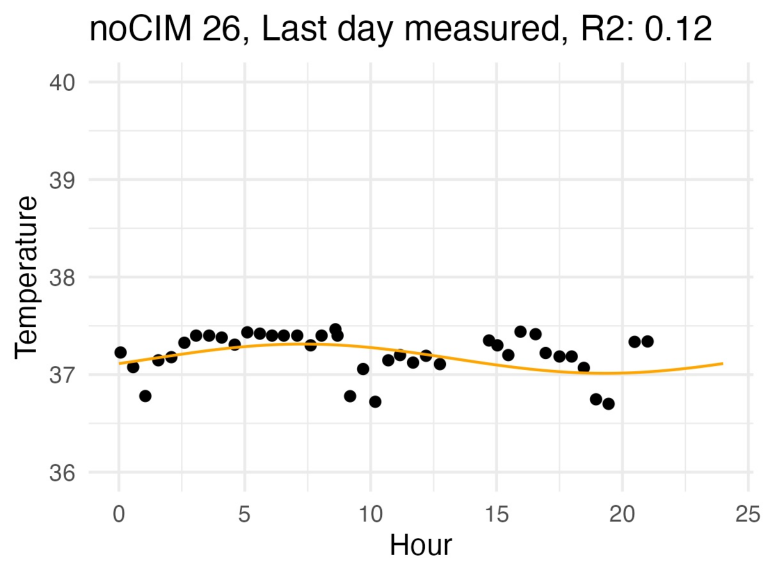
noCIM 26, Last day measured, R2: 0.12
Temperature
40
39
38
37
36
0
5
10
15
20
25
Hour

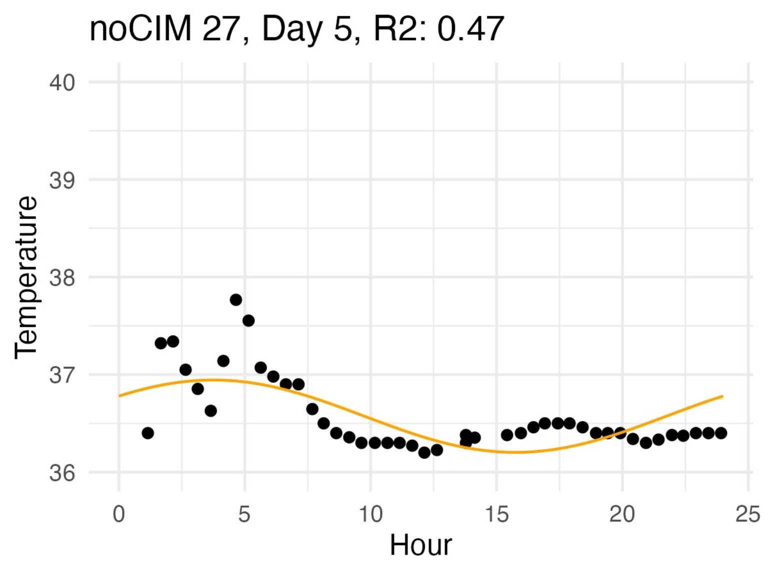
noCIM 27, Day 5, R2: 0.47
Temperature
40
39
38
37
36
0
5
10
15
20
25
Hour

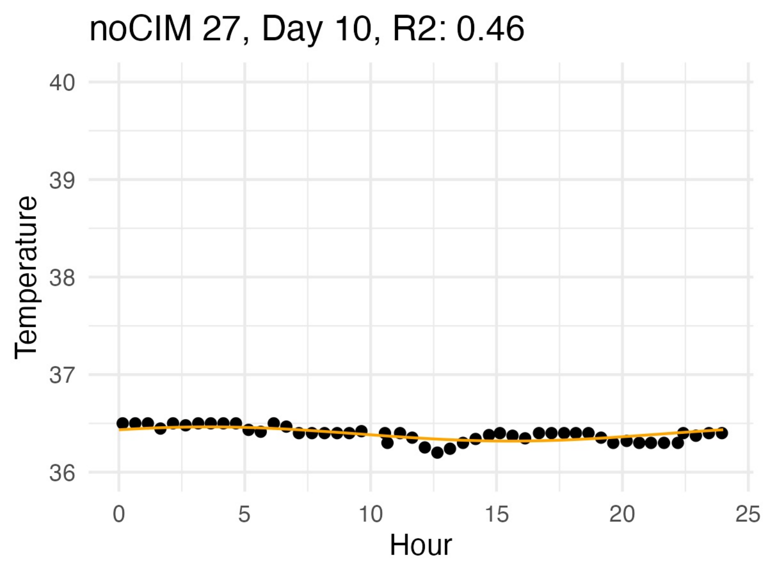

noCIM 27, Day 10, R2: 0.46
Temperature
40
39
38
37
36
0
5
10
15
20
25
Hour

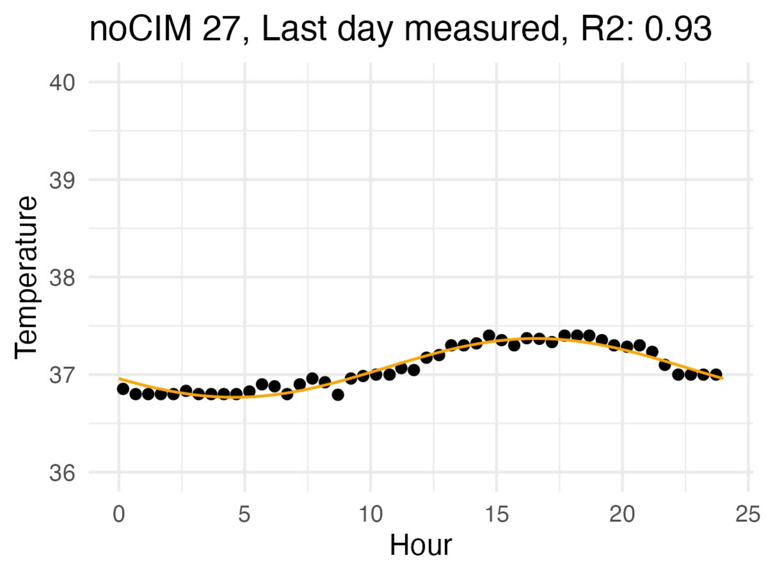
noCIM 27, Last day measured, R2: 0.93
40
39
38
37
36
Temperature
0
5
10
15
20
25
Hour

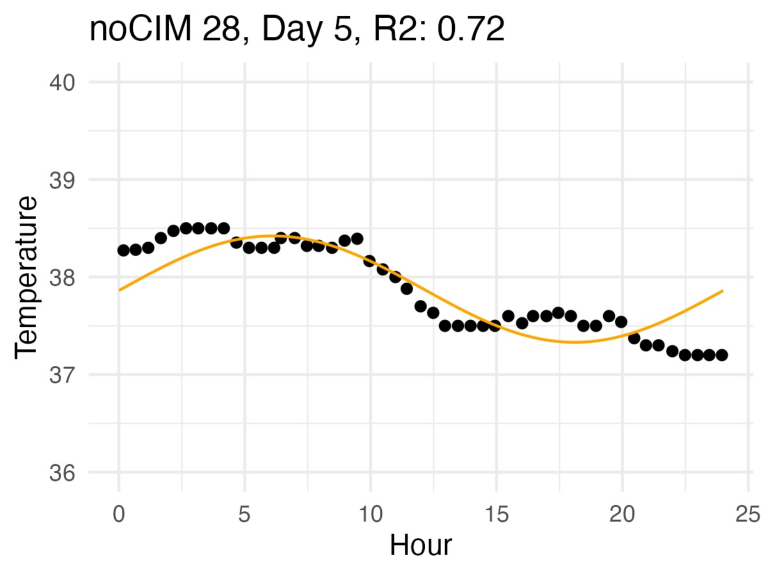
noCIM 28, Day 5, R2: 0.72
Temperature
40
39
38
37
36
0
5
10
15
20
25
Hour

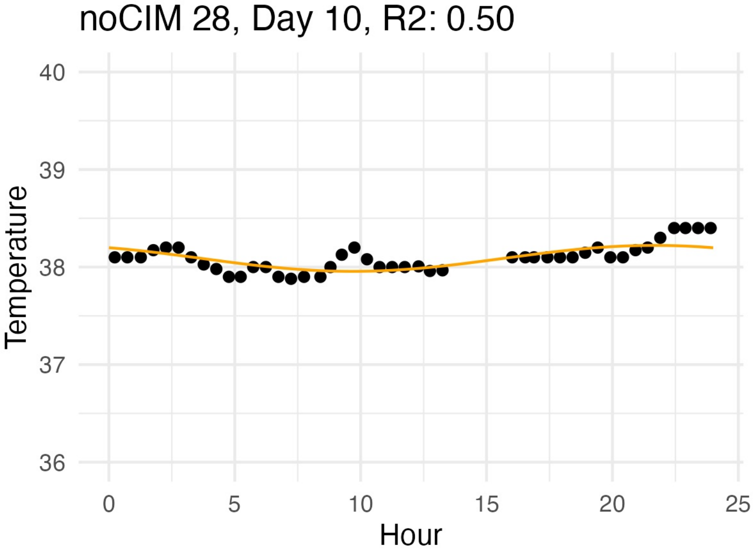
noCIM 28, Day 10, R2: 0.50
Temperature
40
39
38
37
36
0
5
10
15
20
25
Hour

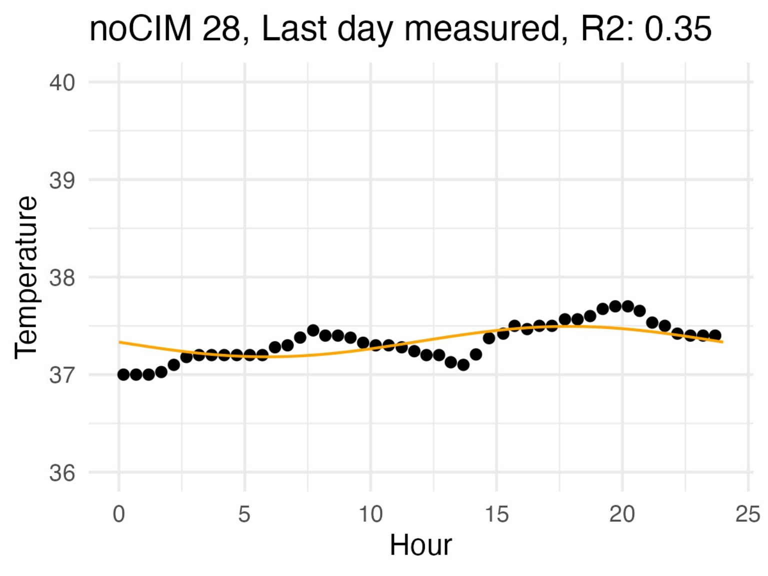
noCIM 28, Last day measured, R2: 0.35
Temperature
40
39
38
37
36
0
5
10
15
20
25
Hour

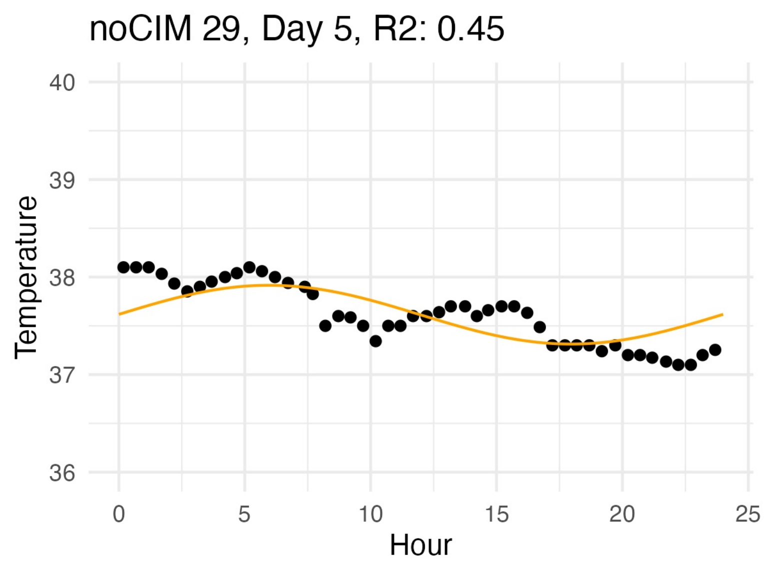
noCIM 29, Day 5, R2: 0.45
Temperature
40
39
38
37
36
0
5
10
15
20
25
Hour

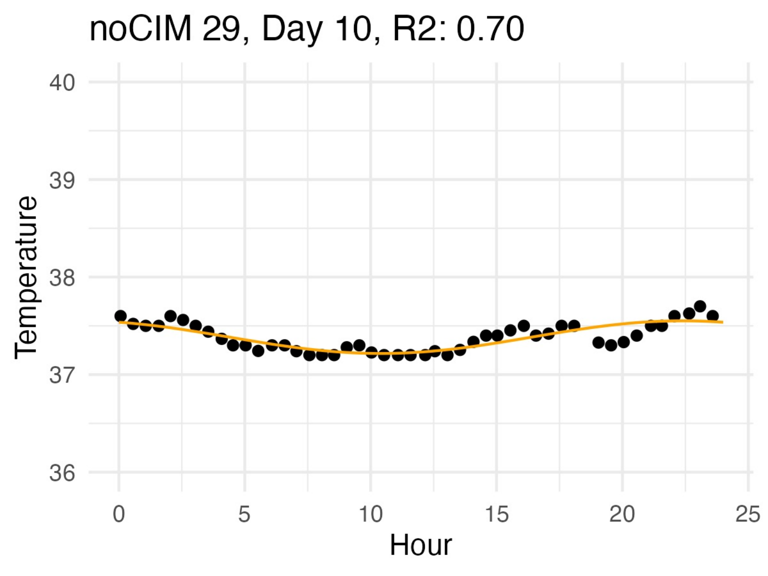
noCIM 29, Day 10, R2: 0.70
Temperature
40
39
38
37
36
0
5
10
15
20
25
Hour

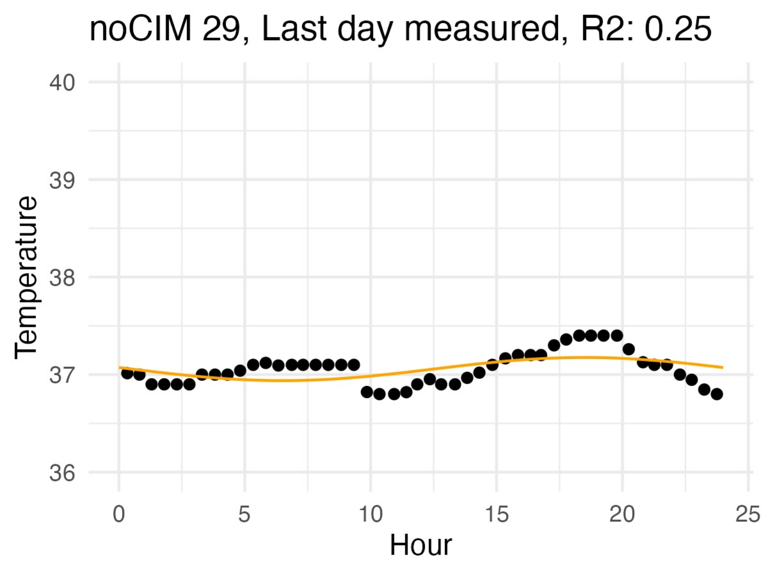

noCIM 29, Last day measured, R2: 0.25
Temperature
Hour
40
39
38
37
36
0
5
10
15
20
25

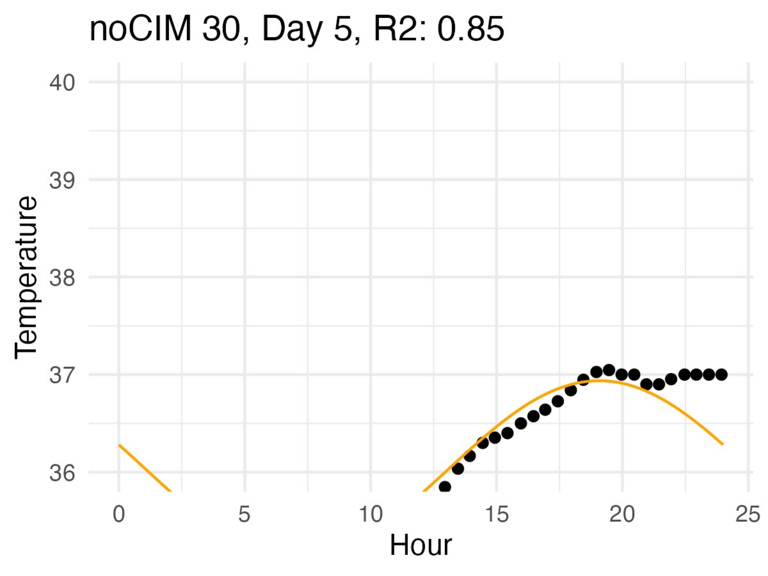

noCIM 30, Day 5, R2: 0.85
40
39
38
37
36
Temperature
0
5
10
15
20
25
Hour

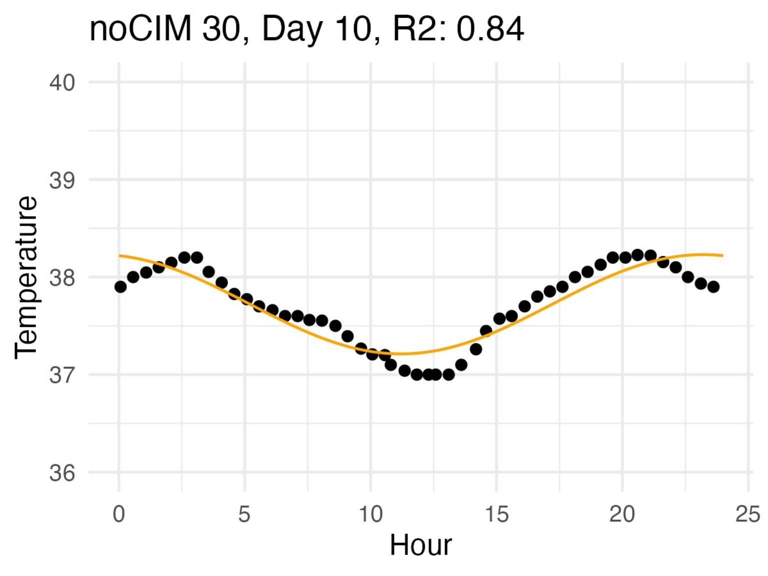
noCIM 30, Day 10, R2: 0.84
Temperature
Hour
40
39
38
37
36
0
5
10
15
20
25

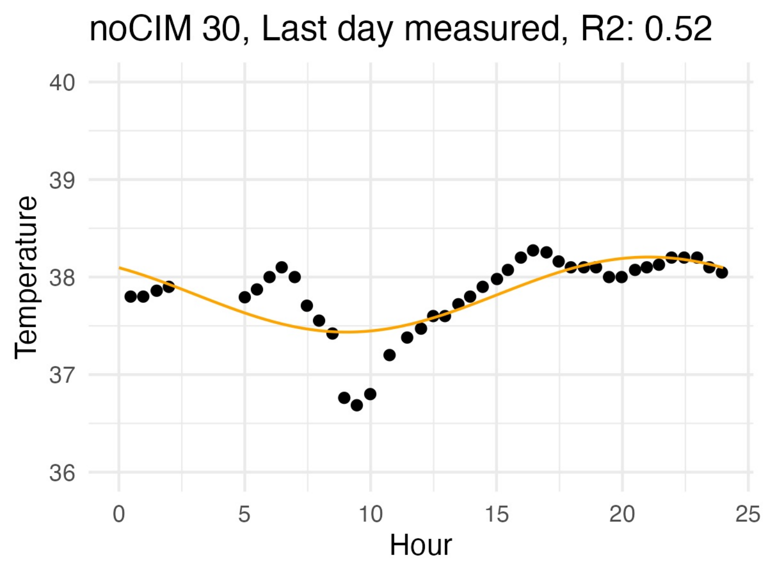
noCIM 30, Last day measured, R2: 0.52
Temperature
40
39
38
37
36
0
5
10
15
20
25
Hour
